# A transcriptomic examination of encased rotifer embryos reveals the developmental trajectory leading to long-term dormancy; are they “animal seeds”?

**DOI:** 10.1186/s12864-024-09961-1

**Published:** 2024-01-27

**Authors:** Tamar Hashimshony, Liron Levin, Andreas C. Fröbius, Nitsan Dahan, Vered Chalifa-Caspi, Reini Hamo, Oshri Gabai-Almog, Idit Blais, Yehuda G. Assaraf, Esther Lubzens

**Affiliations:** 1 Faculty of Biology, Technion-Israel Institute of Technology, Haifa, Israel; 2 National Institute of Biotechnology in the Negev, Ben-Gurion University of the Negev, Beer-Sheva, Israel; 3 Molecular Andrology, Biomedical Research Center Seltersberg (BFS), Justus Liebig University Gießen, Gießen, Germany; 4 Interdisciplinary Center for Life Sciences and Engineering, Technion-Israel Institute of Technology, Haifa, Israel; 5 Department of Obstetrics and Gynecology, Division of Reproductive Endocrinology and IVF, Lady Davis Carmel Medical Center, Haifa, Israel; 6 The Fred Wyszkowski Cancer Research Laboratory, Faculty of Biology, Technion-Israel Institute of Technology, Haifa, Israel; 7 (Retired) Israel Oceanographic and Limnological Research, Haifa, Israel

**Keywords:** Long-term dormancy, Transcriptome, Encased embryos, Embryogenesis, Rotifer

## Abstract

**Background:**

Organisms from many distinct evolutionary lineages acquired the capacity to enter a dormant state in response to environmental conditions incompatible with maintaining normal life activities. Most studied organisms exhibit seasonal or annual episodes of dormancy, but numerous less studied organisms enter long-term dormancy, lasting decades or even centuries. Intriguingly, many planktonic animals produce encased embryos known as resting eggs or cysts that, like plant seeds, may remain dormant for decades. Herein, we studied a rotifer *Brachionus plicatilis* as a model planktonic species that forms encased dormant embryos via sexual reproduction and non-dormant embryos via asexual reproduction and raised the following questions: Which genes are expressed at which time points during embryogenesis? How do temporal transcript abundance profiles differ between the two types of embryos? When does the cell cycle arrest? How do dormant embryos manage energy?

**Results:**

As the molecular developmental kinetics of encased embryos remain unknown, we employed single embryo RNA sequencing (CEL-seq) of samples collected during dormant and non-dormant embryogenesis. We identified comprehensive and temporal transcript abundance patterns of genes and their associated enriched functional pathways. Striking differences were uncovered between dormant and non-dormant embryos. In early development, the cell cycle-associated pathways were enriched in both embryo types but terminated with fewer nuclei in dormant embryos. As development progressed, the gene transcript abundance profiles became increasingly divergent between dormant and non-dormant embryos. Organogenesis was suspended in dormant embryos, concomitant with low transcript abundance of homeobox genes, and was replaced with an ATP-poor preparatory phase characterized by very high transcript abundance of genes encoding for hallmark dormancy proteins (e.g., LEA proteins, sHSP, and anti-ROS proteins, also found in plant seeds) and proteins involved in dormancy exit. Surprisingly, this period appeared analogous to the late maturation phase of plant seeds.

**Conclusions:**

The study highlights novel divergent temporal transcript abundance patterns between dormant and non-dormant embryos. Remarkably, several convergent functional solutions appear during the development of resting eggs and plant seeds, suggesting a similar preparatory phase for long-term dormancy. This study accentuated the broad novel molecular features of long-term dormancy in encased animal embryos that behave like “animal seeds”.

**Supplementary Information:**

The online version contains supplementary material available at 10.1186/s12864-024-09961-1.

## Introduction

Organisms from many distinct evolutionary lineages have acquired the capacity to enter a dormant state in response to environmental conditions incompatible with maintaining normal life activities. Upon resumption of favorable environmental conditions, the dormant organism re-initiates development and reproduction. Dormancy (also known as diapause in many organisms) is of immense ecological and evolutionary importance. It assures survival through unfavorable conditions by synchronizing the organism’s life cycle with the seasonality of abiotic environmental factors and biotic interactions.

Types of diapause, which may be periodic or annual and last several weeks or months, include the dauer stage, hibernation, and aestivation. In many cases, diapause is facultative, such that animals may undergo either a diapause or a non-diapause developmental trajectory, depending on environmental cues and the specific characteristics of any given species, and is genetically determined. The developmental trajectory of diapause or dormancy includes a preparatory phase, a dormant phase, a recovery phase, and a period of post-dormancy development [1–3]. Seasonal or short-short term diapause was reported in many taxa, including nematodes (e.g., the dauer stage of *Caenorhabditis elegans;* reviewed in [2, 4]), insects [3, 5], post-embryonic copepods [6], and annual killifish embryos [7]. In addition, over 130 mammalian species exhibit diapause during embryogenesis [8]. Insects, snails, fish, frogs and toads, may exhibit aestivation [9].

In contrast to these short dormancy periods, some organisms remain dormant for decades or centuries as encased embryos [10–15]. For example, embryos with arrested development are well-known in aquatic invertebrate taxa such as the *Daphnia*, the brine shrimp *Artemia*, copepods, and rotifers. These organisms play an essential role in the aquatic food web and aquaculture. The egg banks they form in sediments are essential for establishing future generations [16–18]. Long-term dormancy is similarly well known in plants: plant seeds survive for centuries [19, 20].

This phenomenon, whereby organisms enter a dormant state and survive very long periods in a state of suspended animation, challenges our concept of what constitutes life. This biological puzzle has captured the imaginations of scientists [18, 21, 22] but is far from being resolved. In general, entrance into dormancy occurs under conditions conducive to normal development. Dormancy triggers vary among taxa; known cues include changes in photoperiod, crowding, temperature, and salinity [2, 3, 5, 17, 23]. In several aquatic invertebrates, the triggers lead to a change from asexual to sexual reproduction, resulting in the formation of encased dormant embryos in the form of so-called resting eggs (e.g., rotifers and copepods), cysts (e.g., *Artemia*), or ephippia (e.g., *Daphnia*). In these examples, the embryos enter dormancy via the suspension of the cell cycle after the blastula or gastrula stage [6, 24–26].

Molecular analyses of diapause were performed in several taxa, including *C. elegans* (dauer larval stages; [4]): insect model and near-model species such as *Drosophila melanogaster* (reproductive diapause), the silkworm *Bombyx mori* (embryos and pupae), and the mosquitoes *Culex pipiens* and *Aedes aegypti* (reproductive diapause); copepods (post-embryonic stages); and annual killifish (embryo diapause; reviewed in [2, 3, 6]). These species exhibit dormancy lasting for several weeks or months. Studies of species exhibiting long-term embryonic dormancy include those on the brine shrimp *Artemia* [27–32] copepods [6, 18], and the rotifer *Brachionus* [17]. Remarkably, despite the diversity and complexity of survival strategies used by organisms capable of dormancy, similar functional pathways are associated with this phenomenon [1, 3, 27, 33, 34], including the suspension of the cell cycle, repression of metabolic pathways, changes in the carbohydrate and lipid metabolism, increased resistance to stress, and protection of proteins and cellular structures. Although there is little similarity in dormancy-related transcriptional profiles across species [35], recent studies have identified a core set of genes that are differentially regulated during diapause in insect species [36]. Common diapause-related functional themes have begun to be identified in three animal phyla: nematodes (*Caenorhabditis elegans*), insects (*Drosophila melanogaster* and *Culex pipiens*), and vertebrates (the annual killifish *Austrofundulus limnaeus* and *Nothobranchius furzeri*). They showed the involvement of insulin, insulin-like growth factor (IGF) signaling (IIS), and the FoxO transcription factor. Moreover, diapause-associated nuclear hormone receptors (NHs) were similar across nematodes, insects, and vertebrates, including DAF-12 in *C. elegans*, the ecdysone receptor (EcR) in insects, and the vitamin D receptor (VDR) in the annual killifish [2, 3, 37].

Numerous signaling pathways, including MAPK, Hippo, and Wnt, are associated with diapause in various organisms [34]. For example, the TOR, JAK/STAT, and JNK signaling pathways affected adult diapause in *Drosophila* [38, 39]. In contrast, the Insulin/IGF, Insulin/FoxO, and TGFβ signaling pathways were involved in entering dauer diapause and insect diapause [1, 40]. The LKB1/AMPK pathway was implicated in the rationing of lipid reserves [41], and TGFβ and BMP signals regulated pupal diapause in the cotton bollworm [42]. In addition, mTOR regulated diapause in the mouse embryonic model [8, 43, 44]. In *Artemia*, pathways that may govern the induction and maintenance of mitotic arrest during diapause include the AURKA-PLK-MEK-ERK-RSK, LKB1-AMPK-CFTR-p53, and Wnt signaling pathways, which are simultaneously required for the regulation of cell-cycle arrest [1, 30]. In addition, a histone lysine methyltransferase from *Artemia* (Ar-SETD4) regulated the trimethylated histone H4K20 (H4K20me3) gene expression level. The knockdown of Ar-SETD4 reduced the level of H4K20me3 significantly and prevented the formation of diapause embryos [45]. Finally, there is evidence that noncoding RNAs (miRNAs, siRNAs, piRNAs, mitosRNAs, and lncRNAs) regulate dormancy in several organisms. They target genes of the TGFβ, mTOR, Wnt, and Insulin signaling pathways, which regulate dormant states [1, 34, 46].

Transcriptome analyses are essential for a clear understanding of molecular dynamics during the development of dormant embryos. However, almost all of the transcriptome studies have characterized transcriptomic changes during embryonic development in non-dormant embryos in various model and non-model organisms [47–51]. One of the few studies examining dormancy was performed on annual killifish embryos. Transcriptomics was performed on embryos at the 1–2-cell stage programmed for the diapause trajectory and differed from those of ontogenetically-matched embryos programmed for the trajectory lacking diapause. The identified differences included alternatively spliced mRNAs and distinct populations of small noncoding RNAs (sncRNAs; [52, 53]).

In this study, we aimed to investigate the mechanisms underlying long-term dormancy using an aquatic invertebrate, the rotifer *Brachionus plicatilis.* The *Brachionus* species complex is crucial to natural aquatic food web. *Brachionus* species are also used as live food for the larvae of farmed marine fish and as an indicator taxon in ecotoxicology studies [23]. Brachionid rotifers produce diploid amictic eggs (AMs), which are formed by oocytes that undergo a single equational maturation division or "mitosis". They also produce two types of mictic eggs: unfertilized haploid male eggs and diploid fertilized encased resting eggs (REs) that enter dormancy [17, 23, 54]. Continued amictic reproduction gives rise to genetically identical diploid amictic females. Oocytes produced by mictic females undergo meiosis, and the resulting haploid eggs are either fertilized, becoming diploid REs, or mature as haploid males, producing haploid sperm by equational maturation division.

In contrast to AMs, REs are encased in protective impermeable shell layers [55]. Although they are referred to as "eggs," REs and AMs are developing embryos that are carried by the maternal rotifer from the time of extrusion from her body. After extrusion, the females carries AMs and haploid male eggs until hatching (after 11–14 h of development), while REs are carried for 48 h, at which point they are released and sink to the substrate. The dark-colored REs contain maternal metabolome contribution which differs from AMs, that will serve the REs during dormancy and the subsequent revival period until hatching. REs continue to develop for several days after formation [56]. Then, they enter an obligatory dormant period (with some exceptions [57, 58]) that lasts at least several weeks [59]. REs retain viability for decades [12, 13] and resist desiccation [59]. After the obligatory dormant period, illumination induces the hatching of REs [56, 60]. The neonates hatched from REs are morphologically indistinguishable from those hatched from AMs.

To investigate the molecular mechanisms that characterize REs and dormancy in *B. plicatilis*, we explored differences in transcript abundance between REs and AMs during development and the initiation of dormancy in REs. Specifically, we used single-embryo RNA-sequencing (CEL-seq) to compare global transcript abundance patterns between dormant and non-dormant embryos sampled at hourly intervals from extrusion until hatching of AMs or until 192 h after extrusion of REs. We focused on differences in transcript abundance patterns between REs and AMs for signaling pathways, transcription factors, homeobox genes, lipid metabolism, phototransduction, circadian rhythm, and circadian entrainment pathways. We also investigated whether nuclear receptors associated with diapause in other organisms were implicated in RE dormancy. Finally, we determined in REs the points at which the cell cycle was suspended, and the ATP levels were reduced. Our results revealed an extended preparatory period of diapause in REs, accompanied by a high transcript abundance of genes encoding for proteins that are hallmarks of dormancy in many organisms and during seed maturation (e.g., LEA, sHSP, and anti-ROS proteins). These proteins facilitate the long-term survival of plant seeds [61]. Like patterns observed in plant seeds, the dormancy preparation period in the REs was characterized by the relatively high transcript abundance of genes with putative functions at the exit from dormancy. Our results enhance our understanding of the global and temporal molecular functions underlying dormancy initiation and illuminate novel dormancy preparation processes that putatively facilitate the long-term survival of the encased embryos of aquatic species.

## Results and discussion

### Physiological changes during embryonic development

There was an apparent morphological difference between the two types of rotifer eggs at the time of extrusion: the AMs were translucent, while the REs were slightly larger and dark-colored. Cellular movements were observed during early development and organogenesis in the AMs (0–12 h post-extrusion, PE), including forming of the frontal lobes, mastax, and eyes just before hatching. Between 0 and 48 h PE in the REs, the most notable visual change occurred at ~ 12 h PE with the regression of the membrane enclosing the cytoplasmic mass from the external egg coverings during the formation of the fluid-filled extra-embryonic space in which the embryo is enclosed [55]. After this point, the embryo changed from dark brown or almost black to medium brown (Additional file 1, S1A Video (RE); Additional file 2, S1 Table). Additional cytoplasmic movements were apparent in the REs at 36–48 h PE. In rotifer cultures, AMs hatch during 11–14 h PE while still attached to the maternal female. The REs are released by the maternal females at ~ 48 h PE and sink to the sediment.

**Fig. 1:**
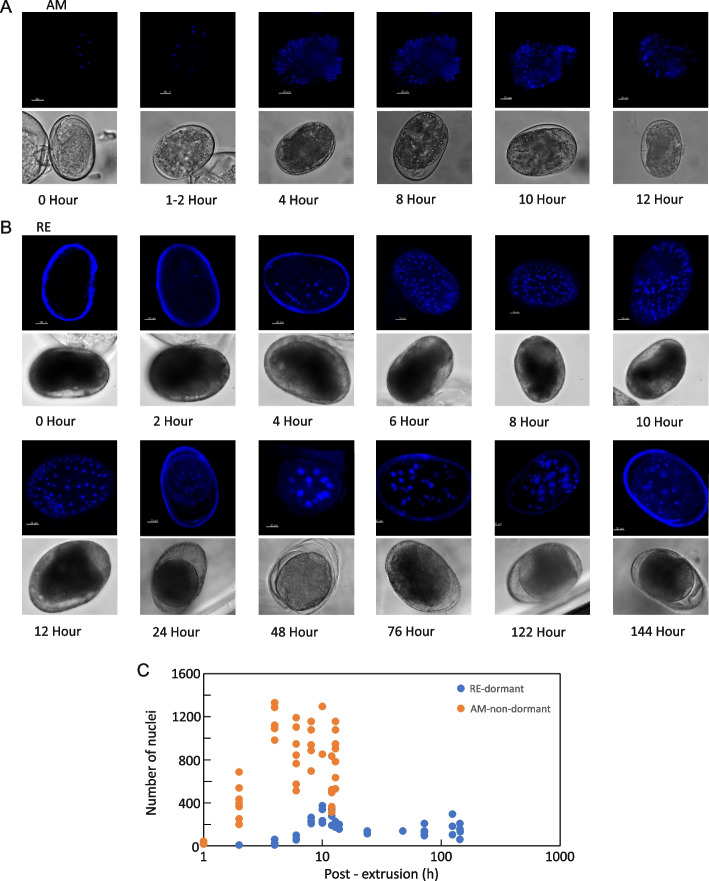
The number of nuclei during the development of dormant (RE) and non-dormant (AM) embryos. Hoechst 33342-stained (top panels) and bright-field (bottom panels) photomicrographs of representative (**A**) AMs and (**B**) REs at various developmental time points. **C** The number of nuclei throughout AM and RE development. Due to the formation of an impenetrable membrane by the REs at ~ 12 h PE, REs were decapsulated before staining from 15 h PE onwards

Interestingly, AMs were permeable to the viable DNA dye Hoechst at all developmental stages. However, REs became impermeable to this DNA dye from ~ 12 h PE, suggesting that the impermeable membrane had enveloped the embryo [55]. For visualization purposes, these REs were decapsulated before staining [62], which may have affected the visualization of nuclei. The number of nuclei in the developing AMs increased gradually to ~ 1,000 after 6–8 h PE (Fig. 1A and C), representing ten cycles of nuclei division. In contrast, the number of nuclei in the REs, was much lower, increasing to only ~ 150–200 by 8–10 h PE (Fig. 1B and C), representing ~ 7 cleavage cycles; the number of nuclei then remained stable throughout dormancy. Notably, the duration of the cell cycle in AMs was shorter than that of REs, as discussed below. The number of nuclei in encased embryos at the stage of developmental arrest seems to vary among taxa. It ranges from 32 in marine copepods (blastula stage; reviewed in [6]), to ~ 1,000 and 3,500 in *Daphnia pulex* and *D. magna,* respectively (early gastrula; [26]), and 4,000 in *Artemia* sp. (gastrula; [24]). In addition, previous studies with *B. plicatilis* reported much lower numbers of nuclei in the REs (26 nuclei in [56]; 160 nuclei in [25]); these differences in the number of observed nuclei may stem from the diverse methods used for nuclear visualization. After 24 h PE, some of the nuclei in the REs were much larger than others; these large nuclei may be associated with the central structures that were observed in vivo [(Fig. 1B; last frames of Additional file 1, S1A Video (RE)]. It has been proposed that the central core of the RE is composed of large nuclei surrounded by an external layer of small nuclei [25]. However, it remains unknown how these large nuclei are formed. Rotifers have a fixed number of cells (eutely) at hatching [63] and a syncytial body plan. Two or three additional doublings, followed by organogenesis, must occur in the REs at the time of dormancy exit before hatching, as the neonates hatching from AMs and REs are morphologically and functionally similar.

In most organisms, the early rapid embryonic cell cycles rely on maternally contributed components [64–70]. The cleavage cycles end with an abrupt transition that is followed by the onset of gastrulation and is known as the mid-blastula transition (MBT). Following a period of transcriptional quiescence, zygotic transcription commences, and the maternal control begins to decline. This process is known as the maternal-to-zygotic transition (MZT; [66]). The expression of the maternal genes is replaced by that of the corresponding genes in the zygote genome. The MZT is evolutionarily conserved across different branches of metazoa, albeit the timing of the initiation of zygotic transcription varies among species and may start after fertilization [66]. In rotifers, gastrulation occurs at the 16-cell stage [23], which is reached at ~ 1 h PE in AMs and ~ 2–4 h PE in REs. The exact timing of the MZT transition has not been directly determined in the fertilized REs but is irrelevant for the unfertilized AMs.

### Transcriptome analysis

To construct the developmental transcriptome, we collected AMs and REs throughout development: AMs were collected hourly for 14 h, while REs were collected hourly for the first 12 h, and then at longer intervals up to 192 h, see Materials and Methods). At each time point, 5–7 embryos were collected. The 155 collected samples were sequenced using the CEL-seq protocol (see Materials and Methods; for a detailed description, see Additional file 3, S1 Text), generating 136 high-quality transcriptomes. In total, 14,779 genes were transcribed across all samples (transcript abundance after normalization and log2 transformation, are shown in Additional file 2, S2A Table). Of these genes, 63.7% (9,418 genes) were successfully annotated: 61.3% (9,061 genes) were associated with one or more gene ontology (GO) terms, and 42.6% (6,289 genes) were assigned a KEGG Orthology (KO) identifier. During the first 12 h of development, the transcript abundance patterns of 8,058 and 7,381 annotated genes changed significantly in the AMs and the REs, respectively. In addition, the transcript abundance patterns of 6,568 of the annotated genes differed between AMs and REs (dataset AM vs. RE) during this period.

**Fig. 2:**
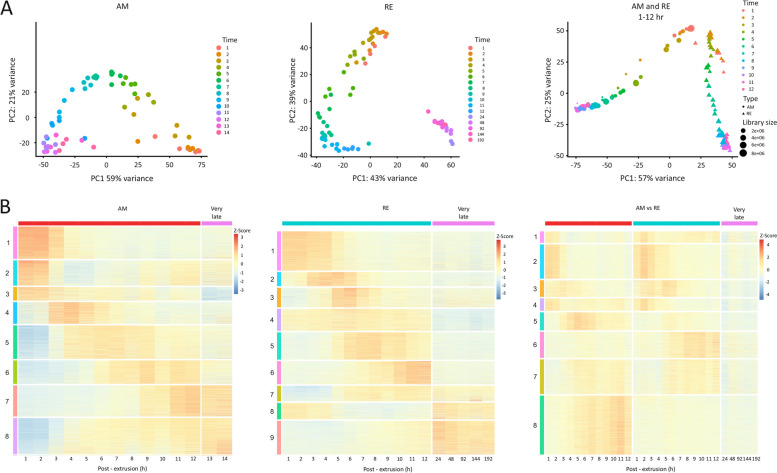
Temporal differential transcript abundance profiles for genes during AM development (0–14 h PE), during RE development (0–192 h PE), and between AMs and REs (0–12 h PE). **A** Principal component analysis (PCA) of the transcript abundance patterns. **B** The corresponding heatmaps show the temporal transcript abundance patterns of eight gene clusters during AM development, nine during RE development, and eight between AMs and REs

Principal component analysis (PCA; https://bioconductor.org/packages/release/bioc/html/scater.html) revealed a smooth progression through development for both AMs and REs (Fig. 2A), with 80% of the variation, in the normalized transcript abundance, explained by PC1 and PC2 for AMs and 82% of this variation explained by PC1 and PC2 for REs. The PCA of RE transcript abundance highlighted a distinction between transcript abundance patterns earlier in development (1–12 h PE) and much later in development (24–192 h PE). PCA of the genes with differential transcript abundance between AMs and REs over the first 12 h of development (1–12 h PE) revealed a noticeable divergence between AM and RE genes, which increased as development progressed. In this PCA, 57% of the variation in transcript abundance was explained by PC1 and 25% by PC2 (Fig. 2A).

**Fig. 3:**
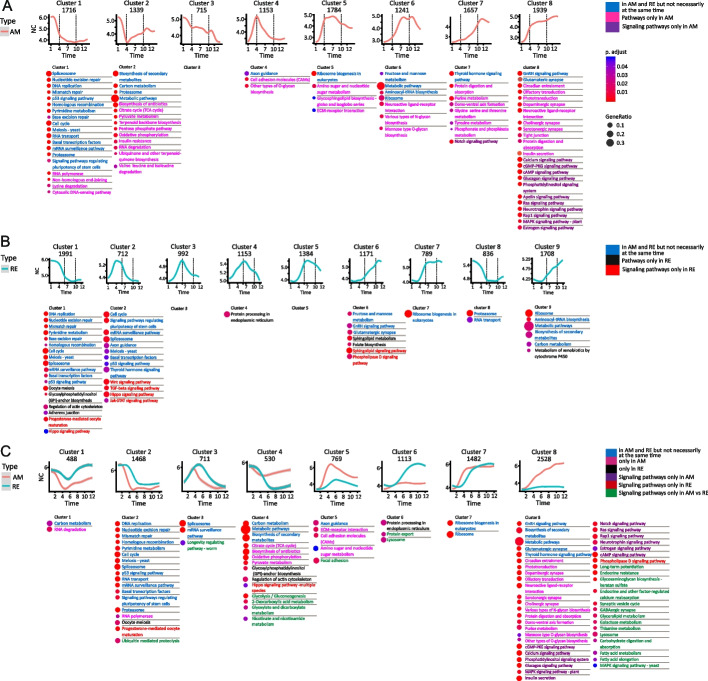
Transcript abundance patterns of each differential gene transcript cluster and the KEGG pathways enriched in each cluster. **A** AM clusters, (**B**) RE clusters, and (**C**) AM vs. RE clusters. In each panel, the upper images show the transcript abundance patterns of each cluster over time (h PE), and the associated enriched KEGG pathways are shown below. The name of each pathway is colored to indicate its specificity (AM, RE, or both but not necessarily simultaneously), and the colored circles prepending each pathway indicate the statistical significance of the enrichment (color) and the relative proportion of total genes (size)

Clustering analysis grouped the differential transcript abundance of genes for AM and RE development into eight and nine clusters, respectively, whereas genes with significantly different temporal transcript abundance patterns between AMs and REs were divided into eight clusters (Fig. 2B; Additional file 2, S3 Table). Examination of the KEGG pathways enriched in each cluster revealed significant functional differences during development (Fig. 3; Additional File 2, S3 Table). Visualization of transcript abundance patterns suggested that the first 12 h of development for both embryo types could be broadly divided into three phases (Figs. 2B and 3, Additional file 2, S3A–S3C Table): an early phase (AM: 1–3 h PE; RE: 1–5 h PE), a middle phase (AM: 4–9 h PE; RE: 6–9 h PE), and a late phase (RE and AM: 10–12 PE). A fourth phase, corresponding to very late development (AM: 13–14 h PE; RE: 24–192 h PE), was also discernible based on the transcript abundance patterns (Fig. 2B, Additional file 2, S3A–S3C Table).

### Molecular changes during embryonic development

#### The early phase of development

**Fig. 4:**
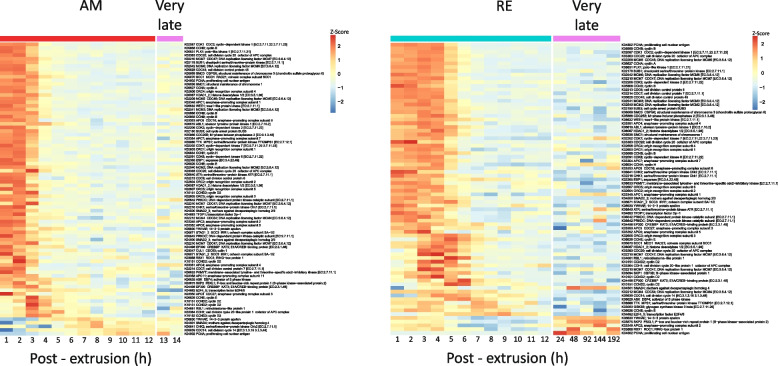
Heatmaps showing the temporal transcript abundance patterns of genes associated with the cell cycle pathway during development in AMs and REs

During early development (AM: 1–3 h PE; RE: 1–5 h PE), high transcript abundance was found of genes associated with the KEGG cell cycle, RNA translation, and related pathways (AM clusters 1 and 2; RE clusters 1, 2 and 8; Fig. 3). Notably, a similar number of genes was transcribed in AMs and REs (Additional file 4, S1 Fig). Most of the KEGG pathways enriched in the gene clusters during early development were shared between the AM and RE embryos; however, the associated gene transcripts were highly abundant only from 1–3 h PE in AMs, whereas in RE embryos, these gene transcripts were highly abundant up to 5 h after the beginning of development. Notably, gene transcripts associated with the cell cycle were with longer high abundance in the RE embryos than in AM embryos (Fig. 4). Interestingly, this transcript abundance pattern correlated well with an extended period of cell division in RE embryos (although nuclei division was likely slower in REs, as fewer cycles ultimately occurred; Fig. 1C). A few pathways were uniquely enriched in either AMs or REs: the unique AM pathways included RNA polymerase, non-homologous end-joining, lysine degradation, and cytosolic DNA-sensing pathway, while the unique RE pathways included oocyte meiosis, regulation of actin cytoskeleton, adherens junctions, progesterone-mediated oocyte maturation, and Hippo signaling. KEGG pathways and protein functions are described at https://www.genome.jp/pathway and https://www.uniprot.org/, respectively (see also Materials and Methods). Notably, the mode of function of progesterone in rotifers needs to be clarified, as the progesterone-mediated oocyte maturation pathway was enriched only in RE. A membrane progesterone receptor was identified in *B. manjavacas* [71], and progesterone increased the production of REs in *B. manjavacas* but not in *B. plicatilis* [72]. We identified two progesterone receptor genes in the *B. plicatilis* embryos normalized transcriptome (Additional file 2, S2A Table), the membrane progestin receptor β (c14530_g1) and the membrane progestin receptor γ (c22421_g2). The transcript abundance patterns of these two genes changed significantly over time during both AMs and REs development. However, significant differences in temporal transcript abundance patterns between AMs and REs were only identified for the membrane progestin receptor β (c14530_g1; Additional file 2, S2A Table), suggesting a specific yet unknown function, for progestin β in REs, at early developmental stages. In contrast to most genes, but like many others, the abundance of membrane progestin receptors β and γ transcripts was low at early time points in the progesterone-mediated oocyte maturation pathway.

The Hippo signaling pathway was enriched only in REs. Hippo signaling might be associated with the cell cycle arrest in these embryos as the Hippo pathway modulates cell proliferation, cell death, and cell differentiation to regulate cell number. The Hippo signaling pathway regulates stem-cell function, organ size, and tissue regeneration. Interestingly, the actin cytoskeleton pathway, which integrates and transmits upstream signals to the core Hippo signaling cascade and the adherens junction affecting the Hippo activity, were also enriched only in RE. These observations support a conjecture on the regulation of cell numbers in REs by the Hippo signaling pathway [73]. The TGFβ and Wnt signaling pathways which are associated with Hippo signaling (https://www.genome.jp/kegg-bin/show_pathway?map04390), were enriched in the REs simultaneously with Hippo signaling and the cell cycle, and related pathways. It should be noted that the genes associated with these pathways were also transcribed (but not enriched) in AMs, where the cell cycle continued until the full complement of nuclei was produced. The JAK-STAT signaling pathway, enriched only in the REs, transduces many signals for animal development and homeostasis (https://www.genome.jp/entry/map04630).

Notably, the proteasome and the RNA degradation pathways, which were enriched in AMs and REs, have specific functions during the MZT in embryos as they degrade maternal proteins and mRNAs, respectively [66, 69]. Interestingly, the genes transcript abundance of these two pathways was higher in REs compared with AMs (Fig. 3C, clusters 1 and 2). The higher transcript abundance at early time points (1–3 h PE in AM and 1–6 h PE in RE) supports the assumption regarding the degradation of proteins and mRNAs deposited during oogenesis [74].

**Fig. 5:**
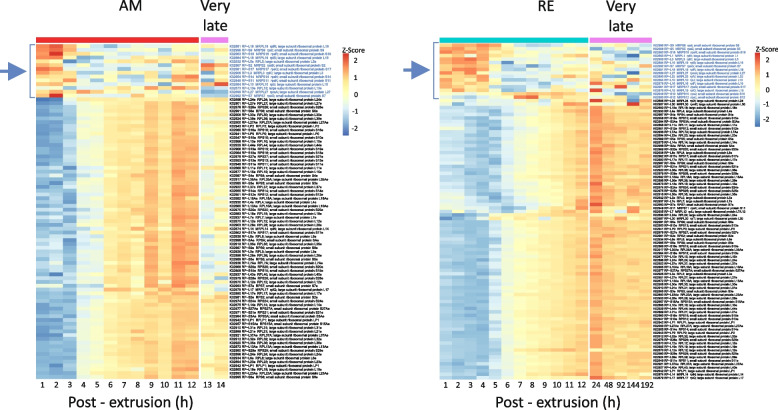
Heatmaps showing the temporal transcript abundance patterns of genes associated with the ribosome pathways during the development of AMs and REs. The blue arrow indicates the mitochondrial genes in the ribosome pathway, highlighting the differences in the transcript abundance patterns between nuclear and mitochondrial ribosomal genes. Of note is the high transcript abundance of ribosome genes during very late development in REs

A striking transcript abundance pattern during early development was observed for the genes encoding ribosomal proteins (Fig. 5). Genes encoding for nuclear ribosomes were not transcribed in both AMs and REs during the cell cycle, unlike mitochondrial ribosome transcripts, many of which showed high transcript abundance during this period. Maternal mRNAs drive the first mitotic cycles independently of mRNA transcription and ribosome biogenesis before the MZT [66], explaining the lack of ribosome transcripts at early developmental stages. Mitochondrial transcript abundance patterns may reflect the mitochondria’s continuous energy-generating activity [75]. Ribosome biogenesis and the ribosomal pathways were enriched at later developmental stages in AMs (from 4 h onwards; clusters 5 and 6, Fig. 3A) and RE (from 6 h onwards, clusters 4 and 9, Fig. 3B). Indeed, many ribosomal proteins have been identified in mature REs [76]. Our results were consistent with early studies demonstrating a lack of ribosomal RNA synthesis in numerous species before the late blastula stage [77].

The pattern of maternal gene replacement by zygotic genes remains to be determined for rotifers. Nevertheless, zygotic transcript abundance may commence in REs after gastrulation, which occurred at the 16-cell stage (approximately one h PE in AMs and 2–4 h PE in REs; Fig. 1). To explore the transcript abundance patterns of potential maternal genes, we identified 699 annotated genes (out of 1,087 maternal genes) with differential transcript abundance patterns between AMs and REs over the first two hours of development (1–2 h PE). These putative maternally inherited genes were assigned to ~ 40 KEGG pathways (with at least five allocated genes per pathway, Additional file 2, S2B Table). A previous study of annual-killifish embryos very early in development (at the 1–2-cell stage) identified 57 differentially expressed transcripts between diapause-destined embryos and continuously-developing embryos; many differences between these two types of embryos were gene splice variants and antisense sncRNAs [52]. Of the 57 killifish genes, five genes showed differential transcript abundance between AMs and REs (including the Titin-like, Rho guanine nucleotide exchange factor 12-like, Rho GTPase activating protein 39, Dynein cytoplasmic 1 heavy chain 1, and spermatogenesis associated 6; Additional file 2, S2A Table). The functional significance of these genes in dormancy remains unknown.

Clustering analysis grouped the 1,087 putative maternal gene transcripts into seven clusters (Additional file 2, S4 Table and Additional file 4, S2A Fig); cluster 1 in AM (1–3 h PE) and cluster 3 in RE (1–4 h PE) showed high transcript abundance at the earliest stages of development, supporting the identification of maternal genes in these clusters (Additional file 4, S2A Fig). The spliceosome was the only pathway enriched in cluster 1. Although this pathway was enriched in both AMs and REs during early development (Fig. 3A and B), the transcript abundance of the associated genes was higher in AMs than in REs (cluster 1; Additional file 4, S2B Fig), indicating higher transcriptional activity and formation of mature mRNAs in AMs. The genes in REs clusters 4–7 (6–12 h PE; Additional file 4, S2A Fig) may not be maternal, as the corresponding zygotic counterparts may have replaced many genes. For AMs, the enriched pathways of maternal genes from 3 h PE onwards (clusters 2, 4, and 5), included the ribosome biogenesis in eukaryotes, the Notch signaling pathway, oxidative phosphorylation and fatty acid elongation.

#### The middle phase of development

In contrast to the early stage of embryonic development, where many pathways were enriched in both AMs and REs, most enriched pathways in the later stages of development were distinct between the two types of embryos. In addition, the pathways enriched in the middle stage of development (AM: 4–9 h PE; RE: 6–9 h PE) differed from those enriched during the early and late phases of development. The middle phase of development coincided with organogenesis in AMs and the suspension of development in REs (Additional file 2, S1 Table), as reflected by the higher number of genes transcribed in AMs as compared to REs (Additional file 4, S1 Fig). Transcript abundance patterns overlapped in several gene clusters between the middle and early or middle and late phases of development (AM clusters 2, 3, 4, 5, and 6; RE clusters 2, 3, 4, 5, and 7; Figs. 2B and 3). In several clusters (AM clusters 5 and 6; RE clusters 2, 3, 4, and 5; AM vs. RE clusters 3 and 5), transcripts abundance peaked in the middle phase of development, followed by a decline (Fig. 3). Fourteen pathways were enriched in the middle stage of development in AMs but only ten in REs, as detailed in Additional file 2, S3 Table. Five pathways were unique to REs (Hippo, Wnt, TGFβ, JAK-STAT and the sphingolipid signaling pathways) and one was unique to AMs (the Notch signaling pathway). Several pathways enriched in cluster 2 of REs, were associated with the cell cycle, which was longer in REs, compared with AM. Many pathways associated with energy metabolism were enriched in AM cluster 2, including oxidative phosphorylation, carbon metabolism, the citrate cycle (TCA), pyruvate metabolism, oxidative phosphorylation, and the pentose phosphate pathway (Fig. 3A). In contrast, many of these energy-generating pathways presented lower transcript abundance in REs from the middle phase of development than AMs (cluster 4, Fig. 3C). The biosynthesis of secondary metabolites was also enriched in AMs but not in REs (Additional file 2, S3 Table). Enrichment of the ribosome biogenesis in eukaryotes pathway in AM and AM vs. RE, and the ribosome pathway in AM, RE, and AM vs. RE (Fig. 3A–C), implied relatively high translational activity in the two types of embryos. The axon guidance representing a key stage in the formation of the neuronal network, was enriched in AM (cluster 4), RE (cluster 2), and AM vs. RE (cluster 5). It peaked at the beginning of the middle phase of development and declined afterward (Fig. 3A–C). Most interestingly, the longevity regulating pathway (worm) was enriched in the genes with differential transcription abundance between AMs and REs (cluster 3); this cluster had higher transcript abundance in REs than in AMs (Fig. 3C). Longevity pathway genes were associated with diapause [1, 2] and the long-term survival of plant seeds [61].

#### The late phase of development

The late phase (10–12 h PE in AMs and REs) precedes AMs hatching and many more genes were transcribed in AMs than in REs (Additional file 4, S1 Fig). The transcript abundance in several pathways peaked during this phase (AMs clusters 7 and 8; REs clusters 6, 7, and 9; Fig. 3), and there were notable differences in the transcript abundance patterns between AMs and REs. The differential transcript abundance between AMs and REs was associated with 50 enriched functional pathways, representing a wide array of functions suggesting a role at the termination of embryo development and during hatching (Fig. 3C, cluster 8 and Additional file 2, S3 Table). A total of 15 signaling pathways were enriched in AM clusters 7 and 8 (Additional file 2, S3 Table), including the Notch signaling pathway, which plays a role in development (reviewed in [78]). In contrast, three signaling pathways were enriched in RE cluster 6 but none in cluster 7 and 8. Genes of cluster 6 with higher transcript abundance in the REs as compared with the AMs (Fig. 3C), were enriched with the protein processing in the endoplasmic reticulum, protein export, and the lysosome pathways, suggesting higher synthesis and export of proteins and also protein degradation (Fig. 3C). Higher transcript abundance was found for AM relative to RE at the late time points in Fig. 3C cluster 4. As discussed, cluster 4 was enriched in several energy-generating metabolic pathways, the actin cytoskeleton regulatory pathway, and the Hippo signaling pathway, corresponding with the termination of organogenesis and preparation for AMs hatching.

#### The very late phase of development

AMs hatching typically occurred at ~ 12 h PE, although many AM embryos hatched during 13–14 h PE. In both AMs and REs, many genes displayed differential transcript abundance between the very late phase of development (AMs: 13–14 h PE; REs: 24–192 h PE) and earlier developmental stages (1–12 h PE for both AMs and REs; Additional file 2, S2 and S5 Table).

**Fig. 6:**
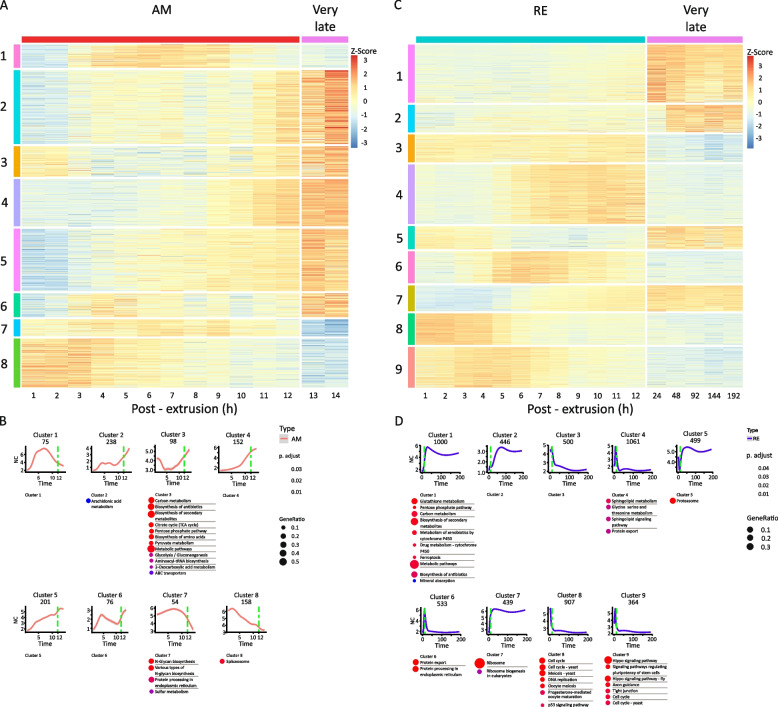
Comparison of the differential transcript abundance profiles during the very late phase of development in AMs and REs. **A** Heatmap of the differential transcript abundance patterns of genes in AMs between the very late phase of development (13–14 h PE) and earlier developmental stages (1–12 h PE), showing the eight identified clusters. **B** The transcript abundance pattern of each cluster and the corresponding enriched pathways. **C** Heatmap of the differential transcript abundance patterns of genes in REs between the very late phase of development (24–192 h PE) and earlier developmental stages (1–12 h PE), showing the nine identified clusters. **D** The transcript abundance pattern of each cluster and the corresponding enriched pathways. In B and D, the colored circles prepending each pathway indicate the statistical significance of the enrichment (color) and the relative proportion of total genes (size)

In the AMs, 1,052 genes (697 genes with annotation) were differentially transcribed between the very late development stage (13–14 h PE) and earlier developmental stages (1–12 h PE; Additional file 2, S2 and S5 Table). The annotated genes were associated with ~ 60 functional KEGG pathways (with at least five genes in each pathway; Additional file 2, S2B Table), demonstrating the vast array of putative functions at this developmental stage. The 1,052 genes formed eight clusters (Fig. 6A and B; Additional file 2, S5A and S5B Table), and in general, transcript abundance patterns at the very late phase in development continued trends observed in the earlier late developmental phase. Enriched pathways associated with genes with high transcript abundance during very late development (e.g., cluster 3) included mainly energy-yielding pathways (Fig. 6B). In contrast, enriched pathways associated with genes with relatively low transcript abundance during very late development (e.g., clusters 7 and 8) included N-glycan biosynthesis, protein processing in the endoplasmic reticulum, sulfur metabolism and the spliceosome. This transcript abundance pattern corresponds with the end of embryo developmental processes and the preparation for hatching.

**Fig. 7:**
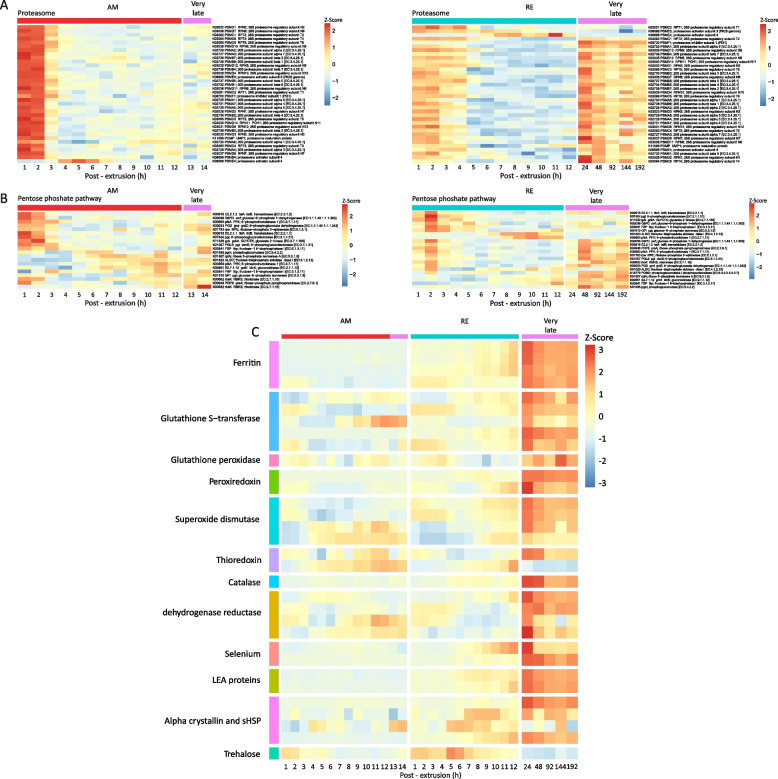
Heatmaps showing the temporal transcript abundance patterns of functionally important genes, very late in the development of AMs and REs as compared to earlier time points (**A**) Genes in the proteasome pathway, (**B**) Genes in the pentose phosphate pathway, (**C**) Genes encoding dormancy hallmark proteins. Additional details of genes encoding dormancy hallmark proteins are given in Additional file 2, S6 Table

In REs, the very late developmental phase corresponded with the period following the formation of the extra-embryonic space, which occurred at ~ 12 h PE [Additional file 2, S1 Table; Additional file 1, S1A Video (RE)]. Remarkably, differential transcript abundance of 5,720 genes was identified between the very late time development (24–192 h PE) and earlier development stages (1–12 h PE), of which 4,062 genes were annotated (Additional file 2, S2 Table). The annotated genes were associated with ~ 200 KEGG pathways (with at least five genes in a pathway; Additional file 2, S2B Table), indicating a wide range of putative functional pathways for this phase. Clustering analysis allocated the genes into nine clusters (Fig. 6C and D, Additional file 2, S5C and S5D Table). Clusters 1, 2, 5, and 7 presented genes with exceptionally high transcript abundance at this phase in comparison with earlier developmental stages (Fig. 6C). Examples of this transcript abundance patterns are shown for the ribosome and the pentose phosphate pathways and of dormancy hallmark proteins (Fig. 7A–C). The high transcript abundance of genes during this phase suggests an essential function during dormancy or a role at the exit from dormancy. These transcripts include mRNAs synthesized before or after 24 h, depending on the availability of ATP (see below), and maintained up to 192 h, or later during dormancy. The enriched transcript abundance in cluster 1 (Fig. 6C and D) includes gene transcripts of the pentose phosphate pathway; most of the pentose phosphates derived from this pathway are incorporated into DNA in rapidly dividing cells. Moreover, the pentose phosphate pathway is a major source of NADPH which is consumed during fatty acid synthesis. The NADPH is also required for the generation of reduced glutathione (GSH), a major scavenger of reactive oxygen species (ROS; reviewed in [79] and these functions are of importance during dormancy and at the exit from it. Glutathione metabolism was also enriched in this cluster. This pathway plays a prominent role in several critical cell functions, ranging from antioxidant defense to metabolic pathway regulation, and includes two dormancy hallmark proteins [76]. Another enriched pathway was ferroptosis with the synthesis of ferritin. Ferritin, another dormancy hallmark protein, is one of the most abundant proteins in REs [76]. In clusters 5 and 7, the proteasome and ribosome pathways were enriched, respectively. The proteasome is presumably implicated in the degradation of dormancy-specific proteins, and the ribosome pathways might function in translation activity, at the exit from dormancy.

Pathways enriched in the genes with relatively low transcript abundance at the very late phase in RE development were identified in RE clusters 3, 4, 6, 8, and 9 (Fig. 6D). Notably, these genes showed relatively high transcript abundance in AMs and REs at 1–3 h and 1–6 h PE, respectively, and described above for the early developmental phase (a complete list of the enriched pathways is given in Additional file 2, S5 Table). While the low transcripts abundance at the very late RE development emphasizes the suspension of the cell cycle and its associated pathways, it is expected that these pathways will be renewed during the exit from dormancy.

Genes encoding for dormancy hallmark proteins showed high transcript abundance during this phase (Fig. 7C, Additional file 3, S2 Text; Additional file 2, S6 Table), consistent with the high abundance of these proteins in mature REs and suggesting that these proteins were synthesized after 12 h PE or during this phase of development [76]. The dormancy hallmark proteins include the late embryogenesis abundant proteins (LEAs), which enhance desiccation tolerance and serve as protein chaperones during dormancy [80] and references therein; reviewed in [33]). Several proteins participate in macromolecule stabilization and ameliorating ROS-associated damage, including ferritin, glutathione S-transferase, glutathione peroxidase, superoxide dismutase, catalase, thioredoxin, dehydrogenase reductase, selenium binding proteins, α-crystallin, and small heat shock proteins (sHSP) (Fig. 7C) [33]. In addition, two proteins (superoxide dismutase and catalase) also play an essential role in the dormancy-associated longevity regulation pathway [1, 2]. An examination of genes associated with the longevity pathway revealed 98 annotated genes, 31 with high transcript abundance (> 4.591) at 192 h PE (Additional file 2, S6 Table; Additional file 4, S3 Fig).

At the latest developmental time point assessed in REs (192 h PE), high transcript abundance was observed for 3,062 genes (> 4.591), of which 1,803 showed significant differences in transcript abundance between earlier developmental stages (1–12 h PE) and very late development phase (24–192 h PE). See the list in Additional file 2, S2E and S2B Table, for the corresponding enriched KEGG pathways. In general, higher transcript abundance of specific genes may stem from reduced gene expression. Although mRNAs per embryo were reduced about 3–4 fold at 24–192 h (data not shown), the fold-change in transcript abundance was much higher (> log 4.591, Additional file 2, S2F Table), suggesting de novo transcription after 12 h.

**Fig. 8:**
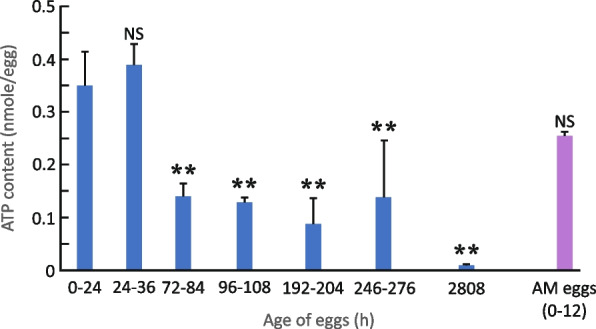
ATP levels (Mean ± SD) during the development of REs and the dormant stage (1–2808 h) in comparison with AMs (1–12 h PE). ATP levels in REs decreased significantly from 36 h PE (*n* = 3 for each time point)

Of the 3,062 genes with high transcript abundance, 34 were identified as encoding dormancy hallmark proteins as described above (Additional file 2, S6 Table; Fig. 7C). In addition, other highly abundant gene transcripts (> 4.591 at 192 h PE) suggest preparation for the renewal of embryo development at the exit from dormancy. These include genes that participate in energy generation pathways (Additional file 4, S4 Fig), signaling pathways (Additional file 4, S5 Fig), lipid metabolism (Additional file 4, S6 Fig) and the light response (phototransduction, circadian entrainment, and the circadian rhythm (Additional file 4, S7 Fig). Notably, although many genes in energy-forming pathways show high transcript abundance very late in RE development, ATP levels were significantly reduced from 72 h PE compared to earlier RE developmental stages (0–36 h; one-way ANOVA, *p* = 0.001; Tukey post-hoc test, *p* < 0.01; Fig. 8). RE ATP levels remained low through the end of the assessment period (280 h post-hoc) and were similarly low in REs stored for ~ 117 days or 2808 h). Consistent with this, previous studies have indicated that a slow reduction in metabolic rate is one of the hallmarks of dormancy. In *Artemia franciscana* dormant embryos, the ATP level drops significantly during diapause. However, substantial quantities of ATP remain and the concentration of ATP in diapause embryos is about fivefold lower compared with post-diapause embryo. Moreover, no evidence of an ongoing metabolism was reported during bouts of anoxia [1, 81]. ATP was not detected in previous metabolomic analyses [82] and very low levels were detected at 2808 h (Fig. 8), possibly because of anoxia. Significant differences were also reported between AMs and dormant REs for the relative abundances of metabolites associated with the following pathways: glycolysis, the citrate cycle, amino acid metabolism, purine metabolism, and pyrimidine metabolism [82]. Notably, ATP levels did not differ significantly between AMs and REs earlier in development (AMs: 0–14 h PE; REs: 0–24 h PE; one-way ANOVA, *p* = 0.2513; Tukey post-hoc test, *p* = 0.24176).

The results presented here suggest that the very late phase of RE development serves as a unique period of molecular preparation for dormancy. In some ways, this preparatory phase resembles the maturation and late maturation phases of plant seed development, characterized by the improvement of functional traits such as germination capacity and desiccation tolerance, as and the acquisition of dormancy and longevity traits. Like REs, the induction of sHSP and the accumulation of LEA proteins occurs during the late maturation of plant seeds [61]. However, unlike plant seeds, RE development does not end with desiccation, but REs acquire this capacity [59]. In dried plant seeds, the mRNAs transcribed during maturation are stored and protected for long-term storage associated with monosomes. They are translationally upregulated during germination [83, 84]. Because REs also store mRNAs [76], it is tempting to conclude that REs and plant seeds use similar dormancy strategies.

### Pathways with a putative role in RE dormancy

We explored transcript abundance profiles of pathways that may play a role in dormancy and its regulation. These include signaling pathways, genes encoding transcription factors, homeobox genes, nuclear receptors, genes associated with lipid droplets, and the light response.

#### Signaling pathways

Signaling pathways transduce extracellular signals to regulate target gene transcription of and facilitate cell specifications differentiating from progenitor cells during embryogenesis [85]. To explore their role in RE dormancy, we focused on genes in signaling pathways previously identified as dormancy-associated in other organisms: Wnt, Notch, TGFβ, Hippo, Hedgehog, AKT, JAK-STAT, MAPK, AMPK, insulin signaling, FoxO, and mTOR (see Introduction; Additional file 3, S2 Text). Of the genes with differential transcript abundance between AMs and REs (dataset AM vs. RE; Additional file 2, S7 Table), 394 were associated with these 12 signaling pathways. This number includes 130 signaling-associated genes with high transcript abundance (> 4.591) in REs during the entry into dormancy (192 h PE; Additional file 2, S7 Table). Essential membrane receptors, such as Frizzled, IGFIR, BMPR1, BMPR2, TGFBR1, EGFR, RTK, Notch, and HH (Additional file 2, S7 Table; Additional file 4, S5 Fig) were among the signaling-associated genes with differential transcript abundance between AMs and REs. Membrane receptors transduce external cell-surface signals and translocate components of the intracellular cascade (e.g. second messenger molecules and kinases) into the nucleus, activating or repressing transcription [85]. Thus, differences in transcript abundance of these membrane receptors suggest significant changes in the activation of gene transcription between AMs and REs.

**Fig. 9:**
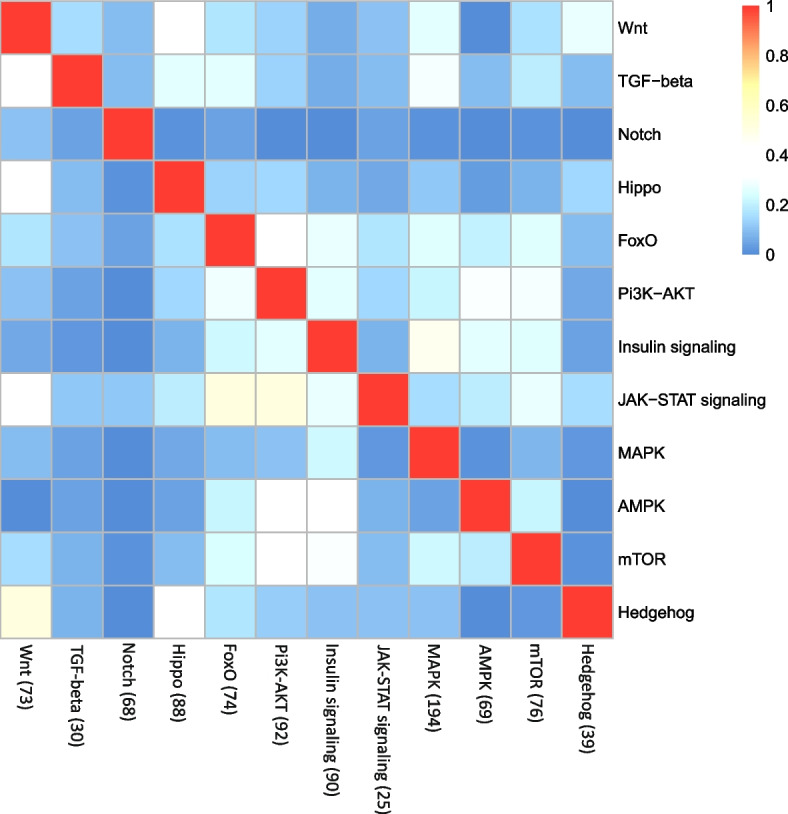
Heatmap showing the numbers of genes shared among 12 signaling pathways in REs. Each cell reflects the fraction of genes shared between the two pathways relative to the total number of genes in the pathway on the x-axis (the total number of genes is shown in brackets). Additional details are shown in Additional file 2, S7 Table

The assignment of the same genes in different signaling pathways suggested functional crosstalk between the pathways (including all 570 annotated genes listed in Additional file 2, S7 Table). Thus, the Wnt pathway shared 51.2% of its genes with the Hedgehog pathway, 36.7% with the TGFβ signaling pathway, 31.8%, with the Hippo pathway, and 17.6% with the FoxO signaling pathway. Similarly, the FoxO pathway shared 28.3% of its genes with the PI13-AKT pathway, 26.7% with the TGFβ pathway, and 22.2% with the Insulin signaling pathway. The insulin signaling pathway shared 34.8% of it genes with the AMPK pathway, 30.2% with mTOR, and 27.0% with FoxO. Moreover, 47.8% of the genes of the MAPK pathway were shared with the Insulin signaling pathway and 25.7% with the FoxO pathway (Fig. 9). Many of the mechanisms underlying cell-type specification rely on interactions among signaling pathways, and many examples of cross-regulation involving multiple different pathways have been reported. Prominent examples include crosstalk between TGFβ/BMP and MAPK, PI3-AKT, Wnt, Hedgehog, and Notch [86] or between TGFβ/Smad and Wnt, Notch, Hippo, Hedgehog, MAPK, PI3-AKT, and JAK-STAT [78]. A crosstalk between TGFβ, the Hedgehog and Notch signaling pathways, was reported for *C. elegans* [40]. Particularly well-known is the association between insulin and FoxO signaling pathways (see Introduction). The negative regulation of the nuclear function of FoxO by the insulin/PI3K/AKT pathway is a hallmark of the FoxO family [87]. Surprisingly, the rotifer FoxO forkhead box transcription factor, which participates in the FoxO, PI3K-AKT, and AMPK signaling pathways (Additional file 4, S5 Fig) and was here annotated as FoxO6/FoxO3 (gene c24194_g1, NCBI RMZ95845.1), with very low transcript abundance throughout RE development (Additional file 2, S7 Table). Rotifer FoxO6 and FoxO3 are similar to the *C. elegans* FoxO (*daf-16*) and *Drosophila* FoxO proteins [87]. It is, therefore, suggested that FoxO was unlikely to play a role in RE dormancy.

Interestingly, in AM most of the signaling pathways were enriched at later stages of development (Fig. 3A), while in RE clusters signaling pathways were enriched early in development (< 6 h PE; Fig. 3B). In addition, a high transcript abundance of the signaling pathways genes were identified in RE at 192 h (Additional file 2, S5 Fig). Therefore, signaling pathways may play an essential role in AM embryo development before hatching and at the exit from dormancy in REs by mRNAs stored during the dormant state.

#### Transcription factors (TFs)

The primary consequence of signaling is the activation of specific target genes by signal-regulated TFs [88]. TF transcript abundance patterns reflect the DNA binding activity of these regulatory proteins, which control the transcriptional changes that occur during metazoan embryonic development [89]. To explore the role of TFs in RE development, we first identified 65 genes encoding for six classes of TFs (bHLH, bZIP, ETS, Fox, HMG, and T-box) in AMs and REs (Additional file 2, S8 Table). Of these, 45 genes exhibited differential temporal transcript abundance patterns between AMs and REs. As functions of TFs in rotifers are poorly investigated (excluding homeobox genes, as discussed below), it remains to be seen whether TF functions described during embryogenesis in other organisms are conserved in rotifers. It is noteworthy that CLOCK, a member of the bHLH transcription gene family with a central role in the regulation of circadian rhythms, showed high transcript abundance in both AMs (> 5.247) and REs (> 4.591), during development (see further discussion below).

#### Homeobox genes

Homeobox genes encode for TFs that bind DNA via the homeodomain consensus motif to regulate the expression of target genes in numerous developmental processes. Despite their seemingly similar DNA-binding properties, homeobox proteins display specific effects on the transcriptome, mediated through variations of amino acids in their DNA-binding motifs. Moreover, a significant fraction of downstream genes encode for realizator functions, which directly affect morphogenetic processes, such as orientation and rate of cell divisions, cell–cell adhesion and communication, cell shape and migration, cell differentiation and cell death [90].

**Fig. 10:**
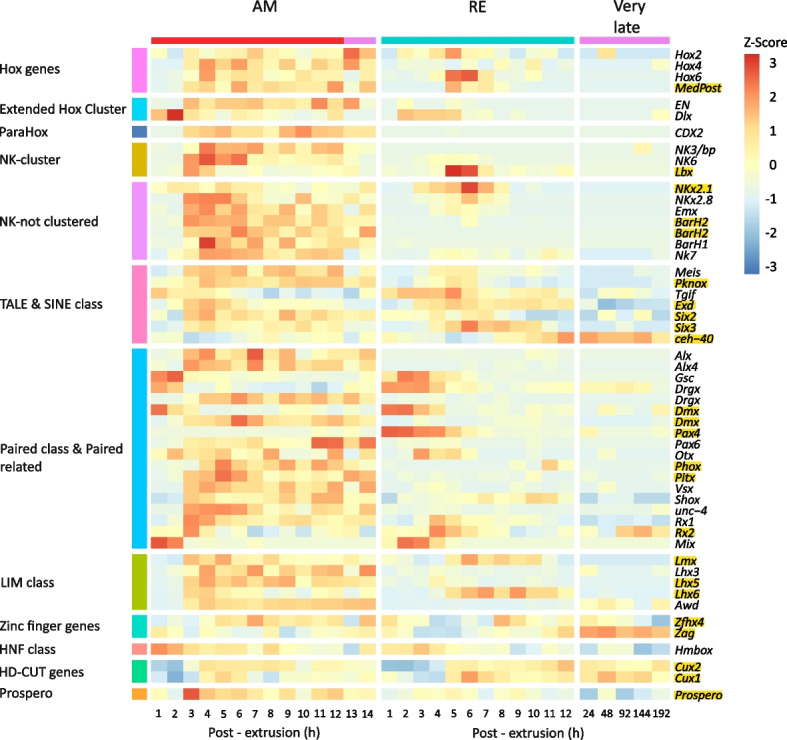
Heatmaps showing the temporal transcript abundance profiles of homeobox genes during AM and RE development. Genes within each homeobox family with significantly different temporal transcript abundance profiles between AMs and REs (during 1–12 PE h) are highlighted in yellow. Further details of all genes are given in Additional file 2, S9 Table

Eleven homeobox gene families are usually recognized: ANTP, PRD, LIM, POU, HNF, SINE, TALE, CUT, PROS, ZF, and CERS [91, 92]. We identified 53 homeobox genes in the REs and AMs transcriptomes (Additional file 2, S9 Table, Fig. 10). Relatively low transcript abundance was identified for fourteen homeobox genes (< 1.69). Throughout AMs and REs development (0–12 h PE), a differential pattern in transcript abundance was identified of 45 and 25 homeobox genes, respectively, and the transcript abundance patterns of 23 homeobox genes differed between AMs and REs (Additional file 2, S9 Table). In AMs, low initial transcript abundance of 34 genes was observed at 1–2 h PE, (< 1.87), but they increased as development progressed (Fig. 10). This transcript abundance pattern was consistent with previous observations in other species (i.e., *Xenopus tropicalis*, *Danio rerio*, *Ciona intestinalis*, *Drosophila melanogaster*, *C. elegans*, and *Anopheles gambiae*), where homeobox TF expression was under-represented among the maternal transcripts and increased significantly after the onset of gastrulation [89]. Non-canonical expression patterns were reported for five Hox genes (*Hox2*, *Hox4*, *Hox3*, *Hox6,* and *Medpost*) in the AM nervous system during embryogenesis [93]. Variable temporal transcript abundance patterns were found for these genes in AM and RE during embryonic development (Additional file 2, S9 Table). Although transcripts for *Hox3* and *Hox5* were identified in the reference transcriptome, these genes were not detected in the CEL-seq analysis, probably due to low transcript abundance. The low transcript abundance of these *Hox* genes is possibly based on the expression of these genes in only a few cells. Many TFs genes were identified downstream as *Hox* targets, often being activated at differing time points [89].

Many genes with an association to differentiation and imprinting of the nervous system showed moderate or high transcript abundance during AMs and REs development (AMs: 1–14 h PE; REs: 1–192 h PE), including *DRGX* (Paired class and Paired related class), *ZAG* (a Zinc finger gene), *HMBOX* (an HNF class gene), *CUX1* and *CUX2* (HD-CUT class genes), *Prospero* (a Prospero class gene), *TGIF* and *ceh-40* (TALE & SINE class), and *RX2* (Paired class and Paired related class). In addition, *AWD* (LIM class), with a function in the development of trachea and imaginal discs in insects, also presented high transcript abundance (Fig. 10).

Interestingly, several homeobox genes associated with the visual system, including *RX1*, *VSX*, *SIX3*, *OTX*, *PITX* and *Pax6*, displayed low transcript abundance (< 1.69) during the very late stage of RE development [94]. Remarkably, the transcript abundance of *Pax6*, which is known to be associated with rotifer eye development [95], was higher (> 1.69) in AM from 3 h onwards, in concordance with the development of a functional eye at hatching (Fig. 10). This was surprising, as illumination is required for RE hatching [56, 60] and calls into question the target receptors that respond to light in REs (further below).

#### Nuclear receptors

Nuclear receptors are ancient TFs that are activated by metazoan ligands regulating key metabolic and developmental pathways [96]. In *C. elegans*, the nuclear receptor *daf-12* regulates diapause and longevity [4]. As we identified a gene annotated as *daf-12* in the CEL-seq transcriptome, we explored the transcript abundance patterns of nuclear receptors during the embryonic development of AMs and REs (Additional file 3, S2 Text). Of the 29 nuclear receptor genes previously identified in the *B. plicatilis* genome [97], the transcripts of 25 were identified in both AMs and REs (Additional file 2, S10 Table). A few of these mRNA sequences were annotated as more than one gene, suggesting difficulties in the identification. In addition, several nuclear receptor gene transcripts (annotated as E78C, RXRA, RXRG, FTZ-F1β, HUNKA, and Daf-41) were highly abundant very late in RE development (192 h PE; > 4.591), suggesting a functional role at the exit from dormancy.

Two groups of nuclear receptors have been associated with dormancy: those linked to ecdysteroids and those involved in the dauer formation in *C. elegans* [98]. The maternal contribution of ecdysteroids is well-known in insects including *Drosophila* [99]. The nuclear receptors associated with ecdysteroids include the ecdysone receptor EcR (NRH1; an ortholog of the farnesoid X receptor, FXR, or the liver X receptor, LXR), which binds 20E to form the 20E-EcR-USP complex together with Ultraspiracle (USP; an ortholog of the vertebrate RXR receptor, [99]). Several nuclear receptors are the main targets of the 20E-EcR-USP complex. They are regulated by 20E, including DHR3, HR4, HR39, E75, E78, and *ftz*- factor 1 (FTZ-F1), and transcripts of these genes were identified in AMs and REs (Additional file 2, S10 Table), suggesting a function for ecdysone in AMs and REs. An association between low ecdysone levels and embryonic diapause was found in the diapause-type eggs of the silkworm *Bombyx mori* [100]. In rotifers, exposure to 20E increased mictic female production, but the effects of 20E on REs are unclear [101], and more studies are required to determine the role of ecdysteroids in the embryonic development of AMs and REs.

Four genes in the CEL-seq transcriptomes were annotated as *C. elegans* orthologs; *daf-12*, *daf-36*, *daf-41*, and *nhr-23. daf-12* was previously annotated as the 20E-induced insect ortholog H96 [97]. *daf-12* is homologous to the vertebrate farnesoid-X (FXR), liver-X, and Vitamin D (VD) receptors, which are known to regulate a wide range of functions. *daf-12* is regulated by bile acid-like steroids called dafachonic acids (DAs), which bind to *daf-12* to prevent dauer formation. The first step in DA biosynthesis (the transformation of cholesterol into 7-dehydrocholesterol) involves Daf-36/Rieske oxygenase. 7-desaturation, catalyzed by the orthologous *neverland*/Rieske oxygenase, is the first step in insect ecdysteroid biosynthesis [4]. In vertebrates, 7-dehydrocholesterol is converted to VD3 and the exposure of annual killifish embryos to VD3 analogs and Δ4-DA stimulates continuous development despite diapause-inducing conditions [37]. Thus, it is tempting to suggest that *daf-36* may also participate in dormancy regulation in rotifers, especially as its transcripts abundance in AMs are significantly higher than of REs (Additional file 2, S10 Table). In preliminary experiments, the exogenous addition of 7-dehydrocholesterol (at concentrations ranging from 0.1 nM to 10 μM) to the culture medium neither affected the number of REs produced, nor induced the hatching of formed REs (i.e., the exit from dormancy) during the obligatory dormant period (data not shown). The function of *daf-36* in rotifer dormancy has not been determined. Transcripts of *daf-41* were highly abundant in AMs (> 5.247) and REs (> 4.591; Additional file 2, S10 Table), but their function in rotifers has not been investigated. In *C. elegans daf-41,* a co-chaperone of *hsp90/daf-21* participates in the regulation of longevity, larval entry and exit from the dauer stage, and responses to environmental cues such as oxidative stress (https://www.uniprot.org/uniprotkb/Q23280). The *C. elegans* nuclear receptor *nhr-23* is an ortholog of *Drosophila* DHR3 (discussed above), and both *nhr-23* and DHR3 participate in molting [4]. The function of *nhr-23* in rotifers remains unclear, as rotifers (in contrast to *C. elegans* and insects) do not molt.

### Lipid droplets

Rotifer REs are characterized by the presence of LDs, which are formed during RE development [55, 102]. Under starvation conditions, the survival of hatched RE neonates containing LDs was higher than hatched AM neonates [103]. Lipid accumulation characterizes the preparatory diapause phase in many animals and used during diapause (e.g. the dauer stage and insects [1]), The accumulated lipids are stored as lipid droplets (LDs) [104–108]. LDs are evolutionarily conserved cellular organelles with a unique ultrastructure, consisting of a neutral lipid core containing triacylglycerols (TAG) and sterol esters, and encircled by a phospholipid monolayer that is studded with integral and peripheral proteins. LDs play roles in lipid biosynthesis and hydrolysis and are tightly coupled to cellular metabolism and energy homeostasis [106].

In total, 26 proteins associated with LDs were identified in the AM and RE transcriptomes (Additional file 2, S11 Table). Transcripts of genes encoding for the LD-associated proteins tended to be highly abundant (> 4.591) late in RE development. The transcript abundance patterns of 21 of the 26 LD-associated genes differed significantly between AMs and REs, suggesting the formation of LDs for lipid storage during dormancy. Further transcriptomic exploration of the lipid metabolism in AMs and REs identified several genes associated with fatty acid biosynthesis, elongation, unsaturated fatty acid biosynthesis, and fatty acid degradation. Significant differences were found between AMs and REs (AM vs. RE) in the abundance for most of the gene transcripts of the four KEGG maps (left panels in Additional file 4, S6A–S6D Figs), implying a difference between AMs and REs in the fatty acids stored in LDs. Moreover, most of the gene transcripts were highly abundant (> 4.591) at 192 h, suggesting a role during dormancy or at its exit (right panels in Additional file 4, S6A–S6D Figs). Surprisingly, enzymes synthesizing long-chain unsaturated fatty acids (e.g., docosahexaenoic acid and eicosapentaenoic acid), were transcribed in the embryo transcriptome (Additional file 2, S2A Table). A low transcript abundance was found at 192 h in REs for carnitine O-palmitoyltransferase 2 (CPT2, K08766), located in the inner mitochondrial membrane and a key enzyme in fatty acid degradation, implying an attenuated degradation of fatty acids during dormancy. As LDs were also observed in amictic females in *B. koreanus* [109], we propose that the conspicuous appearance of LDs in REs might be linked with a lower turnover in REs as compared with AMs.

### The light response

REs hatch after an obligatory dormant period in response to illumination [56, 60]. Hence, it is reasonable to assume that the light response is intrinsic in the formation of dormant REs. Hatching rates of REs were highest in response to UV radiation at 350–400 nm, but the eyespots of adult rotifers absorb light in the range of 470–540 nm [56, 110]. The dormant stages of several crustacean species hatch in response to light, including *Artemia*, *Daphnia pulex*, and *Tripos granaries* [111]. In these crustaceans, the light response precedes eyespot formation, and each species may possess multiple visual pigments with distinct spectral classes. However, the elicited intracellular response cascades remain unknown. In REs, the transcripts abundance of the homeobox *Pax6* gene and the PNR nuclear receptor suggest the absence of a functional photoreceptor.

To unveil functional pathways associated with the response to light, we searched the reference transcriptome and Additional file 2, S2A Table for transcripts of the phototransduction, circadian rhythm, and circadian entrainment pathways (Additional file 2, S12 Table). Twelve putative opsin genes were identified in the *B. koreanus* genome [110], but only eight genes annotated as opsins were identified in the AMs and REs transcriptomes (Additional file 2, S13 Table). The transcript abundance patterns of seven opsins differed between AMs and REs and only three (PDE6D, OPSD1, and OPS1) showed relatively high transcript abundance at 192 h PE. Whether these opsins function at the exit from dormancy remains to be shown. The transcript abundance patterns of six or seven genes (out of 12 or 13) were identified in the phototransduction pathway (Additional file 4, S7A Fig). Almost all (excluding one) exhibited differential abundance between REs and AMs, and most showed low transcript abundance in REs (Additional file 2, S12A Table; Additional file 4, S7A Fig, left panel). Only two genes with high transcript abundance were identified at 192 h (the right panel in Additional file 4, S7A Fig). As some key genes were not identified in this pathway, it remains to be determined whether this pathway represents phototransduction in rotifers. The higher sensitivity of REs to UV irradiation may be associated with the lower abundance of L-kynurenine compared to AMs). L-kynurenine is an ultraviolet chromophore that in vertebrates prevents light below 400 nm from reaching the retina (discussed in [82]).

The transcript abundance patterns of many genes in the circadian rhythm and circadian entrainment pathways differed between AMs and REs. Most of these differentially abundant gene transcripts (Additional file 4, S7B and S7C Figs) exhibited very high abundance at 192 h in the REs, including *PER*, *CLOCK*, *CRY*, and *REV-ERBA* of the circadian rhythm. Interestingly, the transcript abundance patterns of most of the membrane receptors of the circadian entrainment pathway differed between AMs and REs. Moreover, most of the genes in these KEGG pathways were identified in the AMs and REs transcriptomes. It remains, however, to be determined whether the genes of these pathways respond to external stimuli or participate in the regulation of the obligatory dormancy period.

## Conclusions

**Fig. 11:**
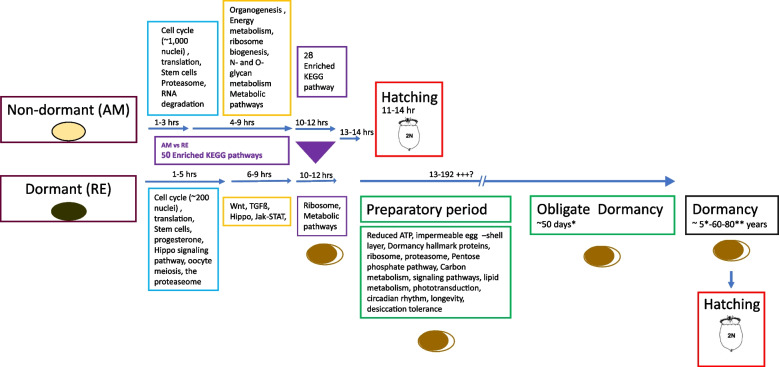
A simplified overview of the differences in embryonic developmental trajectories between AMs and REs, from egg extrusion until hatching. [*12, **13]

Our results highlighted several prominent similarities and differences in the functional development trajectories of dormant (REs) and non-dormant embryos (AMs) (Fig. 11). The trajectory was divided into four phases, with the first being the most similar between AMs and REs. The significant divergence between AMs and REs starts with the second phase and increases in the third phase, which ends embryonic development in AMs. Finally, a preparatory phase for dormancy characterizes the fourth phase in REs. It is reminiscent of the maturation phase, preparing “orthodox” plant seeds for the long dormant period.

The cell cycle pathway occurred only during the early developmental phase in REs and AMs, but it was slower in RE, and fewer nuclei (~ 200) were observed than in AMs (~ 1,000) before it stopped. Strikingly, in both REs and AMs, the translation of nuclear mRNA presumably relied entirely on maternal ribosomal proteins. The Hippo pathway, in association with other enriched signaling pathways, may function in the cessation of nuclei cleavage. Degradation of maternal proteins and mRNAs presumably occurred at this phase.

In AMs, in contrast to REs, the middle phase of development (4–9 h PE) was characterized by the enrichment and higher abundance of genes associated with the metabolic and energy-yielding pathways. Many homeobox genes displayed relatively high transcript abundance patterns during this phase in AMs, concomitant with organogenesis, while their transcript abundance patterns were low in REs. The enrichment of numerous signaling pathways characterized the middle stage of RE development.

Significant differences in enriched pathways distinguished the late stage of development (10–12 h PE for both AMs and REs); genes in ~ 50 functional pathways, including 15 signaling pathways, disclosed differential transcript abundance patterns between AMs and REs during this period. This remarkable difference emphasized the physiological distinction between the preparation for hatching in AMs and the preparation for dormancy in REs.

The very late stage of RE development (24–192 h PE) affords a unique and novel insight into the molecular preparations for long-term dormancy. Reduced ATP levels marked this phase, in addition to differences in the abundance of more than 5,000 gene transcripts, compared with earlier time points (1–12 h PE), and the very high transcript abundance of ~ 3,000 genes. In general, higher transcript abundance of specific genes may stem from reduced gene expression. Although mRNAs per embryo were reduced at 24–192 h, the fold-change in transcript abundance was much higher, suggesting de novo transcription after 12 h. The list of very highly abundant genes includes genes encoding for dormancy hallmark proteins, proteins participating in macromolecular proteins and proteins involved in antioxidant protection, longevity, and desiccation tolerance, presumably synthesized before or during this phase and stored in REs. Many functionally essential pathways were enriched with highly abundant gene transcripts during the very late developmental period, including the ribosome, the pentose phosphate, energy-yielding metabolism, signaling, the proteasome, and lipid metabolism pathways. Although the preparatory dormancy period appeared to continue past the 192 h of observation, the length of this period remains unknown. REs are refractory to hatching signals and appear to develop the light response and desiccation tolerance several weeks after formation [59]. Following the initial preparatory period, REs retain viability for several years or even decades (e.g., 80 years, [13]; 65–100 years, [12]).

As to the signaling pathways, most were enriched in REs at the early and middle developmental phases, while in AMs, most were enriched at the late phase of development. Moreover, crosstalk among signaling pathways was suggested by shared genes, implying that changes in the transcript abundance pattern of a few genes could contribute to enhancing or reducing the functions of numerous signaling pathways. In contrast to many organisms displaying short-term dormancy or diapause, the low transcript abundance of FoxO at all developmental stages of RE suggest that it may not be involved in regulating of dormancy. Surprisingly, REs and AMs express nuclear receptors associated with the regulation of diapause in insects and *C. elegans*.

Lipid metabolism plays a crucial role in the survival of *C. elegans* in the dauer stage, and diapausing insects and possibly also in REs, while metabolism is reduced, but not entirely abolished. REs contain a prominent number of LDs; the transcript abundance profiles of genes encoding for LD proteins differed between AMs and REs. Most genes in the fatty acid biosynthesis, fatty acid elongation, and biosynthesis of unsaturated fatty acids presented differential abundance of gene transcripts between AM and REs, suggesting differences in the type of lipids stored in REs. Remarkably, the low transcript abundance of a key enzyme in fatty acid degradation (CPT2) in REs at 192 h PE implies that fatty acids were not metabolized during dormancy.

Light induces hatching, however, a low transcript abundance of genes associated with the visual system suggests a lack of a functional eyespot in REs, and the photoreceptor remains unknown. Interestingly, almost all of the genes currently reported in the circadian rhythm and circadian entrainment KEGG pathway maps were identified in the REs and AMs. While the transcript abundance patterns of many of these genes differed between the two egg types, their role in eliciting a response to external stimuli and regulating the obligatory dormant period remains unknown.

One of the most striking findings of our study was the apparent similarity between the late developmental stage in REs and the late maturation stage of "orthodox" plant seeds, which desiccate and can survive for centuries [19, 20]. A comparison of REs and plant seeds in Table 1 revealed several convergent functional solutions appearing during late development and late maturation in REs and plant seeds, respectively. These functional convergences suggest that REs behave like animal “seeds.”

**Table 1:** Long-term dormancy: a functional comparison between plant seeds and resting eggs

Plant seeds	Resting eggs (RE)
Survive for centuries in a desiccated form [20]	Survive for decades in a wet form [12, 13]
Mature or immature embryos [112]	Development is suspended after gastrulation (this study)
Mechanical barriers; cutin, suberin, pectin, β glucans [113]	Impermeable eggshell layer [17, 55]
Low metabolism resulting from desiccation [61]	Low ATP levels without desiccation (this study)
Late maturation phase [61, 114]	Dormancy preparatory period (this study)
1. Acquire tolerance to desiccation and dehydration [61, 114]	1. Remain wet but acquire tolerance to desiccation [59]
2. Expression of sHSPs, LEA, anti-ROS respiratory enzymes, and longevity [61]	2. High expression of sHSPs, LEA, anti-ROS, dormancy hallmark proteins ribosomes, pentose phosphate pathway, longevity, and signaling pathways ( [76], this study)
3. Storage of ribosomes, mRNAs as monosomes, stress granules, and P-body proteins [83]	3. Store mRNAs [76]
4. Storage of starch/ lipids, sugars, disaccharides [114]	4. Storage of glycogens and lipids (trehalose?) [55, 82]
After-ripening: post-maturation stage acquiring the capacity to germinate [114]	Refractory to external stimuli during the obligate dormancy period while acquiring the capacity to tolerate desiccation [59]
Germination after water imbibition [112]	Responsive to light for exiting dormancy [56, 60]
Translation during germination of stored mRNAs [83]	?

This study provides novel insights into the molecular mechanisms underlying the entry into long-term embryonic dormancy, which characterizes organisms that produce dormant egg banks in response to variable environmental conditions. This work markedly expands our understanding of dormancy and highlights the gaps in our knowledge for future investigations.

## Materials and methods

### Rotifer culture methods

Rotifers (clone ATB4; [59]) were cultured in artificial seawater (ASW; salts were purchased from Red Sea, Israel) at 24–26°C and fed *Nannochloropsis* sp. (Galil Algae, Israel). Synchronous entrance into sexual reproduction and RE production was achieved by transferring rotifers from a high salinity environment (40 ppt; 42.5 g L^−1^) to a low salinity environment (10 ppt; 10.75 gL^−1^). Additional details of the cultural conditions are given below.

### Experimental design

The experiment aimed to identify genes functionally associated with long-term dormancy by comparative transcriptome profiling of transcript abundance patterns during the development of REs and AMs. First, because *B. plicatilis* forms a large taxonomic complex, a novel transcriptome assembly was constructed using the full-length RNA sequences from *B. plicatilis* at various life stages (see Additional file 3, S1 Text). The workflow of the experiment was as follows: sample collection at one h intervals during the first 12 h of development and at late time points (13–14 h PE in AM and 24, 48, 92, 144, and 192 h PE in RE), mRNA extraction, single embryo CEL-seq mRNA sequencing, CEL-seq transcriptome assembly, and bioinformatics analysis of the RNA-seq data. The novel *B. plicatilis* transcriptome was a reference for annotating the RNA CEL-seq 3'-end specific sequences. Because the onset of dormancy in most organisms involves the cessation of the cell cycle and metabolic depression, the number of nuclei and the ATP content were determined for AMs and REs throughout development. These measurements were later associated with the transcriptomic data. In parallel with sampling, time-lapse microscopic imaging was performed to monitor morphological changes from the time of egg extrusion to hatching for AMs and 48 h PE for REs.

### Sample collection

Fertilized females that had not yet extruded REs were identified based on a black dot within the abdomen and randomly selected from two 600 ml cultures (6–8 days old); each culture contained 15,000–20,000 rotifers at ten ppt ASW. At this point, both cultures contained morphologically distinct rotifers: amictic females, unfertilized mictic females carrying male eggs, males, fertilized mictic females carrying REs and females without eggs (for a description of the rotifer life cycle, refer to [17]). Each randomly selected fertilized female was placed in one well of a 96-well flat-bottom plate with 100 μl of culture medium (10 ppt ASW containing ~ 1.6 × 10^6^ cells/ml of *Nannochloropsis* sp.); 4–6 plates (~ 400–600 individual females) were prepared. Plates were kept at 24–26 °C and inspected every hour. At each inspection, females with an extruded RE were collected and transferred to a new culture plate with 100 μl of culture medium for each female in a well. The RE was detached from the female and retained but the female was discarded. REs were collected hourly from 0–12 h PE (5–7 eggs per time point) and then at 24, 48, 72, 92, 144 and 192 h, as RE formation may last up to five days [56].

Non-dormant AM eggs were collected similarly from 2-day-old 10 ppt cultures (600 ml, 10 ppt ASW), as the cultures contained almost no mictic females. Young females that had not yet extruded an egg were placed in 96-well plates (one female per well) as described above for REs. Also, as described above plates were inspected hourly, and females carrying extruded eggs were transferred to a new plate; the egg was retained, and the female was discarded. AMs were collected hourly from 0–14 h after extrusion (5–7 eggs per time point). Most AMs hatched at ~ 12 h; unhatched AMs were collected at 13–14 h. Each collected RE or AM was washed twice in sterile filtered phosphate-buffered saline (PBS; Sigma-Aldrich), inserted into a marked 1.5 ml Eppendorf tube, and flash-frozen in liquid nitrogen and stored at − 80 °C.

The viability of eggs subjected to the procedure described above was tested. Over 90% of the AMs hatched despite the separation from the maternal females and repeated washing. After 3–8 months (following the obligatory dormancy period of 6–8 weeks, [59]), the REs hatching rate was 30–40%, like that of untreated REs that were collected from the same culture (data not shown) and as reported in [59].

### Morphological and physiological analyses

#### Documentation of AM and RE development using time-lapse photography

Time-lapse microscopic imaging was used to monitor the development of RE and AM embryos [Additional file 1, Video S1A (RE), S1B (AM)]. AMs were imaged for 12–14 h, from the egg’s detachment, until the neonates’ hatching, while REs were imaged for 48 h after detachment. Images were taken at time intervals of one minute. We chose to focus on the 48 h after RE extrusion because, under our culture conditions (ten ppt ASW at 23–25°C), REs typically detach from the maternal female at this time. Five full replicate films (of 10 trials) were obtained of AMs from different cultures and on different days, spanning the period from ~ 30 min after extrusion to hatching time. Similarly, five full replicate films (of 11 trials) were obtained of REs from different cultures and on different days. Live microscopic images were captured using an inverted microscope (DMIRE2, Leica or Cell Observer, Zeiss) equipped with an AxioCam HS camera (LS&E Microscopy Core Facility, Technion-Israel Institute of Technology, Haifa, Israel).

#### Staining of nuclei

REs and AMs were obtained as described above. However, different sampling time points were used: AMs were collected at 0, 1–2, 2, 4, 6, 8, 10, 12, and 13 h after extrusion, while REs were collected at 0, 2, 4, 6, 8, 10, 12, 13, 13–15, 24, 48, 72, 124, 144 h, ~ 1.5 months, and ~ 4.5 months after extrusion. After collection, the collected REs and AMs were stained with Hoechst 33342 nuclear staining dye (Sigma); REs older than 15 h were decapsulated before staining [62]. Images of these REs and AMs were captured by using an inverted widefield fluorescent microscope (DMi8, Leica) with a 20 × objective lens (NA 0.75) to capture bright-field images of unstained eggs and fluorescent images of nuclear-stained eggs. Deconvolution software (Leica LASX) was applied to improve the image contrast and was used to determine the number of nuclei and measure their size.

#### Determination of ATP content

AMs and REs were obtained and sampled as described above. At each time point, 50 REs or 50 AMs were transferred into a sterile 1.5 ml Eppendorf vial and homogenized in 0.1 ml PBS buffer (Sigma). The homogenate was heated to 100°C for 10 min and cooled on ice. The vials were centrifuged (Eppendorf Centrifuge; 12,000 G at 4°C for 20 min), and each supernatant was transferred to a new vial, flash-frozen in liquid nitrogen, and stored at − 80°C until analysis. Once all of the time-course samples had been collected, ATP content was determined for all frozen samples simultaneously. The frozen samples were thawed on ice and centrifuged at 12,000 g for 20 min at 4°C (Eppendorf Centrifuge). The supernatant of each sample was filtered using Corning Costar Spin-X sterile centrifuge tube filters with cellulose acetate membranes (pore size 0.22 μm; Sigma). The ATP content was measured in 70 μl of each filtered sample, with an ab113849 Luminescent ATP Detection Assay Kit (Abcam), following the manufacturer's instructions and using white flat-bottom 96-well plates (Catalogue number CLS3917**,** Merck). Luminescence was measured using a Clario-Star plate-reading spectrophotometer (BMG). ATP was measured in three replicate sets of time-course RE samples from three different cultures; the AMs samples were collected from three cultures and were considered the positive control. Statistical analysis of the results was performed using Statistica version 14.0.0.15 (TIBCO Software Inc.). One-way ANOVAs and Tukey Post Hoc tests were performed after confirming that the data were normally distributed.

### Molecular analyses

#### *B. plicatilis* reference transcriptome construction

The *B. plicatilis* reference transcriptome was assembled from pooled RNA samples of a variety of life stages (details in Additional file 3, S1 Text) using Trinity (https://github.com/trinityrnaseq/trinityrnaseq/wiki). The datasets generated during the current study are available NCBI, Accession PRJNA821380 ID 821380 at https://www.ncbi.nlm.nih.gov/bioproject/PRJNA821380. To reach the list of samples click on 171 of SRA experiments in the Project Data table. After reaching the web page, click on "Send results to Run selector" and the reached webpage contains a list of the samples. Samples 1–155 (SRR18532591-SRR18532745) and 156–171 (SRR215415774-SRR21541589) correspond with the Cel-seq and the transcriptome libraries, respectively. Reference transcriptome assembly yielded 50,628 transcripts and 35,242 putative genes. The longest isoform of each gene was annotated using Blast2GO (https://www.blast2go.com) InterProScan (https://www.ebi.ac.uk/interpro/search/sequence), and the KEGG Automatic Annotation Server (KAAS) (https://www.genome.jp/kegg/kaas). Further details of reference transcriptome assembly and annotation are given in Additional file 3, S1 Text.

Single-embryo RNA extraction and CEL-seq: REs and AMs embryos were collected and sampled as described above. RNA was extracted from 5–7 individuals of REs or AMs at each time point using Trizol reagent (Thermo-Fischer Scientific), following the manufacturer's instructions. RNA from 155 samples was sequenced and analyzed, consisting of five replicates from each time point of AM (1–14 h PE) and RE (1–192 h PE). After quality control of count data, 19 samples were removed due to both the small library size and the low number of transcribed genes. Moreover, 20,463 genes were removed from the analysis due to a very low average count. In total 136 samples with 14,779 genes remained for downstream analysis.

Library construction and 3'-end CEL-seq were performed as previously described [115], yielding an average of 2.5 M raw reads per sample. The CEL-seq transcriptome is available at NCBI (accession no. PRJNA821380, samples SRR18532591-SRR18532745). Further details of CEL-Seq are given in Additional file 3, S1 Text.

#### Transcript abundance profiling

To identify differences in transcript abundance profiles between AMs and REs over the course of development, the 3'-end CEL-seq reads from the single embryo samples were aligned to the reference transcriptome (available at NCBI accession no. PRJNA821380, samples SRR21541574-SRR21541589, using Bowtie2 (https://bowtie-bio.sourceforge.net/bowtie2/index.shtml, v.2.2.6) and relative transcript abundance were estimated using RSEM (https://github.com/deweylab/RSEM, v1.2.25). Low-quality count data were filtered out using the R scater package (https://bioconductor.org/packages/release/bioc/html/scater.html). Counts were normalized using variance stabilizing transformation in DESeq2 (https://bioconductor.org/packages/release/bioc/html/DESeq2.html). Transcript abundance is in concordance with mRNA gene expression level.

"Low transcript abundance" was defined as a value less than one standard deviation above the global minimum (i.e., the mean value for REs of < 1.69 and < 1.87 for AMs; Additional file 2, S2A Table), whereas "high transcript abundance" was defined as a value of at least two standard deviations greater than the global minimum (i.e., > 4.591 for REs and > 5.247 for AMs). Values between these two extremes were considered “moderate transcript abundance”. Further quality control and normalization details are given in Additional file 3, S1 Text.

Four sets of analyses were carried out (Additional file 2, S2A Table): 1) Identification of genes that have changed significantly during development (1–12 h PE) of each egg type, AMs and REs. 2) Identification of genes with differential transcript abundance patterns between AMs and REs (AM vs. RE) during development (1–12 h PE). 3) Identification of genes that show differences in transcript abundance between AMs and REs in very early time points (1–2 PE), which were considered as the maternal effect time points. 4) Identification of differential abundance of gene transcripts between the early stages of development (1–12 h PE) and the very late stages of development (> 12 h PE) in both egg types. Further details of the statistical analyses and models used to identify genes displaying differential transcript abundance are given in Additional file 3, S1 Text. In addition, hierarchical clustering and functional enrichment analyses were performed as described in Additional file 3, S1 Text.

### Exploration of the genes with differential transcript abundance patterns during AMs and REs development using KEGG pathways and transcript abundance profiling

To explore functional differences between AMs and REs, we selected specific functionally relevant genes from the normalized gene transcripts dataset (listed in Additional file 2, S2A Table): genes encoding for transcription factors, homeobox genes, nuclear receptors, genes associated with signaling pathways, lipid droplets, lipid metabolism, phototransduction, circadian rhythm, and circadian entrainment. We constructed transcript abundance profiles of genes and/or KEGG maps for these functionally relevant genes to explore critical differences in AMs and REs development. Detailed descriptions of the pathways and genes analyzed are given in Additional file 3, S2 Text, Additional file 2, S6–S13 Tables, and Additional file 4, S2–S7 Figs. Descriptions of KEGG reference pathways (https://www.genome.jp/pathway/) can be obtained by applying the name of the pathway and “Reference pathway”. Descriptions of genes or protein function is available at uniport (https://www.uniprot.org/).

We also explored the temporal transcript abundance patterns during the development of genes encoding for the dormancy hallmark proteins. The list of dormancy hallmark proteins was obtained from [76]. To identify corresponding genes in Additional file 2, S2A Table, the nucleotide sequences of contigs genes from the transcriptome of Ziv et al. [76], were used in a BLASTX search for the corresponding contigs in the reference transcriptome in the current study.

### Supplementary Information

**Additional file 1: S1 Video.** Time-lapse photography during the development of dormant (A, RE) and non-dormant (B, AM) embryos.

**Additional file 2: Tables S1.** Embryonic developmental stages in AMs and REs, based on observations of time-lapse photographs. **S2 Table.** The temporal mean normalized gene counts (DeSeq2-VSD log-scale normalization) during the development of AMs (1–14 h post-extrusion) and REs (1–192 h post-extrusion). **S3 Table.** Cluster analyses (mean normalized transcript abundance values). **S4 Table.** (A) Cluster analysis of maternal gene transcripts (1-2 h after extrusion) with differential abundance between AMs and REs, and (B) the corresponding enriched KEGG pathways. **S5 Table.** (A, C) Cluster analyses of transcripts with differential abundance between early development (1–12 h post-extrusion) and very late development in each egg type. **S6 Table.** (A)The temporal transcript abundance patterns of transcripts encoding for dormancy hallmark proteins during the very late in RE development (24–192 h post extrusion).  (B) The temporal transcript abundance patterns of genes in the KEGG longevity regulating pathway (worm) in AMs (1–14 h post-extrusion) and REs (1–192 h post-extrusion). **S7 Table.** (A) The temporal transcript abundance patterns of genes associated with the following signaling pathways: Wnt, TGFβ, Notch, Hippo, FoxO, PI3-AKT, Insulin signaling, JAK-STAT, MAPK, AMPK, mTOR, and Hedgehog. **S8 Table.** The temporal transcript abundance patterns of genes encoding for transcription factors in AMs and REs. **S9 Table.** The temporal transcript abundance patterns of homeobox genes in AMs and REs. **S10 Table.** The temporal transcript abundance patterns of genes encoding for nuclear receptors in AMs and REs. **S11 Table.** The temporal transcript abundance patterns of (A) genes associated with lipid droplets and genes involved in KEGG lipid metabolic pathways. **S12 Table.** The temporal transcript abundance patterns of genes involved in the (A) phototransduction, (B) circadian rhythm, and (C) circadian entrainment KEGG pathways in AMs and REs. **S13 Table.** List of opsin-associated genes in AMs and REs.

**Additional file 3: Text. S1.** Bioinformatics. **S2 Text.** Transcript abundance profiles [116, 117].

**Additional file 4:** **Figure. S1.** Number of reads sequenced per sample across three REs and AMs developmental stages. **S2 Fig.** Comparison of the transcript abundance profiles of putative maternal genes between AMs and REs. **S3 Fig.** The Longevity pathway (worm) highlights differential transcript abundance between AM and RE (left panel) and highly abundant protein-encoding genes at 192 h in RE (right panel). **S4 Fig.** Very highly abundant gene transcripts (>4.251) of energy-yielding KEGG pathways. (>4.251) at 192 hr of RE. **S5 Fig.** KEGG signaling pathways (maps) highlighting protein-encoding genes with differential transcript abundance between AM and RE (left panel) and highly abundant transcripts at 192 hr in RE (right panel). **S6 Fig.** Lipid metabolism KEGG pathways (maps) highlighting protein-encoding genes with differential transcript abundance between AM and RE (left panels) and highly abundant transcripts at 192 hr in RE (right panel). **S7 Fig.** Light responding KEGG pathways (maps) highlighting protein-encoding genes with differential transcript abundance between AM and RE (left panels) and transcripts with high abundance at 192 hr in RE (right panels).

## Data Availability

Data are available in the main text or the supplementary materials. In addition, the *B. plicatilis* transcriptome and Cel-seq data are available at NCBI, Accession PRJNA821380 ID 821380 at https://www.ncbi.nlm.nih.gov/bioproject/PRJNA821380. To reach the list of samples click on 171 of SRA experiments in the Project Data table. After reaching the web page, click on "Send results to Run selector" and the reached webpage contains a list of the samples. Samples 1–155 (SRR18532591-SRR18532745) and 156–171 (SRR215415774-SRR21541589) correspond with the Cel-seq and the transcriptome libraries, respectively. Any additional information can be obtained from elubzens@gmail.com (one of the corresponding authors).

## Abbreviations

AMs: Diploid amictic eggs
Ar-SETD4: Histone lysine methyltransferase from *Artemia*
CPT2: Fatty acid degradation
Das: Dafachonic acids
EcR: Ecdysone receptor
FTZ-F1: *ftz*-Factor 1
FXR: Vertebrate farnesoid-X
GO: Gene ontology
GSH: Generation of reduced glutathione
H4K20me3: Trimethylated histone H4K20
IGF: Insulin growth factor
IIS: Signaling
KO: KEGG Orthology
LEAs: Late embryogenesis abundant proteins
MBT: Mid-blastula transition
NHs: Trajectories of dormant
ROS: Reactive oxygen species
sHSP: Small heat shock proteins; single embryo RNA sequencing
TAG: Triacylglycerols
TCA: Citrate cycle
TFs: Transcription factors
VD: Vitamin D
VDR: Vitamin D receptor

## References

[1] Hand SC, Denlinger DL, Podrabsky JE, Roy R (2016). Mechanisms of animal diapause: recent developments from nematodes, crustaceans, insects, and fish. Am J Physiol Regul Integr Comp Physiol.

[2] Karp X. Hormonal regulation of diapause and development in nematodes, insects, and fishes. Front Ecol Evol. 2021;9. 10.3389/fevo.2021.735924.

[3] Denlinger D (2022). Insect diapause.

[4] Antebi A. Nuclear receptor signal transduction in *C. elegans*. WormBook. 2015:1–49. 10.1895/wormbook.1.64.2. 10.1895/wormbook.1.64.2 PMC5402207 26069085

[5] Koštál V (2006). Eco-physiological phases of insect diapause. J Insect Physiol.

[6] Baumgartner MF, Tarrant AM (2017). The physiology and ecology of diapause in marine copepods. Ann Rev Mar Sci.

[7] Wourms JP (1972). The developmental biology of annual fishes. III. Pre-embryonic and embryonic diapause of variable duration in the eggs of annual fishes. J Exp Zool.

[8] van der Weijden VA, Bulut-Karslioglu A. Molecular regulation of paused pluripotency in early mammalian embryos and stem cells. Front Cell Dev Biol. 2021;9. 10.3389/fcell.2021.708318. 10.3389/fcell.2021.708318 PMC8353277 34386497

[9] Storey KB, Storey JM (2012). Aestivation: signaling and hypometabolism. J Exp Biol.

[10] Carvalho GR, Wolf HG (1989). Resting eggs of lake- Daphnia I. Distribution, abundance and hatching of eggs collected from various depths in lake sediments. Freshw Biol.

[11] Hairston NG, Van Brunt RA, Kearns CM, Engstrom DR (1995). Age and survivorship of diapausing eggs in a sediment egg bank. Ecology.

[12] Kotani T, Ozaki M, Matsuoka K, Snell TW, Hagiwara A, Sanoamuang L, Segers H, Shiel RJ, Gulati RD (2001). Reproductive isolation among geographically and temporally isolated marine Brachionus strains. Rotifera IX Developments in hydrobiology Vol 153.

[13] García-Roger EM, Carmona MJ, Serra M (2006). Patterns in rotifer diapausing egg banks: Density and viability. J Exp Mar Biol Ecol.

[14] Radzikowski J (2013). Resistance of dormant stages of planktonic invertebrates to adverse environmental conditions. J Plankton Res.

[15] Frisch D, Morton PK, Chowdhury PR, Culver BW, Colbourne JK, Weider LJ (2014). A millennial-scale chronicle of evolutionary responses to cultural eutrophication in *Daphnia*. Ecol Lett.

[16] Alekseev VR, Stasio BT, Gilbert JJ, Dumont HJ (2007). Diapause in aquatic invertebrates: Theory and human use. Monographiae biologicae.

[17] García-Roger EM, Lubzens E, Fontaneto D, Serra M (2019). Facing adversity: Dormant embryos in rotifers. Biol Bull.

[18] Hansen BW (2019). Copepod embryonic dormancy: “An egg is not just an egg”. Biol Bull.

[19] Shen-Miller J, Mudgett MB, Schopf JW, Clarke S, Berger R (1995). Exceptional seed longevity and robust growth: ancient Sacred Lotus from China. Am J Bot.

[20] Sallon S, Solowey E, Cohen Y, Korchinsky R, Egli M, Woodhatch I (2008). Germination, genetics, and growth of an ancient date seed. Science.

[21] Keilin D (1959). The problem of anabiosis or latent life: history and current concept. Proc R Soc Lond B Biol Sci.

[22] Clegg JS (2001). Cryptobiosis—a peculiar state of biological organization. Comp Biochem Physiol B, Biochem Mol Biol.

[23] Fontaneto D, De Smet WH, Schmidt-Rhaesa A (2015). Handbook of zoology, gastrotricha, cycloneuralia and gnathifera. Handbook of zoology, gastrotricha, cycloneuralia and gnathifera Volume 3, gastrotricha and gnathifera. Vol. 3, gastrotricha and gnathifera.

[24] Clegg JS, Conte FP, Persoone G, Sorgeloos P, Roels O, Jaspers E (1980). A review of the cellular and developmental biology of Artemia. The brine shrimp artemia, physiology, biochemistry, molecular biology.

[25] Boschetti C, Leasi F, Ricci C (2011). Developmental stages in diapausing eggs: an investigation across monogonont rotifer species. Hydrobiologia.

[26] Chen L, Barnett RE, Horstmann M, Bamberger V, Heberle L, Krebs N (2018). Mitotic activity patterns and cytoskeletal changes throughout the progression of diapause developmental program in Daphnia. BMC Cell Biol.

[27] MacRae TH (2016). Stress tolerance during diapause and quiescence of the brine shrimp, Artemia. Cell Stress Chaperones.

[28] Rowarth NM, MacRae TH. ArHsp40 and ArHsp40–2 contribute to stress tolerance and longevity in Artemia franciscana, but only ArHsp40 influences diapause entry. J Exp Biol. 2018;221(Pt 20). 10.1242/jeb.189001. 10.1242/jeb.189001 30158133

[29] Tan J, MacRae TH (2019). The synthesis of diapause-specific molecular chaperones in embryos of Artemia franciscana is determined by the quantity and location of heat shock factor 1 (Hsf1). Cell Stress Chaperones.

[30] Li A-Q, Sun Z-P, Liu X, Yang J-S, Jin F, Zhu L (2019). The chloride channel cystic fibrosis transmembrane conductance regulator (CFTR) controls cellular quiescence by hyperpolarizing the cell membrane during diapause in the crustacean Artemia. J Biol Chem.

[31] Malitan HS, Cohen AM, MacRae TH (2019). Knockdown of the small heat-shock protein p26 by RNA interference modifies the diapause proteome of *Artemia franciscana*. Biochem Cell Biol.

[32] Gbotsyo YA, Rowarth NM, Weir LK, MacRae TH (2020). Short-term cold stress and heat shock proteins in the crustacean Artemia franciscana. Cell Stress Chaperones.

[33] Lubzens E, Cerda J, Clark M (2010). Dormancy and resistance in harsh environments. Topics in current genetics. Vol. 21Topics in current genetics.

[34] Reynolds JA (2019). Noncoding RNA regulation of dormant states in evolutionarily diverse animals. Biol Bull.

[35] Ragland GJ, Denlinger DL, Hahn DA (2010). Mechanisms of suspended animation are revealed by transcript profiling of diapause in the flesh fly. Proc Natl Acad Sci.

[36] Ragland GJ, Keep E (2017). Comparative transcriptomics support evolutionary convergence of diapause responses across nsecta. Physiol Entomol.

[37] Romney ALT, Davis EM, Corona MM, Wagner JT, Podrabsky JE (2018). Temperature-dependent vitamin D signaling regulates developmental trajectory associated with diapause in an annual killifish. Proc Natl Acad Sci.

[38] Kučerová L, Kubrak OI, Bengtsson JM, Strnad H, Nylin S, Theopold U (2016). Slowed aging during reproductive dormancy is reflected in genome-wide transcriptome changes in Drosophila melanogaster. BMC Genom.

[39] Koštál V, Štětina T, Poupardin R, Korbelová J, Bruce AW (2017). Conceptual framework of the eco-physiological phases of insect diapause development justified by transcriptomic profiling. Proc Natl Acad Sci.

[40] Liu T, Zimmerman KK, Patterson GI (2004). . Regulation of signaling genes by TGFβ during entry into dauer diapause in C. *elegans*. BMC Dev Biol.

[41] Narbonne P, Roy R (2009). Caenorhabditis elegans dauers need LKB1/AMPK to ration lipid reserves and ensure long-term survival. Nature.

[42] Li H-Y, Lin X-W, Geng S-L, Xu W-H (2018). TGF-β and BMP signals regulate insect diapause through Smad1-POU-TFAM pathway. Biochim Biophys Acta Mol Cell Res.

[43] Bulut-Karslioglu A, Biechele S, Jin H, Macrae TA, Hejna M, Gertsenstein M (2016). Inhibition of mTOR induces a paused pluripotent state. Nature.

[44] Hussein AM, Wang Y, Mathieu J, Margaretha L, Song C, Jones DC (2020). Metabolic control over mTOR-dependent diapause-like state. Dev Cell.

[45] Dai L, Ye S, Li H-W, Chen D-F, Wang H-L, Jia S-N (2017). SETD4 regulates cell quiescence and catalyzes the trimethylation of H4K20 during diapause formation in *Artemia*. Mol Cell Biol.

[46] Zhao L-L, Jin F, Ye X, Zhu L, Yang J-S, Yang W-J (2015). Expression profiles of miRNAs and involvement of *miR-100* and *miR-34* in regulation of cell cycle arrest in *Artemia*. Biochem J.

[47] Kalinka AT, Varga KM, Gerrard DT, Preibisch S, Corcoran DL, Jarrells J (2010). Gene expression divergence recapitulates the developmental hourglass model. Nature.

[48] Diez-Roux G, Banfi S, Sultan M, Geffers L, Anand S, Rozado D (2011). A high-resolution anatomical atlas of the transcriptome in the mouse embryo. PLoS Biol.

[49] Levin M, Anavy L, Cole AG, Winter E, Mostov N, Khair S (2016). The mid-developmental transition and the evolution of animal body plans. Nature.

[50] Yang KY, Chen Y, Zhang Z, Ng PK-S, Zhou WJ, Zhang Y, et al. Transcriptome analysis of different developmental stages of amphioxus reveals dynamic changes of distinct classes of genes during development. Sci Rep. 2016;6(1). 10.1038/srep23195. 10.1038/srep23195 PMC4793263 26979494

[51] Lefebvre F, Lécuyer É (2018). Flying the RNA nest: Drosophila reveals novel insights into the transcriptome dynamics of early development. J Dev Biol.

[52] Romney AL, Podrabsky JE (2017). Transcriptomic analysis of maternally provisioned cues for phenotypic plasticity in the annual killifish, Austrofundulus limnaeus. EvoDevo.

[53] Romney ALT, Podrabsky JE (2018). Small noncoding RNA profiles along alternative developmental trajectories in an annual killifish. Sci Rep.

[54] Birky CW, Gilbert JJ (1971). Parthenogenesis in rotifers: The control of sexual and asexual reproduction. Am Zool.

[55] Wurdak ES, Gilbert JJ, Jagels R (1978). Fine structure of the resting eggs of the rotifers brachionus calyciflorus and asplanchna sieboldi. Trans Am Micros Soc.

[56] Hagiwara A, Hoshi N, Kawahara F, Tominaga K, Hirayama K (1995). Resting eggs of the marine rotifer Brachionus plicatilis Mller: development, and effect of irradiation on hatching. Hydrobiologia.

[57] Martínez-Ruiz C, García-Roger EM (2015). Being first increases the probability of long diapause in rotifer resting eggs. Hydrobiologia.

[58] Gilbert JJ (2017). Resting-egg hatching and early population development in rotifers: a review and a hypothesis for differences between shallow and deep waters. Hydrobiologia.

[59] Lubzens E, Hamo R, Blais I, Jeries S, Almog-Gabai O, Assaraf YG (2020). Rotifer resting eggs: An alternative for rotifer mass cultures in fish farms—Lessons from a comprehensive study on production, storage and hatching of resting eggs. Aquaculture.

[60] Minkoff G, Lubzens E, Kahan D (1983). Environmental factors affecting hatching of rotifer (Brachionus plicatilis) resting eggs. Hydrobiologia.

[61] Leprince O, Pellizzaro A, Berriri S, Buitink J (2017). Late seed maturation: drying without dying. J Exp Bot.

[62] Snell TW, Shearer TL, Smith HA (2011). Exposure to dsRNA Elicits RNA interference in *Brachionus manjavacas* (Rotifera). Mar Biotechnol.

[63] Clément P, Wurdak E, Harrison FW, Ruppert EE (1991). Rotifera. Microscopic anatomy of invertebrates.

[64] Farrell JA, O'Farrell PH (2014). From egg to gastrula: How the cell cycle is remodeled during the *Drosophila* mid-blastula transition. Annu Rev Genet.

[65] Schier AF (2007). The maternal-zygotic transition: Death and birth of RNAs. Science.

[66] Tadros W, Lipshitz HD (2009). The maternal-to-zygotic transition: a play in two acts. Development.

[67] Marlow FL (2010). Developmental biology. Maternal control of development in vertebrates: My mother made me do it!.

[68] Langley AR, Smith JC, Stemple DL, Harvey SA (2014). New insights into the maternal to zygotic transition. Development.

[69] Vastenhouw NL, Cao WX, Lipshitz HD. The maternal-to-zygotic transition revisited. Development. 2019;146(11). 10.1242/dev.161471. 10.1242/dev.161471 31189646

[70] Lee MT, Bonneau AR, Giraldez AJ (2014). Zygotic genome activation during the maternal-to-zygotic transition. Annu Rev Cell Dev Biol.

[71] Stout EP, La Clair JJ, Snell TW, Shearer TL, Kubanek J (2010). Conservation of progesterone hormone function in invertebrate reproduction. Proc Natl Acad Sci.

[72] Snell TW, DesRosiers NJD (2008). Effect of progesterone on sexual reproduction of Brachionus manjavacas (Rotifera). J Exp Mar Biol Ecol.

[73] Yu F-X, Guan K-L (2013). The Hippo pathway: regulators and regulations. Genes Dev.

[74] Macaulay A, Scantland S, Robert C, Grabowski P (2011). RNA processing during early embryogenesis: Managing storage, utilisation and destruction. RNA processing.

[75] Greber BJ, Ban N (2016). Structure and function of the mitochondrial ribosome. Annu Rev Biochem.

[76] Ziv T, Chalifa-Caspi V, Denekamp N, Plaschkes I, Kierszniowska S, Blais I (2017). Dormancy in embryos: Insight from hydrated encysted embryos of an aquatic invertebrate. Mol Cell Proteomics.

[77] Emerson CP, Humphreys T (1971). Ribosomal RNA synthesis and the multiple, atypical nucleoli in cleaving embryos. Science.

[78] Luo K (2017). Signaling cross talk between TGF-β/Smad and other signaling pathways. Cold Spring Harb Perspect Biol.

[79] Patra KC, Hay N (2014). The pentose phosphate pathway and cancer. Trends Biochem Sci.

[80] Hibshman JD, Goldstein B (2021). LEA motifs promote desiccation tolerance in vivo. BMC Biol.

[81] Patil YN, Marden B, Brand MD, Hand SC (2013). Metabolic downregulation and inhibition of carbohydrate catabolism during diapause in embryos of *Artemia franciscana*. Physiol Biochem Zool.

[82] Rozema E, Kierszniowska S, Almog-Gabai O, Wilson EG, Choi YH, Verpoorte R (2019). Metabolomics reveals novel insight on dormancy of aquatic invertebrate encysted embryos. Sci Rep.

[83] Bai B, van der Horst S, Cordewener JHG, America TAHP, Hanson J, Bentsink L (2020). Seed-stored mRNAs that are specifically associated to monosomes are translationally regulated during germination. Plant Physiol.

[84] Sano N, Rajjou L, North HM (2020). Lost in translation: Physiological roles of stored mrnas in seed germination. Plants.

[85] Babonis LS, Martindale MQ (2017). Phylogenetic evidence for the modular evolution of metazoan signalling pathways. Philos Trans R Soc Lond, B, Biol Sci.

[86] Guo X, Wang X-F (2008). Signaling cross-talk between TGF-β/BMP and other pathways. Cell Res.

[87] Schmitt-Ney M (2020). The FOXO’s advantages of being a family: Considerations on function and evolution. Cells.

[88] Perrimon N, Pitsouli C, Shilo BZ (2012). Signaling mechanisms controlling cell fate and embryonic patterning. Cold Spring Harb Perspect Biol.

[89] Schep AN, Adryan B (2013). A comparative analysis of transcription factor expression during metazoan embryonic development. PLoS One.

[90] Carnesecchi J, Pinto PB, Lohmann I (2018). Hox transcription factors: an overview of multi-step regulators of gene expression. Int J Dev Biol.

[91] Holland PWH (2012). Evolution of homeobox genes. Wiley Interdiscip Rev Dev Biol.

[92] Ferrier DEK (2016). Evolution of homeobox gene clusters in animals: The giga-cluster and primary vs. Secondary clustering. Front Ecol Evol.

[93] Fröbius AC, Funch P (2017). Rotiferan Hox genes give new insights into the evolution of metazoan bodyplans. Nat Commun.

[94] Buono L, Martinez-Morales JR (2020). Retina development in vertebrates: Systems biology approaches to understanding genetic programs: Retina development in vertebrates: systems biology approaches to understanding genetic programs. BioEssays.

[95] Boell LA, Bucher G (2008). Whole-mount in situ hybridization in the Rotifer Brachionus plicatilis representing a basal branch of lophotrochozoans. Dev Genes Evol.

[96] Holzer G, Markov GV, Laudet V. Evolution of nuclear receptors and ligand signaling. Curr Top Dev Biol. 2017:1–38. 10.1016/bs.ctdb.2017.02.003. 10.1016/bs.ctdb.2017.02.003 28527568

[97] Kim D-H, Kim H-S, Hwang D-S, Kim H-J, Hagiwara A, Lee J-S (2017). Genome-wide identification of nuclear receptor (NR) genes and the evolutionary significance of the NR1O subfamily in the monogonont rotifer Brachionus spp. Gen Comp Endocrinol.

[98] Gissendanner CR, Crossgrove K, Kraus KA, Maina CV, Sluder AE (2004). Expression and function of conserved nuclear receptor genes in Caenorhabditis elegans. Dev Biol.

[99] King-Jones K, Thummel CS (2005). Nuclear receptors—a perspective from Drosophila. Nat Rev Genet.

[100] Makka T, Seino A, Tomita S, Fujiwara H, Sonobe H (2002). A possible role of 20-hydroxyecdysone in embryonic development of the silkworm *Bombyx mori*. Arch Insect Biochem Physiol.

[101] Gallardo WG, Hagiwara A, Tomita Y, Soyano K, Snell TW, Hagiwara A, Snell TW, Lubzens E, Tamaru CS (1997). Effect of some vertebrate and invertebrate hormones on the population growth, mictic female production, and body size of the marine rotifer Brachionus plicatilis Müller. Live food in aquaculture Developments in hydrobiology Vol 124.

[102] García-Roger EM, Ortells R (2018). Trade-offs in rotifer diapausing egg traits: survival, hatching, and lipid content. Hydrobiologia.

[103] Gilbert JJ (2004). Females from resting eggs and parthenogenetic eggs in the rotifer Brachionus calyciflorus: lipid droplets, starvation resistance and reproduction. Freshw Biol.

[104] Walther TC, Chung J, Farese RV (2017). Lipid droplet biogenesis. Annu Rev Cell Dev Biol.

[105] Pol A, Gross SP, Parton RG (2014). Biogenesis of the multifunctional lipid droplet: Lipids, proteins, and sites. J Cell Biol.

[106] Olzmann JA, Carvalho P (2019). Dynamics and functions of lipid droplets. Nat Rev Mol Cell Biol.

[107] Gao M, Huang X, Song B-L, Yang H (2019). The biogenesis of lipid droplets: Lipids take center stage. Prog Lipid Res.

[108] Kory N, Farese RV, Walther TC (2016). Targeting fat: Mechanisms of protein localization to lipid droplets. Trends Cell Biol.

[109] Lee M-C, Park JC, Yoon D-S, Han J, Kang S, Kamizono S (2018). Aging extension and modifications of lipid metabolism in the monogonont rotifer Brachionus koreanus under chronic caloric restriction. Sci Rep.

[110] Kim H-J, Sawada C, Rhee J-S, Lee J-S, Suga K, Hagiwara A (2014). Nutritional effects on the visual system of the rotifer Brachionus plicatilis sensu stricto (Rotifera: Monogononta). J Exp Mar Biol Ecol.

[111] Kashiyama K, Ito C, Numata H, Goto SG (2010). Spectral sensitivity of light-induced hatching and expression of genes mediating photoreception in eggs of the Asian tadpole shrimp Triops granarius. Comp Biochem Physiol Part A Mol Integr Physiol.

[112] Bewley JD, Nonogaki H. Seed maturation and germination. Reference Module in Life Sciences. 2017:623–626. 10.1016/b978-0-12-809633-8.05092-5.

[113] De Giorgi J, Piskurewicz U, Loubery S, Utz-Pugin A, Bailly C, Mène-Saffrané L (2015). An endosperm-associated cuticle is required for arabidopsis seed viability, dormancy and early control of germination. PLoS Genet.

[114] Angelovici R, Galili G, Fernie AR, Fait A (2010). Seed desiccation: a bridge between maturation and germination. Trends Plant Sci.

[115] Hashimshony T, Wagner F, Sher N, Yanai I (2012). CEL-seq: single-cell RNA-seq by multiplexed linear amplification. Cell Rep.

[116] Kanehisa M, Furumichi M, Sato Y, Kawashima M, Ishiguro-Watanabe M. KEGG for taxonomy-based analysis of pathways and genomes. Nucleic Acids Res. 2023;51(D1):D587–92. 10.1093/nar/gkac963. 10.1093/nar/gkac963 PMC9825424 36300620

[117] Imai KS, Hino K, Yagi K, Satoh N, Satou Y. Transcript abundanceprofiles of transcription factors and signaling molecules in the ascidian embryo: towards a comprehensive understanding of gene networks. Development. 2004;131(16):4047–58. 10.1242/dev.0127. 10.1242/dev.01270 15269171

