# Supplementary material for: A transcriptomic examination of encased rotifer embryos reveals the developmental trajectory leading to long-term dormancy; are they “animal seeds”?
Source: BMC Genomics. 2024 Jan 27;25:119. doi: 10.1186/s12864-024-09961-1 (PMC10821554; doi:10.1186/s12864-024-09961-1)
Supplement: Supplementary file 2 — Additional file 2: Tables S1. Embryonic developmental stages in AMs and REs, based on observations of time-lapse photographs. S2 Table. The temporal mean normalized gene counts (DeSeq2-VSD log-scale normalization) during the development of AMs (1–14 h post-extrusion) and REs (1–192 h post-extrusion). S3 Table. Cluster analyses (mean normalized transcript abundance values). S4 Table. (A) Cluster analysis of maternal gene transcripts (1-2 h after extrusion) with differential abundance between AMs and REs, and (B) the corresponding enriched KEGG pathways. S5 Table. (A, C) Cluster analyses of transcripts with differential abundance between early development (1–12 h post-extrusion) and very late development in each egg type. S6 Table. (A)The temporal transcript abundance patterns of transcripts encoding for dormancy hallmark proteins during the very late in RE development (24–192 h post extrusion). (B) The temporal transcript abundance patterns of genes in the KEGG longevity regulating pathway (worm) in AMs (1–14 h post-extrusion) and REs (1–192 h post-extrusion). S7 Table. (A) The temporal transcript abundance patterns of genes associated with the following signaling pathways: Wnt, TGFβ, Notch, Hippo, FoxO, PI3-AKT, Insulin signaling, JAK-STAT, MAPK, AMPK, mTOR, and Hedgehog. S8 Table. The temporal transcript abundance patterns of genes encoding for transcription factors in AMs and REs. S9 Table. The temporal transcript abundance patterns of homeobox genes in AMs and REs. S10 Table. The temporal transcript abundance patterns of genes encoding for nuclear receptors in AMs and REs. S11 Table. The temporal transcript abundance patterns of (A) genes associated with lipid droplets and genes involved in KEGG lipid metabolic pathways. S12 Table. The temporal transcript abundance patterns of genes involved in the (A) phototransduction, (B) circadian rhythm, and (C) circadian entrainment KEGG pathways in AMs and REs. S13 Table. List of opsin-associated genes in AMs and REs. [file 12864_2024_9961_MOESM2_ESM.zip › Additional File 2 Table legends_ESM.docx]

**Additional File 2: Table Legends**

**S1 Table.**doc**, Embryonic developmental stages in AMs and REs, based on observations of time-lapse photographs.**

**S2 Table.** xlxs, (**A**) **The temporal mean normalized gene counts (DeSeq2-VSD log-scale normalization) during the development of AMs (1–14 h post-extrusion) and REs (1–192 h post-extrusion).** Transcripts with significant differences of abundance in the following six analyses (details in Additional Files 3, S1 Text) are presented: throughout the development of AMs (1-12 h post-extrusion; A_pval, column AO)) or REs (1-12 h post-extrusion; RE_pval; column AP); transcripts with differential abundance between AMs and REs during 1-12 h post-extrusion (AM_vs. RE_pval, column AQ); between AMs and REs over 1-2 h post-extrusion (Maternal_pval, column AR); between early development (1-12 h post-extrusion) and very late development for each egg type, AMs and REs (AMs: 13-14 h post-extrusion, column AS. REs: 24-192 h post extrusion, column AT). All p-values were corrected for multiple testing. (**B**) KEGG pathways and the corresponding number of genes with each pathway for KOs of all transcripts and the KOs of the six data sets listed above. We also identified the KEGG pathways associated with KOs corresponding with genes highly expressed late in AM, with values >5.247 at 14 h and a matching list displaying the number of genes that differed between Early vs. Very Late AM at this time point. Similarly, KEGG pathways with KOs corresponding with genes with high transcript abundance in the development of RE with values >4.591 at 192 h and a matching list displaying the number of genes that differed between Early vs. Very Late RE at this time point. (**C**) List of AM genes with values >5.247 at 14 h post-extrusion. (**D**) List of AM genes with KO numbers and values >5.247 at 14 h post-extrusion. (**E**) List of RE genes with values >4.591 at 192 h post-extrusion. (**F**) List of the RE genes with KO numbers and values >4.591 at 192 h post-extrusion. Pathways related to diseases, vertebrate physiology, or plant physiology were removed from this dataset.

**S3 Table.** xlxs,**Cluster analyses (mean normalized transcript abundance values).**(**A**) during AM development (1–14 h post-extrusion), (**B**) during RE development (1–192 h post-extrusion), and (**C**) between AMs and REs (AM vs. RE; 1-12 h post-extrusion). The enriched KEGG pathways corresponding to each cluster with differential transcript abundance are shown in sheets D, E, and F, respectively. A link (URL) to the corresponding KEGG map for each enriched pathway is given in column L of sheets D, E, and F. Enriched pathways related to diseases, vertebrate physiology, or plant physiology were removed from all datasets. Data shown in sheets A, B, and C were extracted from Additional File 2, S2A Table (see also Figs 2 and 3 in the main text).

**S4 Table.** xlxs, (**A**) **Cluster analysis of maternal gene transcripts (1-2 h after extrusion) with differential abundance between AMs and REs, and (B) the corresponding enriched KEGG pathways.** A link (URL) to the corresponding KEGG map for each enriched pathway is given in column L of sheet B. Enriched pathways related to diseases, vertebrate physiology, or plant physiology were removed from this dataset. Data shown in sheet A were extracted from Additional File 2, S2A Table (see also Additional Files 4, S2 Fig).

**S5 Table.** xlxs, (**A, C**)**Cluster analyses of transcripts with differential abundance between early development (1–12 h post-extrusion) and very late development in each egg type.**(A) AM, 13–14 h post-extrusion; (C) RE, 24–192 h post-extrusion). (B, D) The enriched KEGG pathways correspond with sheets A and C, respectively. A link (URL) to the corresponding KEGG map for each enriched pathway is presented in column L of sheets B and D. Enriched pathways related to diseases, vertebrate physiology, or plant physiology were removed from these datasets. Data shown in sheets A and C were extracted from Additional File 2, S2A Table (see also Fig 6 in the main text).

**S6 Table.**xlxs,  **The temporal transcript abundance patterns of transcripts encoding for dormancy hallmark proteins during the very late in RE development (24–192 h post extrusion).** The List of dormancy hallmark proteins (column A) was obtained from Ziv et al. [76] (Table 1) and shown in column BA**.**The nucleotide sequences of the old transcriptome contigs (column BA) were used in a BLASX search for the corresponding contigs in the Cel-seq transcriptome (column B). The NCBI accession number for each contig, the corresponding e-values for each BLASTX search, and the annotations are shown in columns C, D, and E, respectively. Column BC shows the fold-change in relative protein abundance levels between AM and RE proteins in Ziv et al. [76]. Data shown in this table were extracted from Additional File 2, S2A Table see also Fig 7C in the main text). For additional information, refer to Additional Files 3, S2 Text. (**B**) The temporal transcript abundance patterns of genes in the KEGG longevity regulating pathway (worm) show the transcript abundance of genes throughout development in AMs (1–14 h post-extrusion) and REs (1–192 h post-extrusion). The gene transcripts with moderate abundance (1.690<values<4.591) or high abundance ( >4.591) at 192 h in REs are highlighted in orange or yellow, respectively. Data shown in this table were extracted from Additional File 2, S2A Table (see also Additional Files 4, S3 Fig).

**S7 Table.** xlxs, **(A) The temporal transcript abundance patterns of genes associated with the following signaling pathways: Wnt, TGFβ, Notch, Hippo, FoxO, PI3-AKT, Insulin signaling, JAK-STAT, MAPK, AMPK, mTOR, and Hedgehog.** In the column corresponding with each pathway (columns AP–BA), a value of “1” indicates that the corresponding gene is associated with that pathway. In contrast, an empty cell indicates that the corresponding gene is not associated with that pathway. (**B**) The number of genes shared among signaling pathways. (**C**) The proportions of the shared genes found in each signaling pathway. Data shown in this table were extracted from Additional File 2, S2A Table (see also Fig 9 in the main text).

**S8 Table.**xlxs,**The temporal transcript abundance patterns of genes encoding for transcription factors in AMs and REs.**The List of transcription factors was assembled based on the literature [116]. Data shown in this table were extracted from Additional File 2, S2A Table.

**S9 Table.**xlxs, **The temporal transcript abundance patterns of homeobox genes in AMs and REs.** Columns C, D, E, and F present the corresponding NCBI accession number, the gene annotation, the e-value, and the identity score in the BLASTX. Data shown in this table were extracted from Additional File S2A, Table (see also Fig 10 in the main text). Empty lines indicate contigs identified in the reference transcriptome but not found in Additional File 2, S2A Table.

**S10 Table.**xlxs, **The temporal transcript abundance patterns of genes encoding for nuclear receptors in AMs and REs.** We used the List of nuclear receptors and the corresponding accession number from Kim et al. [97], except for *Hormone receptor 4, NR6A2. The contigs corresponding to Daf-12, Daf-36, Daf-41, and EcR are annotated in Additional File 2, S2A Table. Nuclear receptors that participate in molting are highlighted in orange. The Contigs with multiple alignments (e.g., c16888_g2, which was annotated as both Daf-12 and HR96) are highlighted in yellow. Data shown in this table were extracted from Additional File 2 S2A, Table.

**S11 Table.**xlxs, **The temporal transcript abundance patterns of (A) genes associated with lipid droplets (based on Olzmann and Carvalho [106]), as well as genes involved in KEGG lipid metabolic pathways: (B) fatty acid biosynthesis, (C) fatty acid elongation, (D) unsaturated fatty acid synthesis, and (E) fatty acid degradation, in REs and AMs.** Data shown in this table were extracted from Additional File 2, S2A Table.

**S12 Table.** xlxs, **The temporal transcript abundance patterns of genes involved in the (A) phototransduction, (B) circadian rhythm, and (C) circadian entrainment KEGG pathways in AMs and REs.**Opsin-annotated contigs are found at the bottom of sheet A. Data shown in this table were extracted from Additional File 2 S2A, Table.

**S13 Table.**xlxs, **List of opsin-associated genes in AMs and REs.**The corresponding RNA accession number and identity score from NCBI are shown. Five genes with high levels of sequence identity to B. koreanus sequences are shown in the right column but may have a different annotation than in Kim et al. [110].
