# Supplementary material for: A transcriptomic examination of encased rotifer embryos reveals the developmental trajectory leading to long-term dormancy; are they “animal seeds”?
Source: BMC Genomics. 2024 Jan 27;25:119. doi: 10.1186/s12864-024-09961-1 (PMC10821554; doi:10.1186/s12864-024-09961-1)
Supplement: Supplementary file 2 — Additional file 2: Tables S1. Embryonic developmental stages in AMs and REs, based on observations of time-lapse photographs. S2 Table. The temporal mean normalized gene counts (DeSeq2-VSD log-scale normalization) during the development of AMs (1–14 h post-extrusion) and REs (1–192 h post-extrusion). S3 Table. Cluster analyses (mean normalized transcript abundance values). S4 Table. (A) Cluster analysis of maternal gene transcripts (1-2 h after extrusion) with differential abundance between AMs and REs, and (B) the corresponding enriched KEGG pathways. S5 Table. (A, C) Cluster analyses of transcripts with differential abundance between early development (1–12 h post-extrusion) and very late development in each egg type. S6 Table. (A)The temporal transcript abundance patterns of transcripts encoding for dormancy hallmark proteins during the very late in RE development (24–192 h post extrusion). (B) The temporal transcript abundance patterns of genes in the KEGG longevity regulating pathway (worm) in AMs (1–14 h post-extrusion) and REs (1–192 h post-extrusion). S7 Table. (A) The temporal transcript abundance patterns of genes associated with the following signaling pathways: Wnt, TGFβ, Notch, Hippo, FoxO, PI3-AKT, Insulin signaling, JAK-STAT, MAPK, AMPK, mTOR, and Hedgehog. S8 Table. The temporal transcript abundance patterns of genes encoding for transcription factors in AMs and REs. S9 Table. The temporal transcript abundance patterns of homeobox genes in AMs and REs. S10 Table. The temporal transcript abundance patterns of genes encoding for nuclear receptors in AMs and REs. S11 Table. The temporal transcript abundance patterns of (A) genes associated with lipid droplets and genes involved in KEGG lipid metabolic pathways. S12 Table. The temporal transcript abundance patterns of genes involved in the (A) phototransduction, (B) circadian rhythm, and (C) circadian entrainment KEGG pathways in AMs and REs. S13 Table. List of opsin-associated genes in AMs and REs. [file 12864_2024_9961_MOESM2_ESM.zip › Additional File 2, S13 Table.docx]

**Additional File 2,**

Table S13. **List of opsin-associated genes expressed in AMs and REs.** The corresponding RNA accession number and identity score from NCBI are shown. Five genes with high levels of sequence identity with *B. koreanus* sequences are shown in the right column, but may have a different annotation than in Kim et al. [110].

| **Annotation** | **Contig number** | **Corresponding RNA accession number from NCBI** | **Gene name in NCBI** | **Identity score in NCBI** | **Corresponding *B. koreanus* mRNAs** |
| --- | --- | --- | --- | --- | --- |
| **PDE6D_HUMAN Retinal;**  **rod rhodopsin-sensitive**  **cGMP 3 5 -cyclic phosphodiesterase**  **subunit delta** | c4852_g1 | RNA01343.1 | retinal rod rhodopsin-sensitive cGMP 3 -5 -cyclic phosphor  diesterase  subunit delta | 1.00E-105 |  |
| **OPSD1_DROPS Opsin Rh1** | c23496_g1 | RNA30626.1 | melanopsin | 7.00E-179 |  |
| **OPN3; c-opsin** | c15896_g1 | RNA11184.1 | rhabdomeric | 5E-156 | KF885944.1 |
| **OPN4 Rh2_7;**  **r-opsin** | c18498_g1 | RNA24474.1 | arthropsin type | 7.00E-65 | KF885938.1 |
| **RHO OPN2; rhodopsin** | c16859_g1 | RNA30611.1 | rod opsin | 0 | KF88936.1 |
| **OPN4 Rh2_7;**  **r-opsin** | c22138_g1 | RNA24474.1 | arthropsin type | 0 | KF885935.1 |
| **RRH; visual pigment-like receptor peropsin** | C23548_g3 | RNA35867.1 | melanopsin | 0 | KF885939.1 |
