# Supplementary material for: A transcriptomic examination of encased rotifer embryos reveals the developmental trajectory leading to long-term dormancy; are they “animal seeds”?
Source: BMC Genomics. 2024 Jan 27;25:119. doi: 10.1186/s12864-024-09961-1 (PMC10821554; doi:10.1186/s12864-024-09961-1)
Supplement: Supplementary file 3 — Additional file 3: Text. S1. Bioinformatics. S2 Text. Transcript abundance profiles [116, 117]. [file 12864_2024_9961_MOESM3_ESM.zip › Additional File 3 S2 Text with references 120124_ESM.docx]

**Additional File 3: S2 Text**

**Transcript abundance profiles.**

**Exploration of the transcript abundance profiles of genes encoding for dormancy hallmark proteins, homeobox genes, genes encoding transcription factors, nuclear receptors, and the construction of KEGG maps of the signaling pathways, lipid droplets, lipid metabolism, phototransduction, circadian rhythm, and the circadian entrainment pathways**

Dormancy hallmark proteins: The list of dormancy hallmark proteins was obtained from Ziv et al. [76] (Table 1). To identify corresponding genes in Additional File 2, S2A Table, the nucleotide sequences of contigs from the transcriptome of Ziv et al. [76] were used in a BLASTX search for the corresponding contigs in the Cel-seq transcriptome of the current study. The temporal transcript abundance patterns of genes during development in AMs (1–14 h PE) and REs (1–192 h PE) were extracted from Additional File 2, S2A Table and presented in Additional File 2, S6 Table and 7C Fig in the text.

To identify genes associated with the following pathways: homeobox genes, genes encoding transcription factors, nuclear receptors, and lipid droplets, we searched for the RNA sequence of each gene at <https://www.ncbi.nlm.nih.gov/nuccore>. The RNA sequences (almost all of them were found in the translated genome of *B. plicatilis*;

<https://www.ncbi.nlm.nih.gov/nuccore/REGN01000000>) were used by BLASTX to identify the corresponding contigs in the reference transcriptome. The contigs (genes) for each pathway were extracted from Additional File 2, S2A Table to form a table containing the temporal transcript abundance patterns of genes in AMs and REs during development and the differential transcript abundance of genes for each pathway. For KEGG pathways, the KO or K1 annotations in Additional File 2, SA2 Table, were used to form tables and KEGG maps corresponding with each pathway. The KEGG: Kyoto Encyclopedia of Genes and Genomes (<https://www.genome.jp/kegg/>) was used for formation of KEGG maps in this study [116].

This way, we obtained the transcript abundance patterns in the Additional File 2, S7-S12 Tables and Additional File 4, S3-S7 Figs.

Signaling pathways: The following signaling pathways were investigated: Wnt, Notch, TGFβ, Hippo, Hedgehog, AKT, JAK-STAT, MAPK, Insulin signaling, FoxO, and AMPK. The temporal transcript abundance patterns of genes in AMs and REs during development and the differential transcript abundance patterns were extracted from Additional File 2, S2A Table and are shown in Additional File 2, S7 Table. These genes were used to construct the KEGG maps shown in Additional File 4, S5 Fig.

Transcription factors: A list of transcription factors (excluding homeobox genes and nuclear receptors), was obtained from Imai et al. [117]. This list included transcription factors in the bHLH, bZip, Ets, Fox, HMG, and T-box families. Because the zinc-finger motif cannot be used as evidence that a gene encodes a transcription factor [117], the list did not include genes with a zinc-finger motif. The temporal transcript abundance patterns in AMs and REs during development and the differential trascriptt abundance patterns were extracted from Additional File 2, S2A Table and are shown in Additional File 2, S8 Table.

Homeobox genes: To identify homeobox transcripts , the RNA sequences were obtained as described above of known homeobox-class genes (i.e., Hox, Parahox, NK, Tale and Sine, Paired and Paired-related, LIM, Zinc-finger, POU, HD-CUT, and Prospero). The transcript abundance profiles during development and for the differential transcript abundance patterns are shown in Additional File 2, S9 Table and Fig 10 in the text.

Nuclear receptors: A list of nuclear receptor genes was extracted from Kim et al. [97]. The contigs corresponding with these nuclear receptor genes were identified by searching for the RNA sequence of each receptor as described above. The genes (contigs) corresponding with these RNAs were extracted from Additional File 2, S2A Table, and the temporal transcript abundance patterns in AMs and REs during development and the differential transcript abundance patterns are shown in Additional File 2, S10 Table. We also searched for nuclear receptors corresponding to those previously identified in *C. elegans* and *Drosophila melanogaster* based on previously published lists [4, 98] and gene annotations in the reference transcriptome and Additional File 2, S2A Table (Description and Pathways_info columns). Phylogenetic comparisons of RNA sequences corresponding to some of the contigs in Additional File 2, S10 Table were performed using SMARTBLAST (<https://blast.ncbi.nlm.nih.gov/smartblast/>, results not shown).

Lipid droplets and lipid metabolism: A list of proteins associated with lipid droplets was constructed based on a previous study [106]. The temporal transcript abundance patterns of the annotations matching these genes in AMs and REs during development and the differential transcript abundance patterns were extracted from Additional File 2, S2A Table and are shown in Additional File 2, S11 Table. The transcript abundance profiles and the differential transcript abundance patterns associated with KEGG lipid metabolism (i.e., fatty acid biosynthesis, fatty acid elongation, biosynthesis of unsaturated fatty acids, and fatty acid degradation) are also shown in Additional File 2, S11 Table, and Additional File 4, S6 Fig.

Phototransduction, circadian rhythm, and circadian entrainment pathways: The contigs (genes) for each pathway were extracted from the reference transcriptome and Additional File 2, S2A Table (as described above) to form a table (Additional File 2, S12 Table) containing the temporal transcript abundance patterns in AMs and REs during development and a table with the list of opsins (Additional File 2, S13 Table). KEGG maps are shown in Additional File 4, S7 Fig.

**Reference:**

1. Kanehisa M, Furumichi M, Sato Y, Kawashima M, Ishiguro-Watanabe M. KEGG for taxonomy-based analysis of pathways and genomes. Nucleic Acids Res. 2023 Jan 6;51(D1):D587-D592. doi: 10.1093/nar/gkac963.
2. Imai KS, Hino K, Yagi K, Satoh N, Satou Y. Transcript abundanceprofiles of transcription factors and signaling molecules in the ascidian embryo: towards a comprehensive understanding of gene networks. Development. 2004;131(16):4047-4058. doi: 10.1242/dev.0127
