# Supplementary material for: A transcriptomic examination of encased rotifer embryos reveals the developmental trajectory leading to long-term dormancy; are they “animal seeds”?
Source: BMC Genomics. 2024 Jan 27;25:119. doi: 10.1186/s12864-024-09961-1 (PMC10821554; doi:10.1186/s12864-024-09961-1)
Supplement: Supplementary file 3 — Additional file 3: Text. S1. Bioinformatics. S2 Text. Transcript abundance profiles [116, 117]. [file 12864_2024_9961_MOESM3_ESM.zip › Additional File 3, S1 Text.docx]

**Additional File 3: S1 Text**

**Bioinformatics**

**RNA-Seq bioinformatic analysis**

1. Reference transcriptome assembly: Pooled RNA samples collected from rotifers at 13 developmental stages were used for a transcriptome assembly. From 10 ppt ASW cultures displaying sexual reproduction, the following samples were collected (at least 50 individuals or eggs per sample): amictic females, unfertilized mictic females carrying male eggs, fertilized mictic females carrying resting eggs, males, and newly formed REs. In addition, the following samples were collected: REs that were stored for three months and 2.5 years, REs that were stored for three months and exposed to illumination (which induced an exit from dormancy and hatching) for 4, 16, and 31 h, and neonates that hatched from these REs after 36 -48 h. Amictic females carrying amictic eggs were also collected from 40 ppt ASW cultures, where only amictic females are formed as there is no sexual reproduction in these cultures. Pooled samples were sequenced on an Illumina HiSeq2500, yielding 511 million 100-bp paired-end sequences. Estimation of raw-sequence quality using FastQC (<https://www.bioinformatics.babraham.ac.uk/projects/fastqc/>) indicated that the reads were of very high-quality throughout their length. Trimmomatic v. 0.32 (<http://www.usadellab.org/cms/?page=trimmomatic>)

was used to remove the first 15 bases of each read (HEADCROP:15). De novo assembly was carried out using Trinity (v. trinityrnaseq_r20140717, min_kmer_cov = 2; 

(<https://github.com/trinityrnaseq/trinityrnaseq/wiki>). The total number of transcripts was 50,628 (N50 = 1,946; L50 = 6,297), and 85% of all reads were successfully aligned back to the reference transcriptome. Of the successfully aligned reads, 89.4% were uniquely mapped. The rotifer reference transcriptome has been submitted to NCBI, Accession PRJNA821380 ID 821380 at <https://www.ncbi.nlm.nih.gov/bioproject/PRJNA821380> (SRA samples 156-171, SRR215415774-SRR21541589).

2. Reference transcriptome annotation: The longest isoform for each gene in the transcriptome was chosen for transcriptome annotation. First, a BLASTX (v. 2.2.25+;

<https://blast.ncbi.nlm.nih.gov/Blast.cgi>) search was carried out to identify orthologous sequences in the Swiss-Prot database (e-value cutoff 1E-7). Blast2Go (<https://www.blast2go.com/>) was then used to annotate the transcriptome and to associate Gene Ontology (GO) terms with the transcripts. A local InterProScan search against Pfam, PIRSF, and PROSITE was also used to annotate further transcripts that could not be assigned GO terms in the Blast2Go analysis. Of the 35,242 putative genes identified in the transcriptome, 12,201 transcripts were successfully annotated using Blast (34.6%). Fragmentation may explain the low annotation ratio as filtering out transcripts shorter than 500 nucleotides increased the annotated fraction to 65% (10,684/16,339).

Of the 12,201 transcripts successfully annotated using Blast, 11,569 (94.8%) were associated with one or more GO terms. Finally, we functionally annotated these 11,569 genes using the KEGG Automatic Annotation Server (KAAS) (https://www.genome.jp/kegg/kaas/)[1]

KEGG Orthology (KO) IDs were successfully assigned to 7,567 transcripts (65.4%)( The complete GO map was constructed using the GO.db R package

 (<https://bioconductor.org/packages/release/data/annotation/html/GO.db.html>) and KO IDs were converted to pathways using the KEGGREST R package

 (<https://bioconductor.org/packages/release/bioc/html/KEGGREST.html>)

3. CEL-Seq: CEL-Seq data were obtained and cleaned by the Technion Genome Center (ITT-Technion, Haifa, Israel). In brief, 155 single-embryo samples were sequenced (70 AM and 85REs), yielding an average of 2.5 M raw reads per sample. After demultiplexing, the raw data (fastq format) were cleaned by removing low-quality sequences (Phred score < 20). We trimmed adaptor sequences (based on the small RNA 5' adaptor, GATCGTCGGACT) and poly-A ends using the Trim Galore wrapper (https://www.bioinformatics.babraham.ac.uk/projects/trim_galore for Cutadapt v 1.10 (<https://github.com/marcelm/cutadapt/>). Only sequences longer than 30 bp after trimming were retained. Cleaning removed an average of 23.9% of the total reads per sample, and the remaining reads had an average length of 36.6 bp. The CEL-Seq transcriptome was assembled as described below in 4. The CEL-seq transcriptome reads were submitted to NCBI, Accession PRJNA821380 ID 821380 at <https://www.ncbi.nlm.nih.gov/bioproject/PRJNA821380> (SRA samples 1-155, SRR18532591-SRR18532745).

4. CEL-seq mapping, counting, quality control, and normalization: Clean, high-quality reads were aligned to the assembled reference transcriptome using Bowtie2 (<https://bowtie-bio.sourceforge.net/bowtie2/index.shtml>, v.2.2.6) and the read counts per gene were calculated using RSEM (<https://github.com/deweylab/RSEM>, v1.2.25). We used the scater (single-cell analysis toolkit for gene expression data in R) package to filter out low-quality count data: small libraries, libraries with few expressed genes, and genes with very low average counts were removed. After cleaning and quality control, 136 samples and 14,779 genes were retained (19 samples and 20,463 genes were filtered out). Cleaned count data was normalized, and clustering, principal component analysis, and visualization were performed using the variance stabilizing transformation function of DESeq2

 (<https://bioconductor.org/packages/release/bioc/html/DESeq2.html>). To identify differential abundance of gene transcripts (expressed genes), cleaned or raw count data were submitted to DESeq2, and normalization was performed as part of the statistical modeling. The minimum and maximum normalized transcript abundance levels were 0.9484 and 18.9718, respectively (Additional Files 2, S2A Table).

5. Hierarchical clustering analysis, cluster visualization, and enrichment: Hierarchical clustering analysis was performed on each dataset of differential abundance of gene transcripts. The distance matrix used for clustering was created using Pearson correlations. Clusters were agglomerated using the ward.D function in the hclust R package

(<https://www.rdocumentation.org/packages/stats/versions/3.6.2/topics/hclust>)

and partitioned using the mclust R package

 (<https://cran.r-project.org/web/packages/mclust/citation.html>). Clusters were visualized using the pheatmap and ggplot2 R packages (<https://cran.r-project.org/web/packages/pheatmap/>). Using the annotated reference transcriptome, we identified GO terms and KEGG pathways significantly enriched in the CEL-seq gene clusters with the clusterProfiler R package.

 (<https://bioconductor.org/packages/release/bioc/html/clusterProfiler.html>). Enrichment p-values were corrected for multiple testing using the BH method.

 (<https://www.bioinformatics.babraham.ac.uk/projects/fastqc/>). Representative GO terms (i.e., the most significant GO term out of each group of similar enriched terms) were identified using the simplify function in the clusterProfiler R package.

 (<https://bioconductor.org/packages/release/bioc/html/clusterProfiler.html>).

6. Differential transcript abundance models: We identified differential transcript abundance expressed genes using the following four sets of models in DESeq2 (Additional Files 2, S2A Table):

(1) Genes with differential abundance throughout AM or RE development (1-12 h PE). For this analysis, we used raw, uncleaned data (i.e., all 155 samples and 35,242 genes).

Complete model: Count ~ time point [all time points in the cell type]

Reduced model: Count ~ 1

We then used the likelihood ratio test (LTR) to identify genes with expression profiles that changed significantly during development for each egg type (FDR-adjusted p-value < 0.01).

(2) Genes with a temporal transcript abundance pattern that differed significantly between AMs and REs over 1 -12 h PE of development. For this analysis, we used the cleaned count data (136 samples and 14,779 genes).

Full model: Count ~ time point [1 to 12] + cell type [RE or AM] + time point: cell type

Reduced model: Count ~ time point [1 to 12] + cell type [RE or AM]

The comparison was between counts of 1-12 h of AM and 1-12 h of RE. We used the LRT to identify gene transcripts in which the interaction term [time point: cell type] (which was not present in the reduced model) contributed significantly to the likelihood of the dataset.

(3) Genes with significant differential transcript abundance between REs and AMs very early in development (1-2 h PE). For this analysis we used the cleaned count data (136 samples and 14,779 genes.

Model: Count ~ TIME&CELL groups [AM Very arly; AM Late; RE Very Early; RE Late]

Contrast: [AM Very Early vs. RE Very Early]

Very Early=time points<=12; Late =time points>12

We then used contrasts to identify gene transcripts withsignificantly differential abundance between REs and AMs very early in development.

(4) Genes with differential transcript abundance in the very late stages of development (> 12 h PE) as compared to the earlier stages of development (1–12 h PE) for each egg type. For this analysis, we used the raw, uncleaned data (i.e., all 155 samples and 35,242 genes).

Model: Count ~ TIME&CELL groups [AM Early; AM Very late; RE Early; RE Very Late]

Contrast: [AM Early vs. AM Very late] and [RE Early vs. RE Very late]

Early=time points<=12; Very late =time points>12

We then used contrasts to identify gene transcripts with significant differential transcript abundance between the early and the very late stages of development for each egg type.

In all DESeq2 analyses, p-values were adjusted for the false discovery rate (FDR), and we considered FDR-adjusted p-values < 0.01 statistically significant. DESeq2 also automatically uses independent filtering to maximize the number of gene transcripts with significant p-values.
