# Supplementary material for: A transcriptomic examination of encased rotifer embryos reveals the developmental trajectory leading to long-term dormancy; are they “animal seeds”?
Source: BMC Genomics. 2024 Jan 27;25:119. doi: 10.1186/s12864-024-09961-1 (PMC10821554; doi:10.1186/s12864-024-09961-1)
Supplement: Supplementary file 4 — Additional file 4: Figure. S1. Number of reads sequenced per sample across three REs and AMs developmental stages. S2 Fig. Comparison of the transcript abundance profiles of putative maternal genes between AMs and REs. S3 Fig. The Longevity pathway (worm) highlights differential transcript abundance between AM and RE (left panel) and highly abundant protein-encoding genes at 192 h in RE (right panel). S4 Fig. Very highly abundant gene transcripts (>4.251) of energy-yielding KEGG pathways. (>4.251) at 192 hr of RE. S5 Fig. KEGG signaling pathways (maps) highlighting protein-encoding genes with differential transcript abundance between AM and RE (left panel) and highly abundant transcripts at 192 hr in RE (right panel). S6 Fig. Lipid metabolism KEGG pathways (maps) highlighting protein-encoding genes with differential transcript abundance between AM and RE (left panels) and highly abundant transcripts at 192 hr in RE (right panel). S7 Fig. Light responding KEGG pathways (maps) highlighting protein-encoding genes with differential transcript abundance between AM and RE (left panels) and transcripts with high abundance at 192 hr in RE (right panels). [file 12864_2024_9961_MOESM4_ESM.zip › Additional File 4, S1 Fig.pdf]

Additional File 4

S1 Fig. pdf: **Number of reads sequenced per sample across three REs and AMs developmental stages.** Early (AM: 1–3 h post extrusion; RE: 1–5 h post-extrusion), middle (AM: 4–8 h post-extrusion; RE: 6–8 h post-extrusion), and late/very late (RE and AM: >9 h post-extrusion)**.** Each point represents a single embryo, and this analysis was performed on the raw (un-normalized) data, considering sequencing depth. Samples with less than 400,000 raw reads were removed from the analysis. The number of reads was counted for each sample using at least two raw reads per gene. There was no significant difference in gene numbers between AMs and REs during early development (p=0.78, Student's t-test). However, the gene number differed significantly between AMs and REs in the middle developmental stage (p=0.0018, Student's t-test) and during late development (p=4.99e-10, Student's t-test).

Fig S1

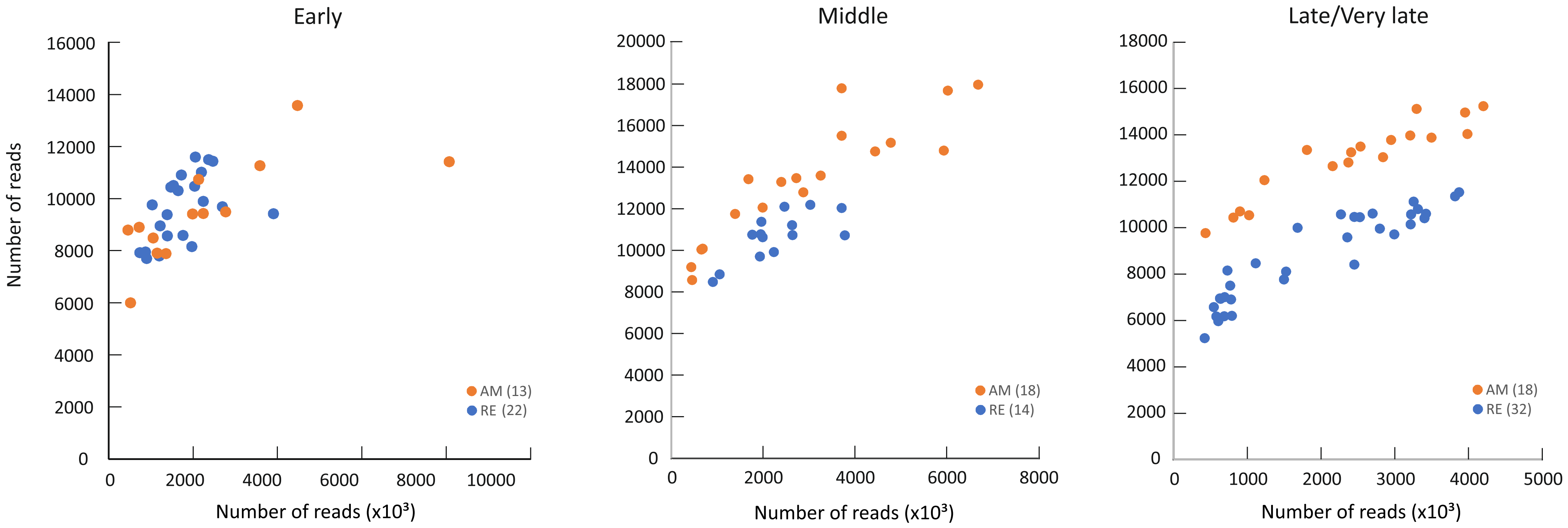
Early
Middle
Late/Very late
Number of reads
Number of reads (x10³)
AM (13)
RE (22)
AM (18)
RE (14)
AM (18)
RE (32)
