# Supplementary material for: A transcriptomic examination of encased rotifer embryos reveals the developmental trajectory leading to long-term dormancy; are they “animal seeds”?
Source: BMC Genomics. 2024 Jan 27;25:119. doi: 10.1186/s12864-024-09961-1 (PMC10821554; doi:10.1186/s12864-024-09961-1)
Supplement: Supplementary file 4 — Additional file 4: Figure. S1. Number of reads sequenced per sample across three REs and AMs developmental stages. S2 Fig. Comparison of the transcript abundance profiles of putative maternal genes between AMs and REs. S3 Fig. The Longevity pathway (worm) highlights differential transcript abundance between AM and RE (left panel) and highly abundant protein-encoding genes at 192 h in RE (right panel). S4 Fig. Very highly abundant gene transcripts (>4.251) of energy-yielding KEGG pathways. (>4.251) at 192 hr of RE. S5 Fig. KEGG signaling pathways (maps) highlighting protein-encoding genes with differential transcript abundance between AM and RE (left panel) and highly abundant transcripts at 192 hr in RE (right panel). S6 Fig. Lipid metabolism KEGG pathways (maps) highlighting protein-encoding genes with differential transcript abundance between AM and RE (left panels) and highly abundant transcripts at 192 hr in RE (right panel). S7 Fig. Light responding KEGG pathways (maps) highlighting protein-encoding genes with differential transcript abundance between AM and RE (left panels) and transcripts with high abundance at 192 hr in RE (right panels). [file 12864_2024_9961_MOESM4_ESM.zip › Additional File 4, S2 Fig.pdf]

Additional File 4

S2 Fig. pdf: **Comparison of the transcript abundance profiles of putative maternal genes between AMs and REs.** (**A**) Heatmaps of the expression patterns of genes that were differentially expressed in early development (1-2 h post-extrusion), showing the seven identified clusters. (**B**) The expression pattern of each cluster and the corresponding enriched pathways. The colored circles prepending each pathway indicate the statistical significance of the enrichment (color) and the relative proportion of total genes (size). The data is presented in Additional File 2, S4 Table.

Fig S2

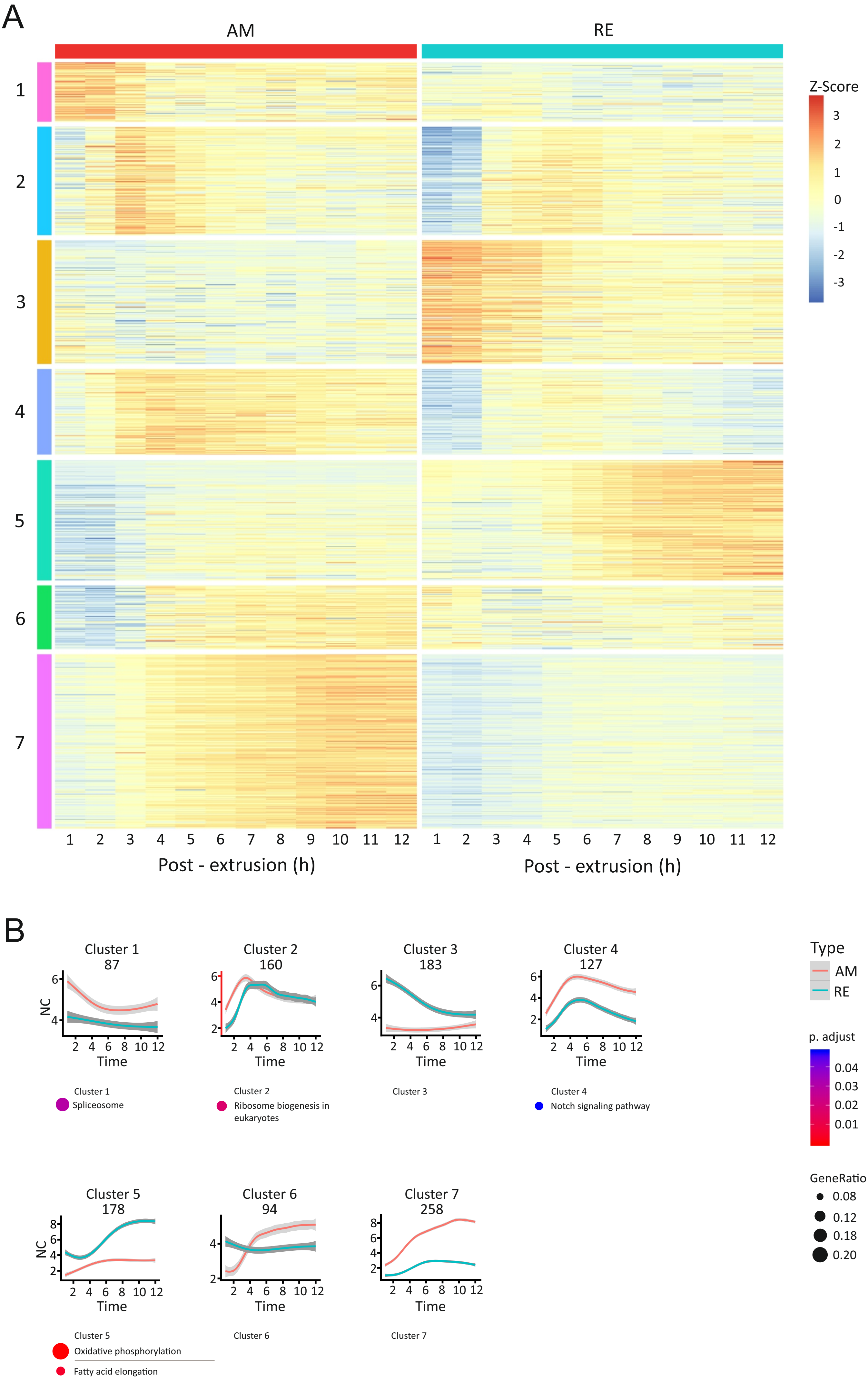

A
AM
RE
1
2
3
4
5
6
7
Z-Score
3
2
1
0
-1
-2
-3
Post - extrusion (h)
Post - extrusion (h)
B
Cluster 1
87
Cluster 2
160
Cluster 3
183
Cluster 4
127
Cluster 5
178
Cluster 6
94
Cluster 7
258
NC
Time
Type
AM
RE
p. adjust
0.04
0.03
0.02
0.01
GeneRatio
0.08
0.12
0.18
0.20
Cluster 1
Spliceosome
Cluster 2
Ribosome biogenesis in eukaryotes
Cluster 3
Cluster 4
Notch signaling pathway
Cluster 5
Oxidative phosphorylation
Fatty acid elongation
Cluster 6
Cluster 7
