# Supplementary material for: A transcriptomic examination of encased rotifer embryos reveals the developmental trajectory leading to long-term dormancy; are they “animal seeds”?
Source: BMC Genomics. 2024 Jan 27;25:119. doi: 10.1186/s12864-024-09961-1 (PMC10821554; doi:10.1186/s12864-024-09961-1)
Supplement: Supplementary file 4 — Additional file 4: Figure. S1. Number of reads sequenced per sample across three REs and AMs developmental stages. S2 Fig. Comparison of the transcript abundance profiles of putative maternal genes between AMs and REs. S3 Fig. The Longevity pathway (worm) highlights differential transcript abundance between AM and RE (left panel) and highly abundant protein-encoding genes at 192 h in RE (right panel). S4 Fig. Very highly abundant gene transcripts (>4.251) of energy-yielding KEGG pathways. (>4.251) at 192 hr of RE. S5 Fig. KEGG signaling pathways (maps) highlighting protein-encoding genes with differential transcript abundance between AM and RE (left panel) and highly abundant transcripts at 192 hr in RE (right panel). S6 Fig. Lipid metabolism KEGG pathways (maps) highlighting protein-encoding genes with differential transcript abundance between AM and RE (left panels) and highly abundant transcripts at 192 hr in RE (right panel). S7 Fig. Light responding KEGG pathways (maps) highlighting protein-encoding genes with differential transcript abundance between AM and RE (left panels) and transcripts with high abundance at 192 hr in RE (right panels). [file 12864_2024_9961_MOESM4_ESM.zip › Additional File 4, S3 Fig.pdf]

Additional File 4

S3 Fig. pdf: **The Longevity pathway (worm) highlights differential transcript abundance between AM and RE (left panel) and highly abundant protein-encoding genes at 192 h in RE (right panel).** Blue colored boxes in the left panel represent transcripts with differential abundance between AM and RE (AM vs. RE, $p<0.01$). In the right panel, dark-colored mauve boxes represent protein-encoding genes with values >4.951, while light-colored mauve boxes represent protein-encoding genes with values >1.690. Yellow boxes represent transcripts identified in Additional File 2, S2A Table. but not in one of the patterns described above, while blank boxes represent protein-encoding genes that are present in the KEGG map but were not identified in Additional File 2, S2A Table.

Fig S3

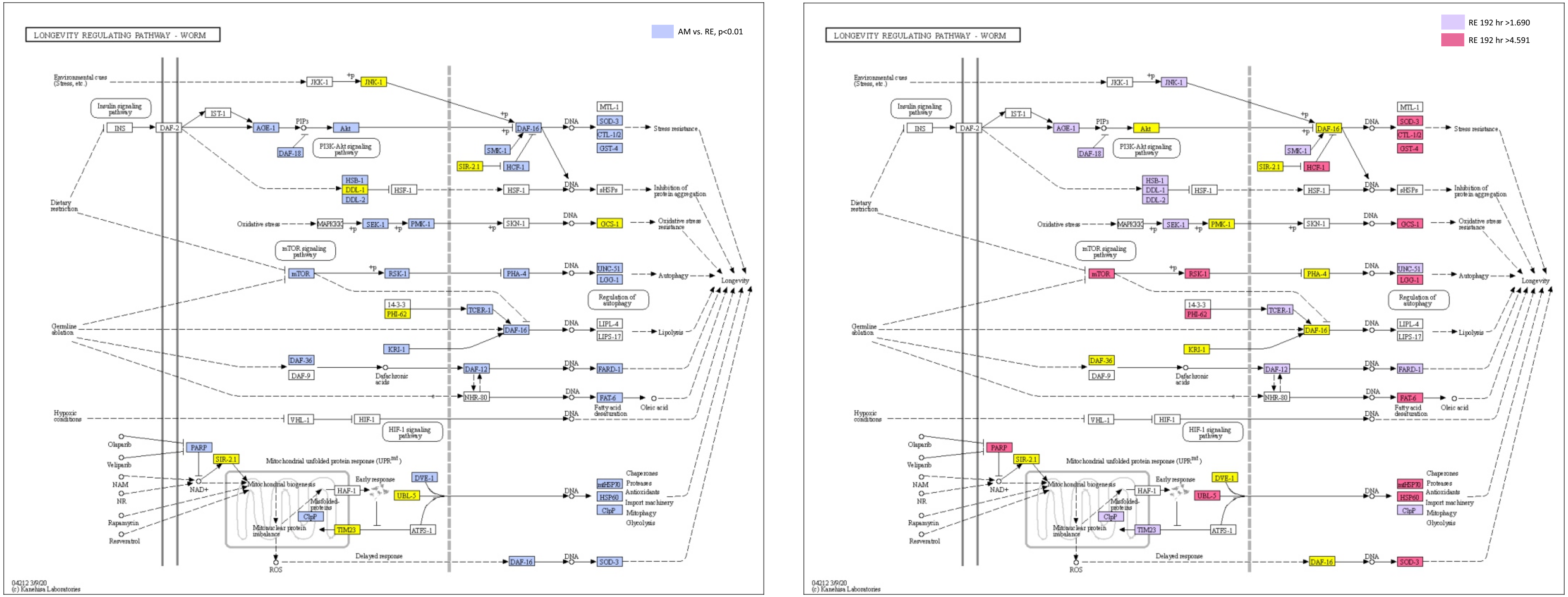
LONGEVITY REGULATING PATHWAY - WORM
AM vs. RE, p<0.01
Environmental cues (Stress, etc.)
JKK-1
JNK-1
Insulin signaling pathway
INS
DAF-2
IST-1
AGE-1
PIP3
Akt
DAF-18
PI3K-Akt signaling pathway
DAF-16
SMK-1
SIR-2.1
HCF-1
DNA
MTL-1
SOD-3
CTL-1/2
GST-4
Stress resistance
HSB-1
DDL-1
DDL-2
HSF-1
sHSPs
Inhibition of protein aggregation
Dietary restriction
Oxidative stress
MAPKKK
SEK-1
PMK-1
SKN-1
GCS-1
Oxidative stress resistance
mTOR signaling pathway
mTOR
RSK-1
PHA-4
UNC-51
LGG-1
Autophagy
Longevity
Regulation of autophagy
14-3-3
PHI-62
TCER-1
Germline ablation
KRI-1
LIPL-4
LIPS-17
Lipolysis
DAF-36
DAF-9
Dafachronic acids
DAF-12
FARD-1
NHR-80
FAT-6
Fatty acid desaturation
Oleic acid
Hypoxic conditions
VHL-1
HIF-1
HIF-1 signaling pathway
Olaparib
Veliparib
NAM
NR
Rapamycin
Resveratrol
PARP
NAD+
Mitochondrial unfolded protein response (UPRmt)
Mitochondrial biogenesis
Early response
HAF-1
DVE-1
UBL-5
Misfolded-proteins
ClpP
Mitonuclear protein imbalance
TIM23
ATFS-1
mtHSP70
HSP60
Chaperones
Proteases
Antioxidants
Import machinery
Mitophagy
Glycolysis
Delayed response
ROS
04212 3/9/20
(c) Kanehisa Laboratories
LONGEVITY REGULATING PATHWAY - WORM
RE 192 hr >1.690
RE 192 hr >4.591
04212 3/9/20
(c) Kanehisa Laboratories
