# Supplementary material for: A transcriptomic examination of encased rotifer embryos reveals the developmental trajectory leading to long-term dormancy; are they “animal seeds”?
Source: BMC Genomics. 2024 Jan 27;25:119. doi: 10.1186/s12864-024-09961-1 (PMC10821554; doi:10.1186/s12864-024-09961-1)
Supplement: Supplementary file 4 — Additional file 4: Figure. S1. Number of reads sequenced per sample across three REs and AMs developmental stages. S2 Fig. Comparison of the transcript abundance profiles of putative maternal genes between AMs and REs. S3 Fig. The Longevity pathway (worm) highlights differential transcript abundance between AM and RE (left panel) and highly abundant protein-encoding genes at 192 h in RE (right panel). S4 Fig. Very highly abundant gene transcripts (>4.251) of energy-yielding KEGG pathways. (>4.251) at 192 hr of RE. S5 Fig. KEGG signaling pathways (maps) highlighting protein-encoding genes with differential transcript abundance between AM and RE (left panel) and highly abundant transcripts at 192 hr in RE (right panel). S6 Fig. Lipid metabolism KEGG pathways (maps) highlighting protein-encoding genes with differential transcript abundance between AM and RE (left panels) and highly abundant transcripts at 192 hr in RE (right panel). S7 Fig. Light responding KEGG pathways (maps) highlighting protein-encoding genes with differential transcript abundance between AM and RE (left panels) and transcripts with high abundance at 192 hr in RE (right panels). [file 12864_2024_9961_MOESM4_ESM.zip › Additional File 4, S4 Fig.pdf]

**Additional File 4**

S4 Fig. pdf: **Very highly abundant gene transcripts (>4.251) of energy-yielding KEGG pathways**. **(>4.251) at 192 hr of RE**. (**A**) Glycolysis/gluconeogenesis, (**B**) Citrate cycle (TCA), (**C**) Pyruvate metabolism, (**D**) Pentose phosphate pathway, (**E**) Carbon metabolism and (**F**) Oxidative phosphorylation. The KEGG maps were formed from data presented in Additional File 2, S2F Table. Dark-colored mauve boxes represent protein-encoding transcripts with values >4.951. Light- colored mauve boxes represent gene transcripts with values >1.690. Yellow boxes represent gene transcript that were identified in Additional File 2, S2A Table but not in one of the patterns described above, while blank boxes represent protein-encoding genes that are present in the KEGG map but were not identified in Additional File 2, S2A Table.

Fig S4

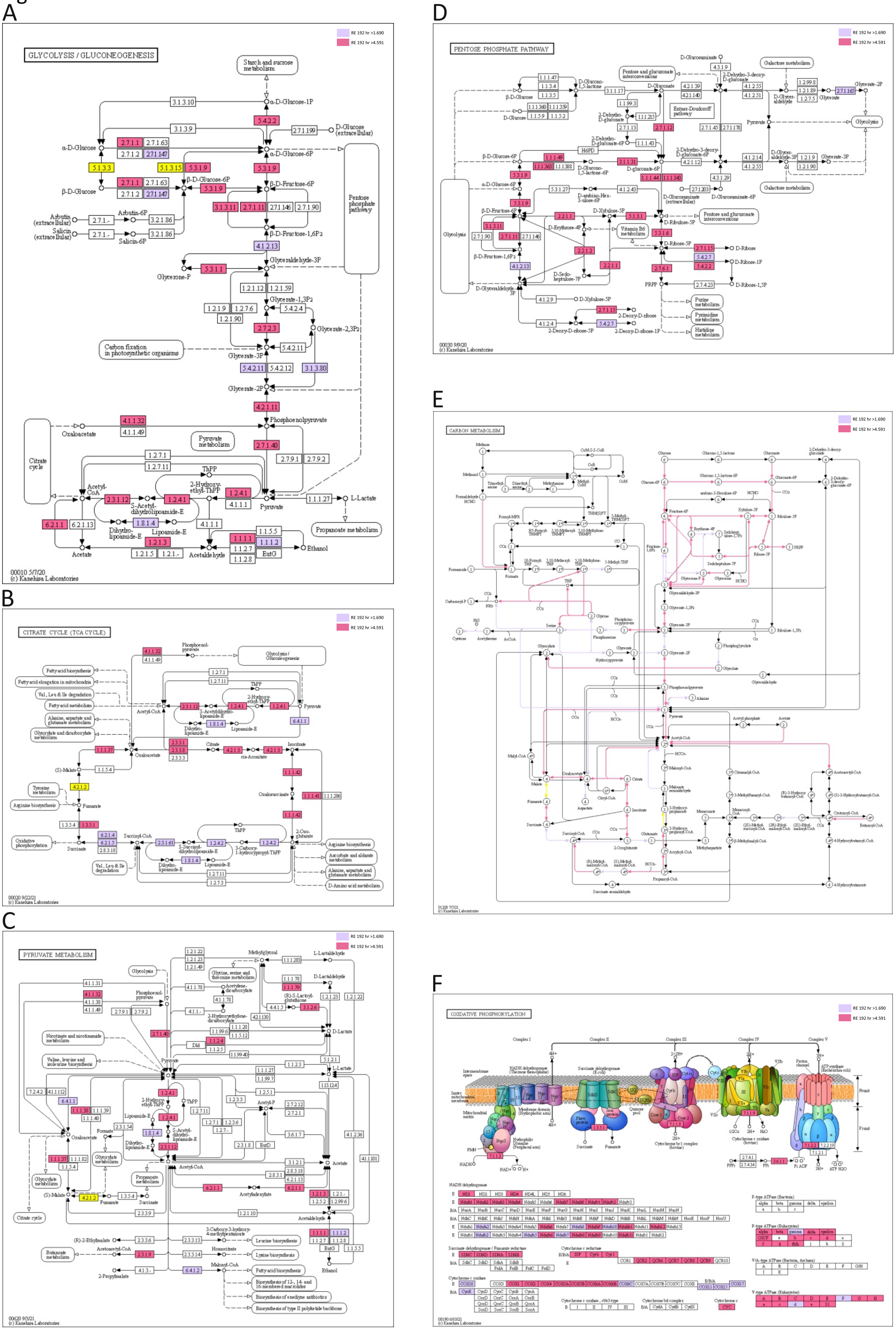
A
GLYCOLYSIS / GLUCONEOGENESIS
RE 192 hr >1.690
RE 192 hr >4.591
B
CITRATE CYCLE (TCA CYCLE)
C
PYRUVATE METABOLISM
D
PENTOSE PHOSPHATE PATHWAY
E
CARBON METABOLISM
F
OXIDATIVE PHOSPHORYLATION
