# Supplementary material for: A transcriptomic examination of encased rotifer embryos reveals the developmental trajectory leading to long-term dormancy; are they “animal seeds”?
Source: BMC Genomics. 2024 Jan 27;25:119. doi: 10.1186/s12864-024-09961-1 (PMC10821554; doi:10.1186/s12864-024-09961-1)
Supplement: Supplementary file 4 — Additional file 4: Figure. S1. Number of reads sequenced per sample across three REs and AMs developmental stages. S2 Fig. Comparison of the transcript abundance profiles of putative maternal genes between AMs and REs. S3 Fig. The Longevity pathway (worm) highlights differential transcript abundance between AM and RE (left panel) and highly abundant protein-encoding genes at 192 h in RE (right panel). S4 Fig. Very highly abundant gene transcripts (>4.251) of energy-yielding KEGG pathways. (>4.251) at 192 hr of RE. S5 Fig. KEGG signaling pathways (maps) highlighting protein-encoding genes with differential transcript abundance between AM and RE (left panel) and highly abundant transcripts at 192 hr in RE (right panel). S6 Fig. Lipid metabolism KEGG pathways (maps) highlighting protein-encoding genes with differential transcript abundance between AM and RE (left panels) and highly abundant transcripts at 192 hr in RE (right panel). S7 Fig. Light responding KEGG pathways (maps) highlighting protein-encoding genes with differential transcript abundance between AM and RE (left panels) and transcripts with high abundance at 192 hr in RE (right panels). [file 12864_2024_9961_MOESM4_ESM.zip › Additional File 4, S5 Fig.pdf]

**Additional File 4**

S5 Fig. pdf: **KEGG signaling pathways (maps) highlighting protein-encoding genes with differential transcript abundance between AM and RE (left panel) and highly abundant transcripts at 192 hr in RE (right panel).** The pathways include; (**A**) AMPK, (**B**) FOXO, (**C**) Hedgehog, (**D**) Hippo, (**E**) Insulin, (**F**) JAK-STAT, (**G**) MAPK, (**H**) mTOR, (**I**) Notch, (**J**) PI3K-AKT, (**K**) TGF-beta, and (**L**) Wnt, Blue colored boxes in the left panel represent transcripts with differential abundance between AM and RE (AM vs. RE, $p<0.01$). In the right panel, dark-colored mauve boxes represent protein-encoding gene transcripts with values >4.951, while light-colored mauve boxes represent protein-encoding gene transcripts with values >1.690. Yellow boxes represent gene transcripts identified in Additional File 2, S2A Table but not in one of the patterns described above, while blank boxes represent protein-encoding genes that are present in the KEGG map but were not identified in Additional File 2, S2A Table.

Fig S5

A

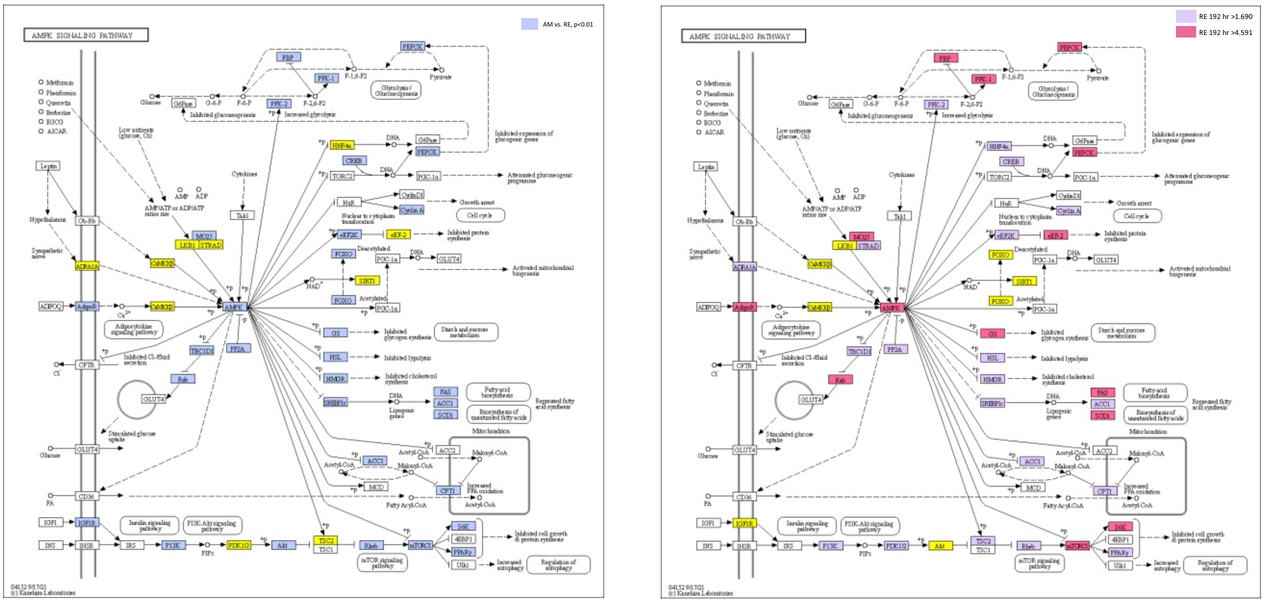

AMPK SIGNALING PATHWAY
AM vs. RE, p<0.01
RE 192 hr >1.690
RE 192 hr >4.591


B

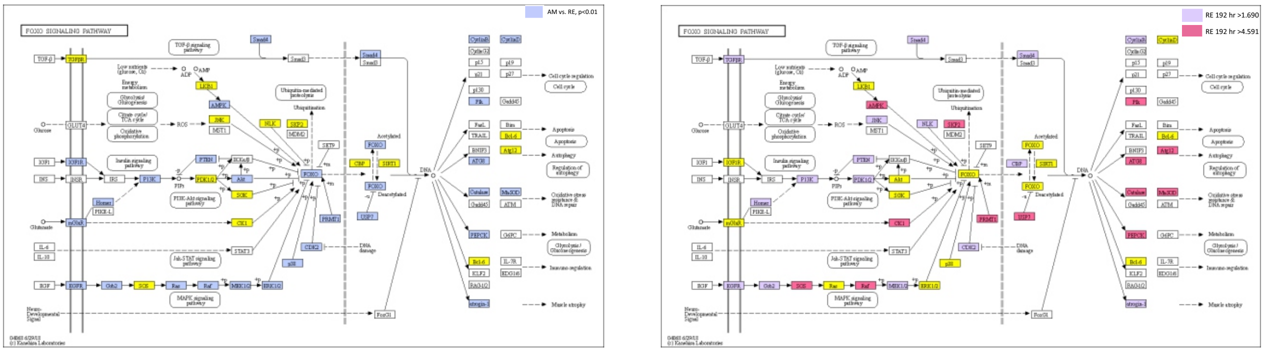

FOXO SIGNALING PATHWAY
AM vs. RE, p<0.01
RE 192 hr >1.690
RE 192 hr >4.591


C

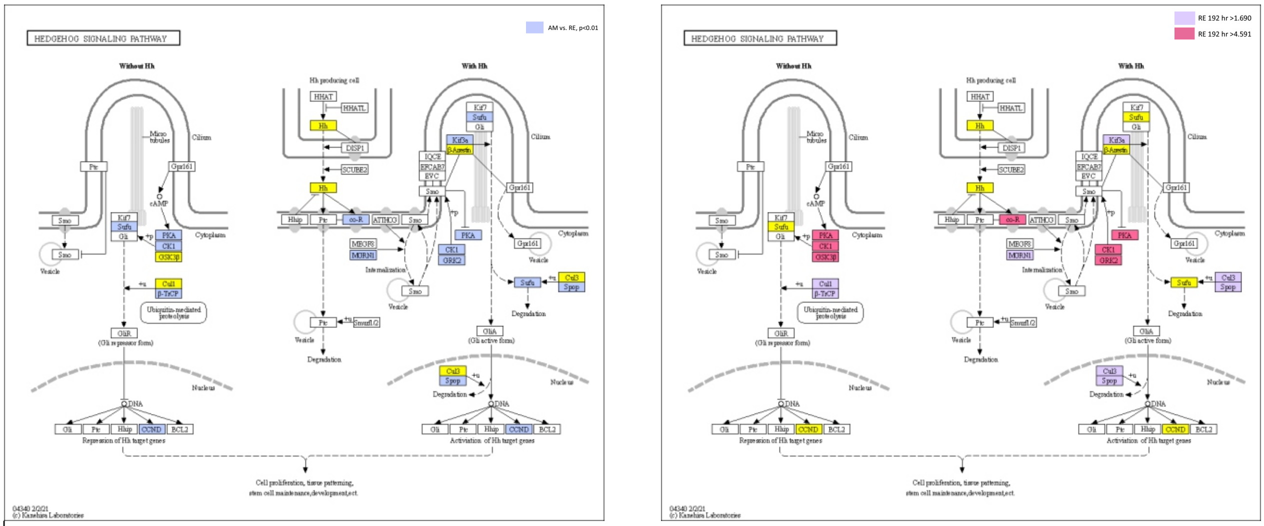

HEDGEHOG SIGNALING PATHWAY
AM vs. RE, p<0.01
RE 192 hr >1.690
RE 192 hr >4.591


D

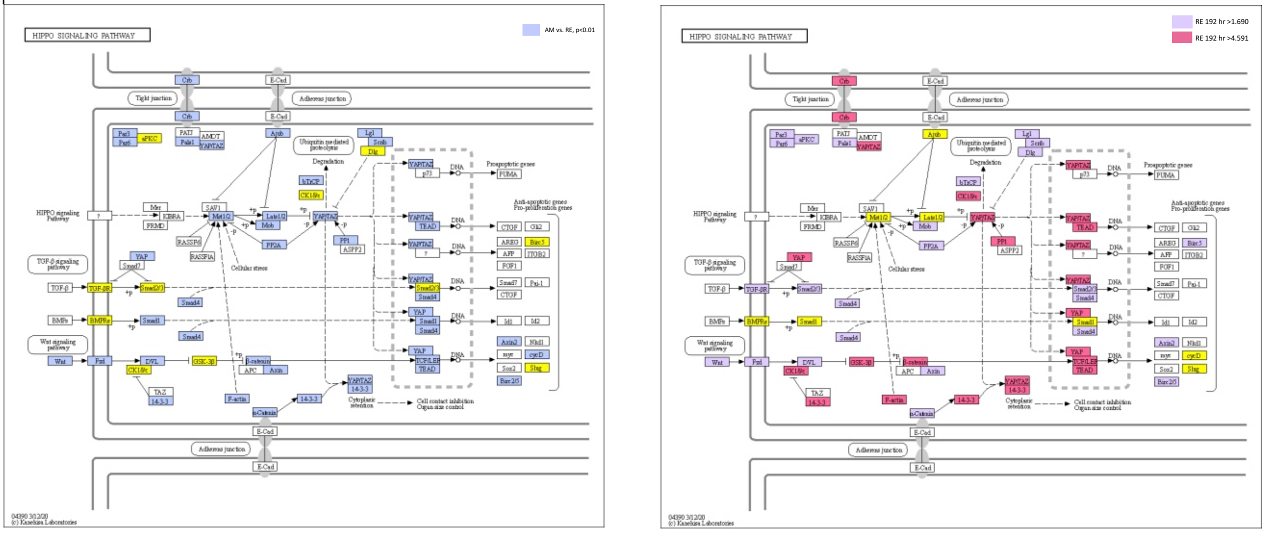

HIPPO SIGNALING PATHWAY
AM vs. RE, p<0.01
RE 192 hr >1.690
RE 192 hr >4.591

Fig S5

E

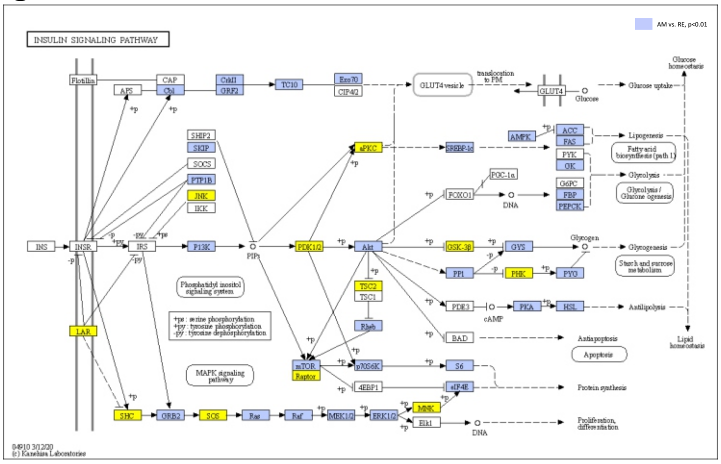
INSULIN SIGNALING PATHWAY
AM vs. RE, p<0.01

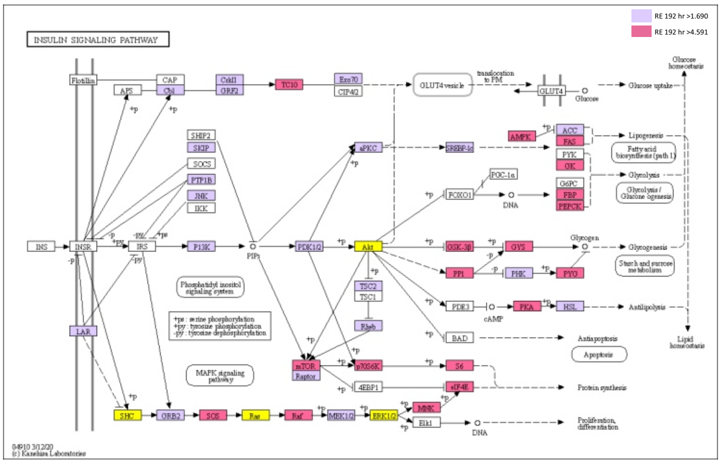
INSULIN SIGNALING PATHWAY
RE 192 hr >1.690
RE 192 hr >4.591

F

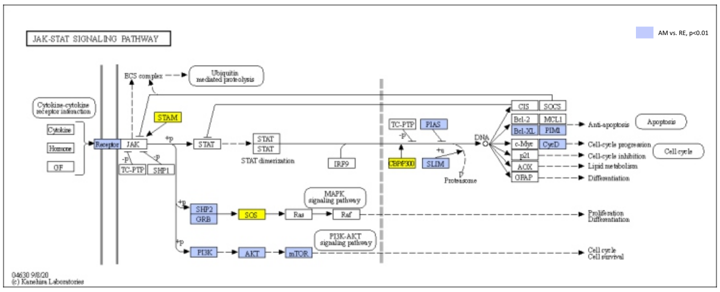
JAK-STAT SIGNALING PATHWAY
AM vs. RE, p<0.01

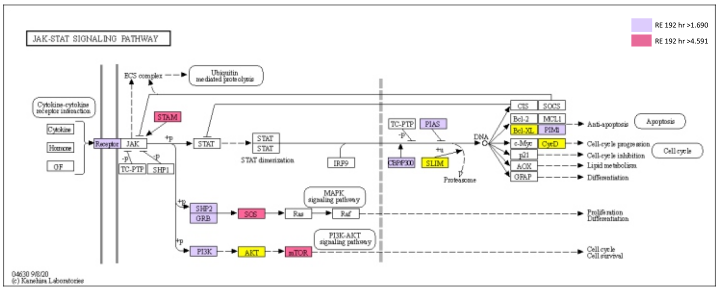
JAK-STAT SIGNALING PATHWAY
RE 192 hr >1.690
RE 192 hr >4.591

G

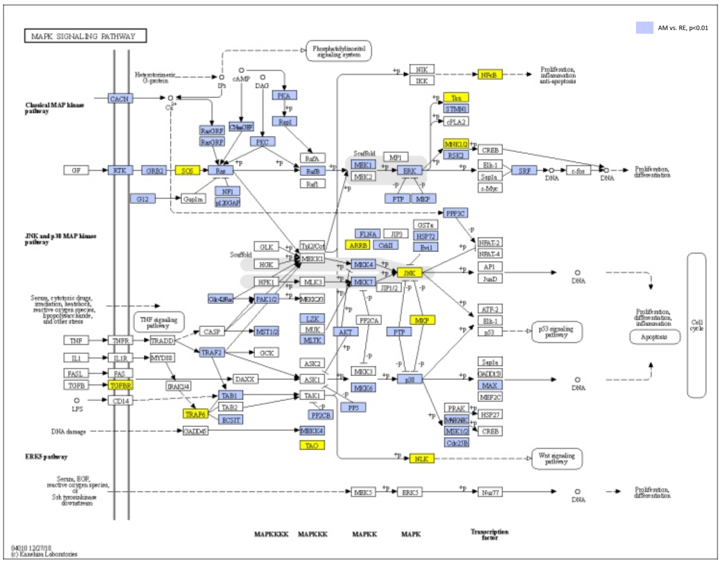
MAPK SIGNALING PATHWAY
AM vs. RE, p<0.01

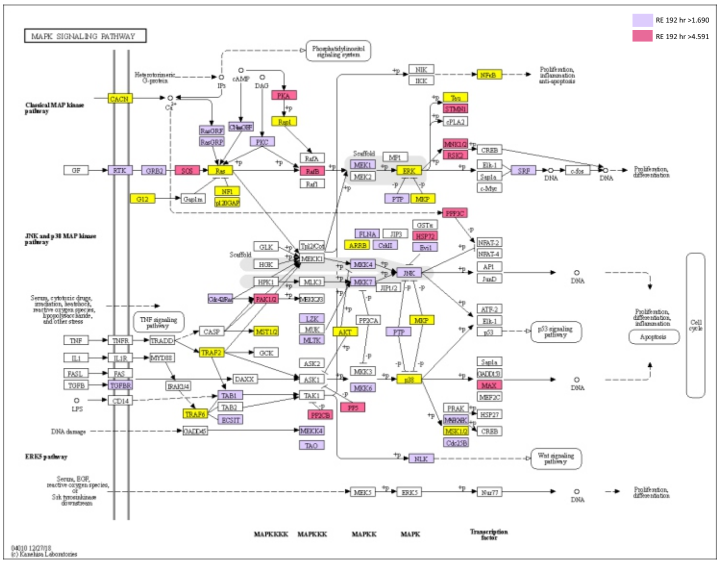
MAPK SIGNALING PATHWAY
RE 192 hr >1.690
RE 192 hr >4.591

H

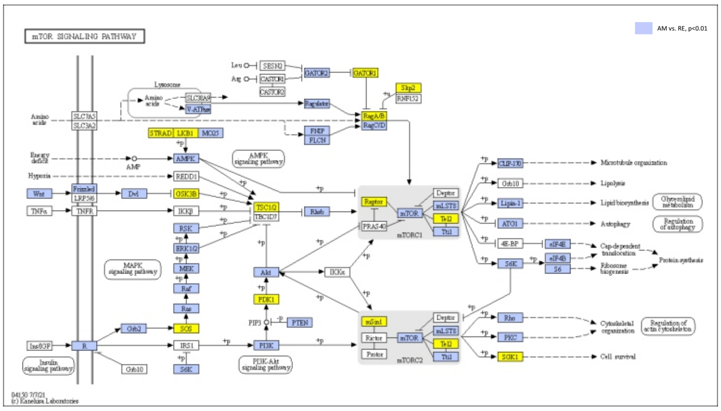
mTOR SIGNALING PATHWAY
AM vs. RE, p<0.01

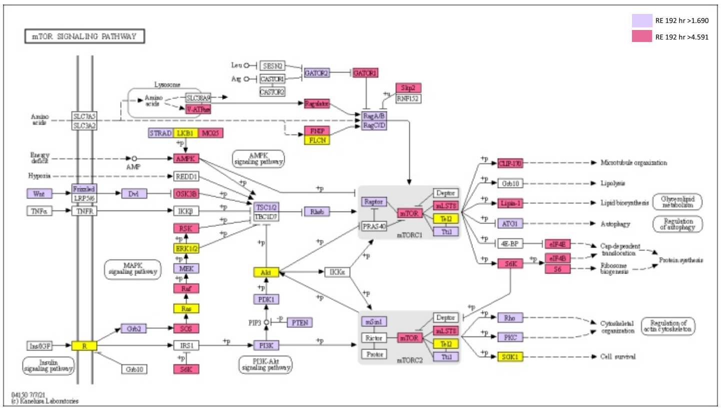
mTOR SIGNALING PATHWAY
RE 192 hr >1.690
RE 192 hr >4.591

Fig S5

I

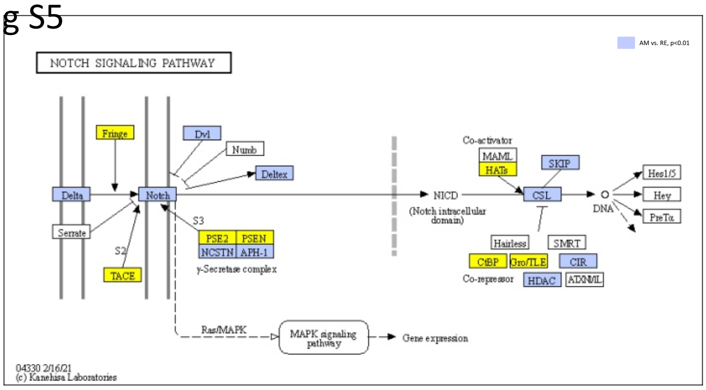
NOTCH SIGNALING PATHWAY
AM vs. RE, p<0.01
04330 2/16/21
(c) Kanehisa Laboratories

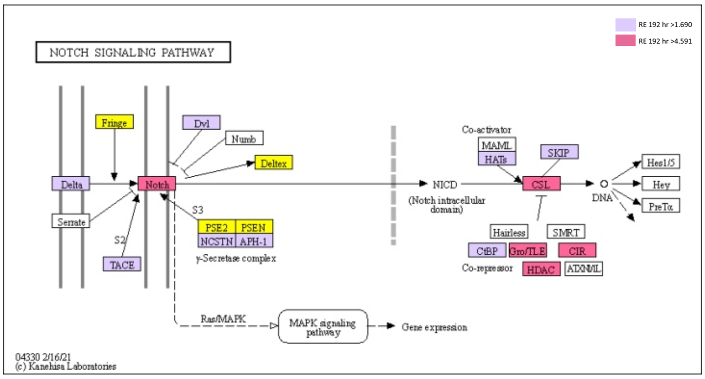
NOTCH SIGNALING PATHWAY
RE 192 hr >1.690
RE 192 hr >4.591
04330 2/16/21
(c) Kanehisa Laboratories

J

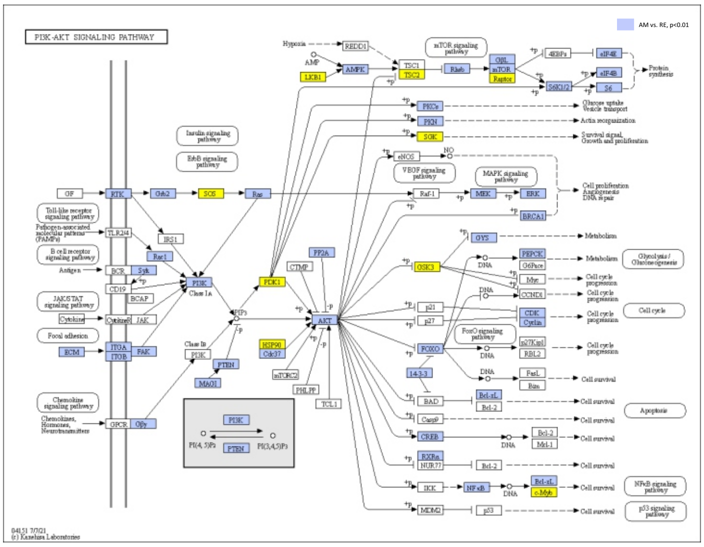
PI3K-AKT SIGNALING PATHWAY
AM vs. RE, p<0.01

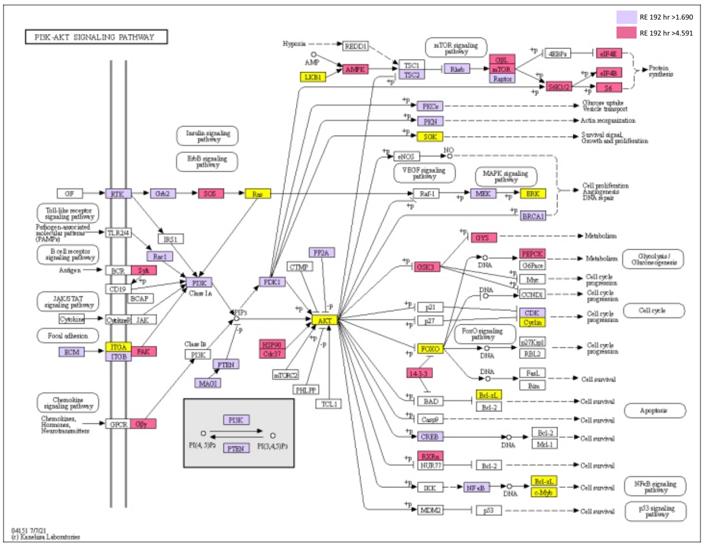
PI3K-AKT SIGNALING PATHWAY
RE 192 hr >1.690
RE 192 hr >4.591

K

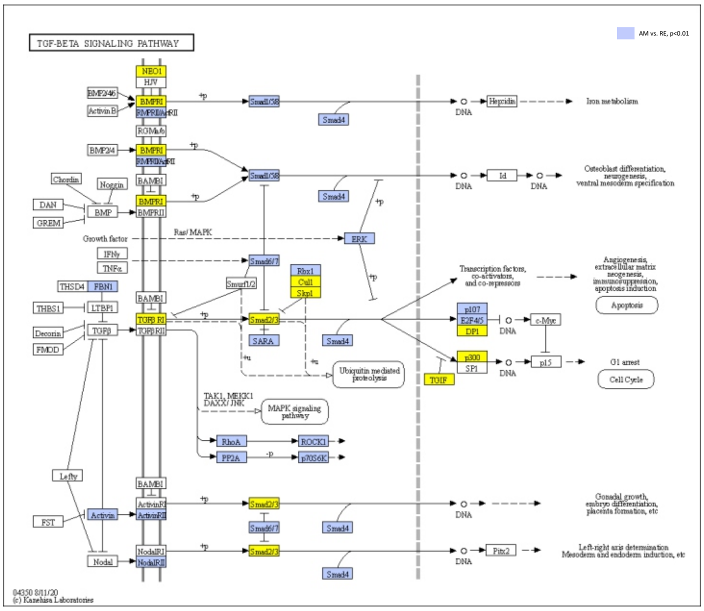
TGF-BETA SIGNALING PATHWAY
AM vs. RE, p<0.01
04350 8/11/20
(c) Kanehisa Laboratories

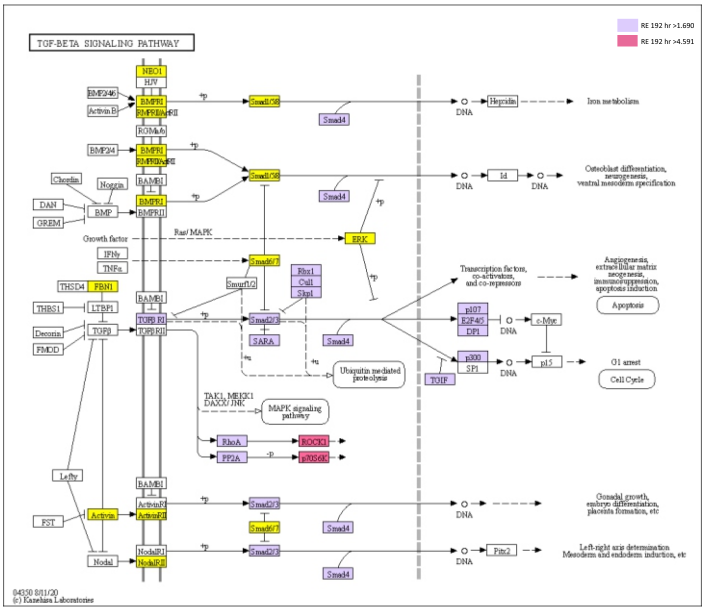
TGF-BETA SIGNALING PATHWAY
RE 192 hr >1.690
RE 192 hr >4.591
04350 8/11/20
(c) Kanehisa Laboratories

L

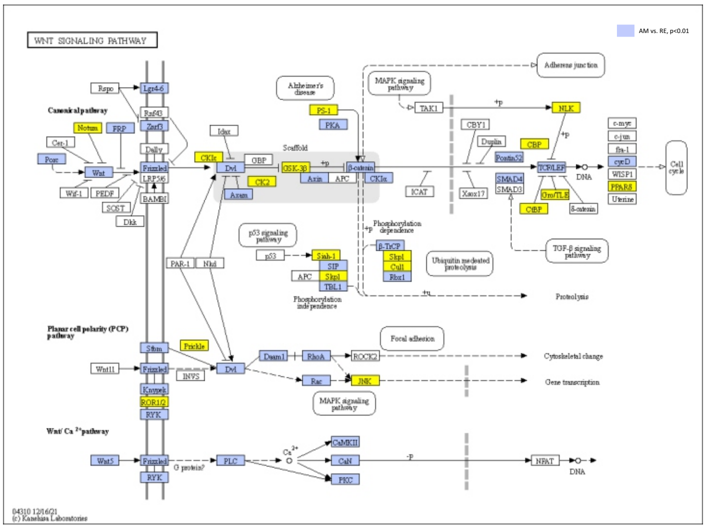
WNT SIGNALING PATHWAY
AM vs. RE, p<0.01
04310 12/16/21
(c) Kanehisa Laboratories

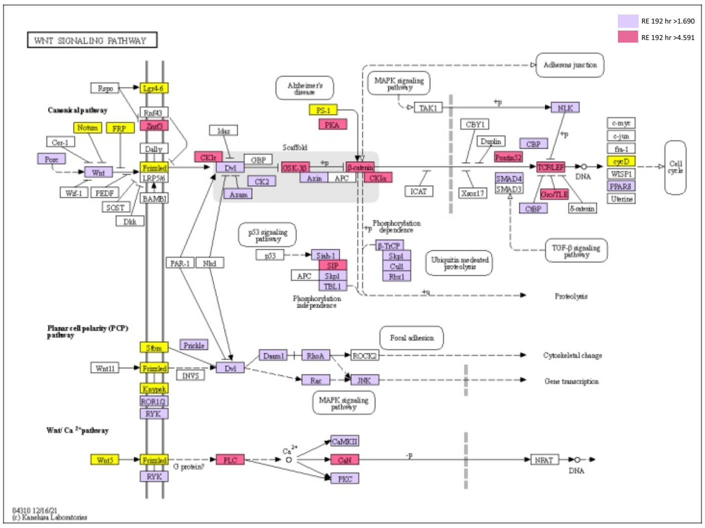
WNT SIGNALING PATHWAY
RE 192 hr >1.690
RE 192 hr >4.591
04310 12/16/21
(c) Kanehisa Laboratories
