# Supplementary material for: A transcriptomic examination of encased rotifer embryos reveals the developmental trajectory leading to long-term dormancy; are they “animal seeds”?
Source: BMC Genomics. 2024 Jan 27;25:119. doi: 10.1186/s12864-024-09961-1 (PMC10821554; doi:10.1186/s12864-024-09961-1)
Supplement: Supplementary file 4 — Additional file 4: Figure. S1. Number of reads sequenced per sample across three REs and AMs developmental stages. S2 Fig. Comparison of the transcript abundance profiles of putative maternal genes between AMs and REs. S3 Fig. The Longevity pathway (worm) highlights differential transcript abundance between AM and RE (left panel) and highly abundant protein-encoding genes at 192 h in RE (right panel). S4 Fig. Very highly abundant gene transcripts (>4.251) of energy-yielding KEGG pathways. (>4.251) at 192 hr of RE. S5 Fig. KEGG signaling pathways (maps) highlighting protein-encoding genes with differential transcript abundance between AM and RE (left panel) and highly abundant transcripts at 192 hr in RE (right panel). S6 Fig. Lipid metabolism KEGG pathways (maps) highlighting protein-encoding genes with differential transcript abundance between AM and RE (left panels) and highly abundant transcripts at 192 hr in RE (right panel). S7 Fig. Light responding KEGG pathways (maps) highlighting protein-encoding genes with differential transcript abundance between AM and RE (left panels) and transcripts with high abundance at 192 hr in RE (right panels). [file 12864_2024_9961_MOESM4_ESM.zip › Additional File 4, S6 Fig.pdf]

Additional File 4

S6 Fig. pdf: **Lipid metabolism KEGG pathways (maps) highlighting protein-encoding genes with differential transcript abundance between AM and RE (left panels) and highly abundant transcripts at 192 hr in RE (right panel).** The pathways include (**A**) fatty acid biosynthesis, (**B**) fatty acid elongation, (**C**) biosynthesis of unsaturated fatty acids, and (**D**) fatty acid degradation. Blue colored boxes in the left panels of A, B, and D, represent transcripts with differential abundance between AM and RE (AM vs. RE, $p<0.01$). In the right panel, dark-colored mauve boxes represent protein-encoding gene transcripts with values >4.951, while light-colored mauve boxes represent protein-encoding gene transcripts with values >1.690. Yellow boxes represent gene transcripts identified in Additional File 2, S2A Table but not in one of the patterns described above, while blank boxes represent protein-encoding genes that are present in the KEGG map but were not identified in Additional File 2, S2A Table. In Panel C, blue arrows in the left panel represent transcripts with differential abundance between AM and RE (AM vs. RE, $p<0.01$). In the right panel dark-colored mauve boxes represent protein-encoding gene transcripts with values >4.951, while light-colored mauve arrows represent protein-encoding gene transcripts with values >1.690.

Fig S6

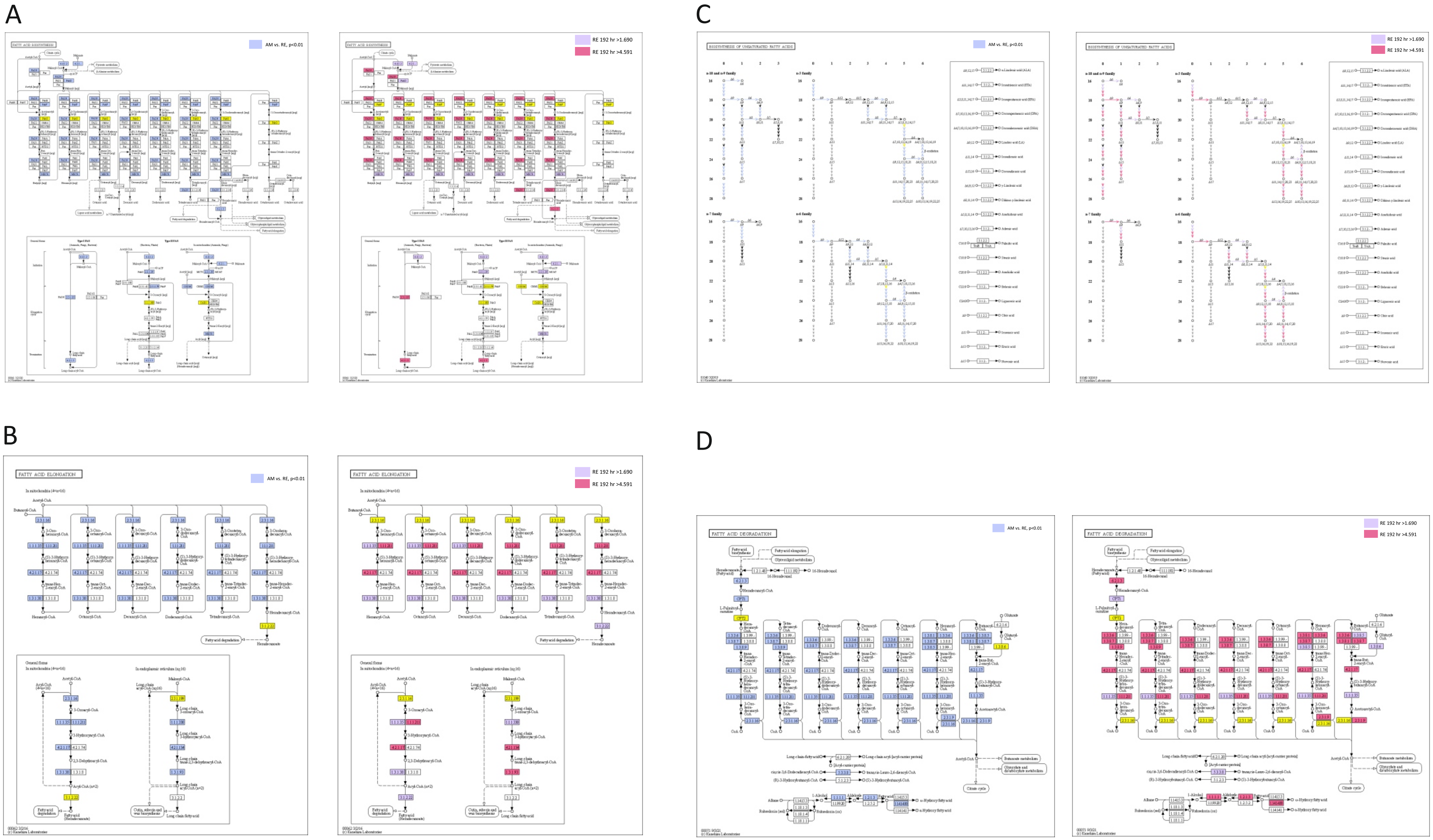
A
B
C
D
FATTY ACID BIOSYNTHESIS
FATTY ACID ELONGATION
BIOSYNTHESIS OF UNSATURATED FATTY ACIDS
FATTY ACID DEGRADATION
AM vs. RE, p<0.01
RE 192 hr >1.690
RE 192 hr >4.591
