# Supplementary material for: A transcriptomic examination of encased rotifer embryos reveals the developmental trajectory leading to long-term dormancy; are they “animal seeds”?
Source: BMC Genomics. 2024 Jan 27;25:119. doi: 10.1186/s12864-024-09961-1 (PMC10821554; doi:10.1186/s12864-024-09961-1)
Supplement: Supplementary file 4 — Additional file 4: Figure. S1. Number of reads sequenced per sample across three REs and AMs developmental stages. S2 Fig. Comparison of the transcript abundance profiles of putative maternal genes between AMs and REs. S3 Fig. The Longevity pathway (worm) highlights differential transcript abundance between AM and RE (left panel) and highly abundant protein-encoding genes at 192 h in RE (right panel). S4 Fig. Very highly abundant gene transcripts (>4.251) of energy-yielding KEGG pathways. (>4.251) at 192 hr of RE. S5 Fig. KEGG signaling pathways (maps) highlighting protein-encoding genes with differential transcript abundance between AM and RE (left panel) and highly abundant transcripts at 192 hr in RE (right panel). S6 Fig. Lipid metabolism KEGG pathways (maps) highlighting protein-encoding genes with differential transcript abundance between AM and RE (left panels) and highly abundant transcripts at 192 hr in RE (right panel). S7 Fig. Light responding KEGG pathways (maps) highlighting protein-encoding genes with differential transcript abundance between AM and RE (left panels) and transcripts with high abundance at 192 hr in RE (right panels). [file 12864_2024_9961_MOESM4_ESM.zip › Additional File 4, S7 Fig.pdf]

Additional File 4

S7 Fig. pdf: **Light responding KEGG pathways (maps) highlighting protein-encoding genes with differential transcript abundance between AM and RE (left panels) and transcripts with high abundance at 192 hr in RE (right panels).** The pathways include (**A**) phototransduction (**B**) circadian rhythm, and (**C**) circadian entrainment. Blue colored boxes in the left panel represent transcripts with differential abundance between AM and RE (AM vs. RE, $p<0.01$). In the right panel, dark-colored mauve boxes represent protein-encoding gene transcipts with values >4.951, while light-colored mauve boxes represent protein-encoding gene transcripts with values >1.690. Yellow boxes represent gene transcripts identified in Additional File 2, S2A Table but not in one of the patterns described above, while blank boxes represent protein-encoding genes that are present in the KEGG map but were not identified in Additional File 2, S2A Table.

Fig S7

A

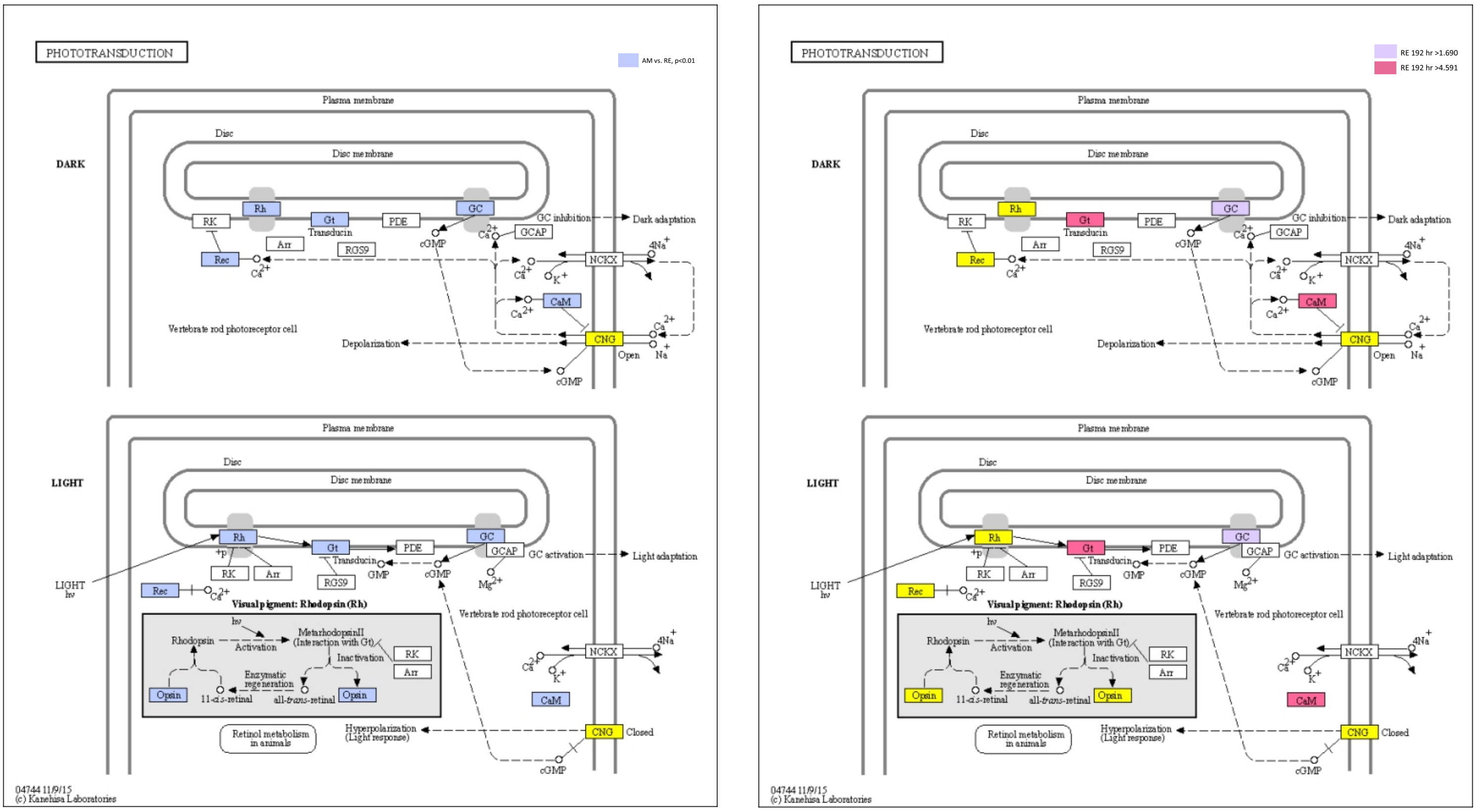
PHOTOTRANSDUCTION
AM vs. RE, p<0.01
RE 192 hr >1.690
RE 192 hr >4.591
Plasma membrane
Disc
Disc membrane
DARK
LIGHT
Rh
Gt
Transducin
PDE
GC
RK
Arr
RGS9
Rec
GCAP
NCKX
CaM
CNG
GC inhibition
Dark adaptation
GC activation
Light adaptation
Vertebrate rod photoreceptor cell
Depolarization
Hyperpolarization (Light response)
Open
Closed
cGMP
GMP
Visual pigment: Rhodopsin (Rh)
Rhodopsin
MetarhodopsinII (Interaction with Gt)
Activation
Inactivation
Enzymatic regeneration
Opsin
11-cis-retinal
all-trans-retinal
Retinol metabolism in animals
04744 11/9/15
(c) Kanehisa Laboratories

B

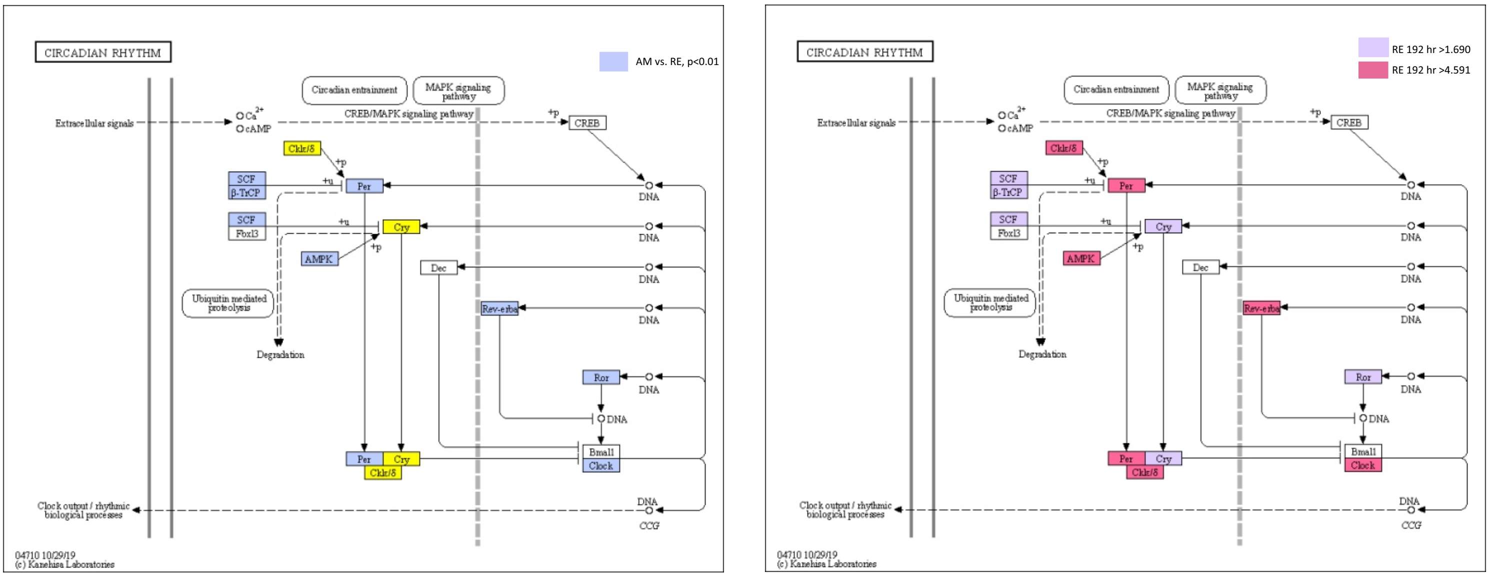
CIRCADIAN RHYTHM
AM vs. RE, p<0.01
RE 192 hr >1.690
RE 192 hr >4.591
Circadian entrainment
MAPK signaling pathway
Extracellular signals
CREB/MAPK signaling pathway
CREB
Ckle/δ
SCF β-TrCP
SCF Fbxl3
Per
Cry
AMPK
Dec
Rev-erbα
Ror
Bmal1
Clock
DNA
Ubiquitin mediated proteolysis
Degradation
Clock output / rhythmic biological processes
CCG
04710 10/29/19
(c) Kanehisa Laboratories

C

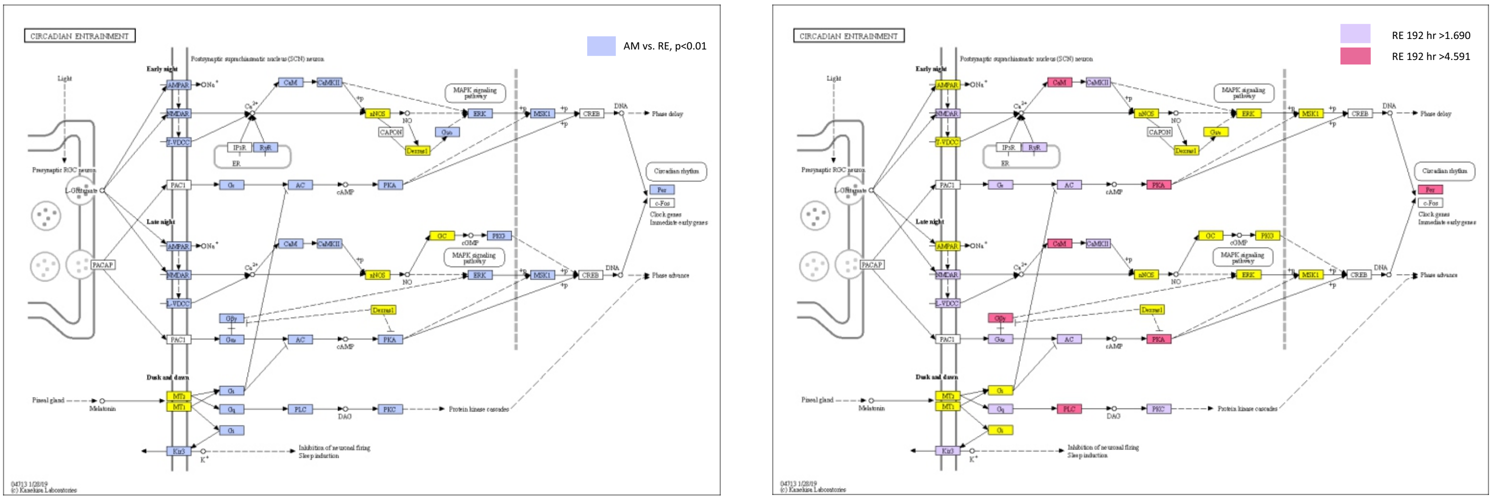
CIRCADIAN ENTRAINMENT
AM vs. RE, p<0.01
RE 192 hr >1.690
RE 192 hr >4.591
Early night
Late night
Dusk and dawn
Light
Glutamate
PACAP
Melatonin
Pineal gland
Phase delay
Phase advance
Circadian rhythm
04713 10/28/19
(c) Kanehisa Laboratories
